# Context-aware experience sampling reveals the scale of variation in affective experience

**DOI:** 10.1038/s41598-020-69180-y

**Published:** 2020-07-27

**Authors:** Katie Hoemann, Zulqarnain Khan, Mallory J. Feldman, Catie Nielson, Madeleine Devlin, Jennifer Dy, Lisa Feldman Barrett, Jolie B. Wormwood, Karen S. Quigley

**Affiliations:** 1 Northeastern University, Boston, USA; 2 University of North Carolina at Chapel Hill, Chapel Hill, USA; 3 Martinos Center for Biomedical Imaging, Massachusetts General Hospital, Charlestown, USA; 4 University of New Hampshire, Durham, USA; 5 Edith Nourse Rogers Memorial Veterans Hospital, Bedford, USA

**Keywords:** Electrophysiology, Psychology, Human behaviour

## Abstract

Emotion research typically searches for consistency and specificity in physiological activity across instances of an emotion category, such as anger or fear, yet studies to date have observed more variation than expected. In the present study, we adopt an alternative approach, searching inductively for structure within variation, both within and across participants. Following a novel, physiologically-triggered experience sampling procedure, participants’ self-reports and peripheral physiological activity were recorded when substantial changes in cardiac activity occurred in the absence of movement. Unsupervised clustering analyses revealed variability in the number and nature of patterns of physiological activity that recurred within individuals, as well as in the affect ratings and emotion labels associated with each pattern. There were also broad patterns that recurred across individuals. These findings support a constructionist account of emotion which, drawing on Darwin, proposes that emotion categories are populations of variable instances tied to situation-specific needs.

## Introduction

Scientists who study emotion acknowledge that there is variability in the *expression* of emotion across individuals and cultures, but differ in whether they view this variability as an intrinsic or epiphenomenal property of emotion (see supplementary Figure S1). Classical views of emotion, for example, propose that there is a single pattern of autonomic nervous system (ANS) activity that is diagnostic of each emotion category (e.g., anger, fear), and consider variability to be epiphenomenal to emotion. In these views, variability is assumed to derive from regulatory processes, display rules, an inability to sample intense emotional events in the lab, and/or stochastic noise^1–6^. Prototype views of emotion propose that ANS activity varies across instances of the same emotion category, but it is still assumed that each category has a diagnostic (i.e., typical or frequent) pattern that can be used to consistently and specifically identify its instances (consonant with several theorists’ work^7–9^). We refer to the idea of an emotion-specific diagnostic pattern as a *fingerprints hypothesis*^10^. This hypothesis is often assumed by basic emotion and causal appraisal approaches to emotion^1,3,6,7,9^. Real fingerprints vary from one instance to the next depending on a host of variables (such as the amount of sweat, pressure or surfaces touched) but are presumed to be sufficiently unique to identify a person across instances. In the same way, if the ‘fear’ fingerprint were an increase in heart rate and electrodermal (i.e., sweat gland) activity and a decrease in skin temperature^11^, then this fingerprint may not be present in full for every instance of fear. However, it would be present in many instances of fear both within people across situations and across persons, and it would not be observed at levels above chance in instances of any other emotion category (such as anger or happiness).

Similarly, multiple scientists have proposed that emotion categories are uniquely associated with the affective features of valence and arousal, implying that emotion categories also have *affective* fingerprints^7,9,12,13^. From these perspectives, for example, the experience of fear would be consistently and specifically associated with negative, high arousal affect, and would not be associated with pleasant or low arousal affect at levels above chance. Furthermore, by these accounts, affective features should have diagnostic biological correlates, such that experienced pleasantness and activation should be consistently and specifically associated with patterns of biological activity.

Alternatively, other theoretical views in the science of emotion employ a *populations hypothesis*, proposing that each emotion category is a population of context-dependent instances whose features, including ANS features, vary in a situated fashion. This hypothesis has been proposed by constructionist, behavioral ecological, and functionalist approaches to emotion. These views hypothesize, a priori, that there is substantial within-category variation as well as between-category similarity that far exceeds what is accounted for by a classical or prototype approach^14–20^. A populations hypothesis draws on Darwin’s discovery of population thinking^21^, in which a biological category, such as a species, consists of heterogeneous individuals. Biological categories do not represent discrete physical types (e.g., consistent and specific patterns of features), and in fact, ‘ideal’ instances of categories (i.e., their prototypes) are statistical summaries that need not exist in nature^22^. Accordingly, these categories are conceptual: they are collections of variable instances that are treated as similar for some function or purpose^16–18^. A populations view of emotions thus posits that emotions, as biological categories, consist of heterogeneous instances which vary in their physiological and psychological features, but which are treated as similar for some function or purpose.

With regard to affect, a populations hypothesis proposes that valence and arousal are features of consciousness that can vary within an emotion category^23,24^. For example, the experience of fear can be affectively pleasant, such as when riding a roller coaster. Critically, a populations hypothesis proposes—and past research has demonstrated—that both valence^25,26^ and arousal^27^ can vary in their biological correlates.

Consistent with a populations hypothesis, a recent meta-analysis found considerable variability in patterns of peripheral physiological activity within emotion categories as well as considerable similarities across categories^10^, replicating prior meta-analyses^28,29^ and demonstrating that ANS patterns for emotion categories are neither consistent nor specific. These findings parallel results showing a lack of consistency and specificity in other biological measures taken during instances of emotion, including facial muscle movements^30^ and neural correlates—whether measured at the level of individual neurons^31^, as activity in specific brain regions^23,32,33^, as activity or connectivity in brain networks^34,35^, or as distributed patterns of activity^36^. Despite the accumulating evidence in support of a populations hypothesis, however, contemporary research continues to be designed with the assumptions of a fingerprints hypothesis in mind^37,38^.

Meta-analytic findings, while consistent with the populations hypothesis, cannot rigorously test it; meta-analytic approaches rely on merely summarizing data across people and situations rather than designing studies to specifically assess intra-individual and inter-individual variability across instances. Moreover, standard lab-based emotion induction methods limit the intensity of the emotional instances sampled^5^, and thus may under-estimate context-specific variation in the physiological features of emotional instances. In the present study, we used a more idiographically-sensitive method that provided us with the opportunity to observe and characterize the extent of physiological variability within and across emotion categories in real-world situations^39^. Previous studies that have attempted to observe within-category variation using experience-sampling in daily life have found evidence supporting the populations hypothesis. For example, an analysis of momentary reports of experienced affect found that the relationship between valence and arousal varied widely across individuals and situations^40^. Experience sampling approaches are often employed to assess self-report features of experience in natural settings^41,42^, yet this work has only just begun to integrate ambulatory monitoring of peripheral physiology^39,43,44^. Research that collects both self-report and physiological features of experience in everyday life is therefore a critical next step in testing a populations hypothesis.

Our method—a novel, physiologically-triggered experience-sampling approach—was specifically designed to sample evocative moments defined in a biological way. Participants were prompted to report on their affective experience upon substantial, sustained changes in their cardiac activity in the absence of physical movement or posture change. By triggering experience samples in this way, we were able to efficiently obtain self-reports when individuals evidenced greater physiological change, and when those changes were most likely due to greater psychological salience rather than gross bodily movement. We then employed an unsupervised machine learning approach to discover the number of distinct, frequently repeated patterns of physiological activity within each individual, and we assessed whether any of these patterns recurred across participants. We also assessed the extent to which any of these patterns were associated with participant-provided labels for emotional experience and/or affective features. This approach allowed us to investigate the relationship between physiological reactivity and emotion categories within individuals in a data-driven way.

As part of a larger study on affective experience and decision making in daily life, 52 participants completed approximately 14, eight-hour days of ambulatory peripheral physiological monitoring. Each day, participants visited the lab and were outfitted with sensors and portable equipment to measure their electrocardiogram (ECG) and impedance cardiogram (ICG) as well as bodily movement and posture (via accelerometers). A custom-built smartphone application initiated an experience sampling prompt any time the interbeat interval (IBI; also called heart period) changed by more than ± 167 ms over an eight-second period, with these thresholds adjusted per participant to ensure they received a comparable number of prompts per day. Prompts were not generated if participants had moved substantially or shifted posture within the proceeding 30 s. In response to each experience sampling prompt, participants first rated their felt valence and arousal on 100-point continuous slider scales and then freely labeled their current affective experience. Confirming that our approach helped us to target evocative affective experiences in everyday life, we found that experience sampling events included ratings for valence (*M* = 10.16, *SD* = 17.43) and arousal (*M* = 6.40, *SD* = 19.42) that each covered the entire possible range from -50 to + 50. To ensure comparability of results across the sample, we excluded participants who had fewer than 70 events devoid of major physiological artifact. This resulted in the removal of six participants, resulting in a final sample size of 46 (see “Methods” for participant demographics).

Using the ambulatory physiological data, we derived six cardiovascular features for analysis (Table 1): interbeat interval (IBI), respiratory sinus arrhythmia (RSA), pre-ejection period (PEP), left ventricular ejection time (LVET), stroke volume (SV), and cardiac output (CO). These cardiovascular features are frequently used in the motivated performance literature, making them highly relevant to sampling evocative experiences in-the-world^45–48^. Change scores for each feature were computed for each experience sampling event, as the difference in physiological activity between the 30 s preceding the IBI change that initiated the experience sampling prompt and the 30 s following (see supplementary Figure S2). Change scores for each participant were submitted to a within-person clustering analysis using Dirichlet Process-Gaussian Mixture Modeling (DP-GMM)^49–51^. Each data point in the model represented a single experience sampling event and was characterized by a six-dimensional vector of within-person standardized change scores for IBI, RSA, PEP, LVET, SV, and CO. The model for each participant yielded a solution comprising clusters of increasingly smaller size, until remaining data represented clusters of single events. Rather than assign each event to only one cluster, DP-GMM assigns and weights cluster membership probabilistically. Thus, each event could be associated with more than one cluster, and contributed proportionally to calculations of weighted mean change scores for the six physiological features across multiple clusters.

**Table 1:** Cardiovascular features derived from ambulatory physiological data.

Feature	Definition	Interpretation
Interbeat interval (IBI)	Time (in ms) between heartbeats (inverse of heart rate)	IBI describes how fast the heart is beating; greater IBI values denote a slower heart rate
Respiratory sinus arrhythmia (RSA)	High frequency variability in IBI which occurs at the respiratory frequency	RSA is an estimate of parasympathetic (PNS) influence on the heart; greater RSA values typically indicate PNS activation
Pre-ejection period (PEP)	Time (in ms) between the beginning of electrical stimulation of the heart and the opening of the aortic valve	PEP is an inverse estimate of cardiac contractility and sympathetic (SNS) control of the heart; greater PEP values typically indicate reduced contractility and SNS withdrawal
Left ventricular ejection time (LVET)	Time (in ms) between the opening and closing of the aortic valve	LVET describes how long it takes the heart to pump blood out of the heart on a given heartbeat; greater LVET values are associated with greater time to eject blood per heartbeat
Stroke volume (SV)	Volume (in mL) of blood ejected by the heart with each beat	SV describes the volume of blood ejected from the heart during each heartbeat; greater SV values indicate greater blood volume per heartbeat
Cardiac output (CO)	Volume (in L) of blood circulated in the body per unit of time (min)	CO describes blood flow over time; greater CO values indicate greater blood flow rate (in L/min)

Consistent with a populations hypothesis, we predicted that participants would vary in the number of clusters discovered in their data, and that these clusters would represent diverse patterns of change in physiological activity both within and across participants. However, we also predicted that we would find structure within this variation, such that we would find some patterns of change in physiological activity that broadly recurred across participants. We further predicted a many-to-many relationship between clusters (i.e., recurring patterns of change in physiological activity) and the words and affect ratings used to label events in those clusters. Conversely, support for a fingerprints hypothesis would be evidenced by similar patterns of change in physiological activity within and across participants, with each cluster consistently and specifically associated with particular emotion words or valence and/or arousal ratings.

## Results

### Variation in patterns of change in physiological activity within participants

We examined the number of clusters of physiological activity that accounted for at least five percent of the within-person events submitted for analysis, along with the percentage of events for which physiological data were not included in clusters of this size (i.e., those left unclustered). In total, 219 clusters of physiological activity were identified across participants, with a mean of 4.76 clusters per person (*SD* = 1.25; see additional summary statistics in supplementary Table S4). The discovered clustering solutions fit the data well; events’ average probability of cluster membership was high (*M* = 0.87; *SD* = 0.06). See “Methods“ and pages 28–30 of the supplementary information for details regarding model and parameter validation. As predicted, we observed variability in the clusters of physiological change across participants, both in their number (minimum = 3; maximum = 8) and in their nature (supplementary Table S5). Clusters associated with a greater number of events typically evidenced small physiological changes. This makes sense because in everyday life we would expect to see commonly-occurring events associated with smaller physiological changes.

**Figure 1:**
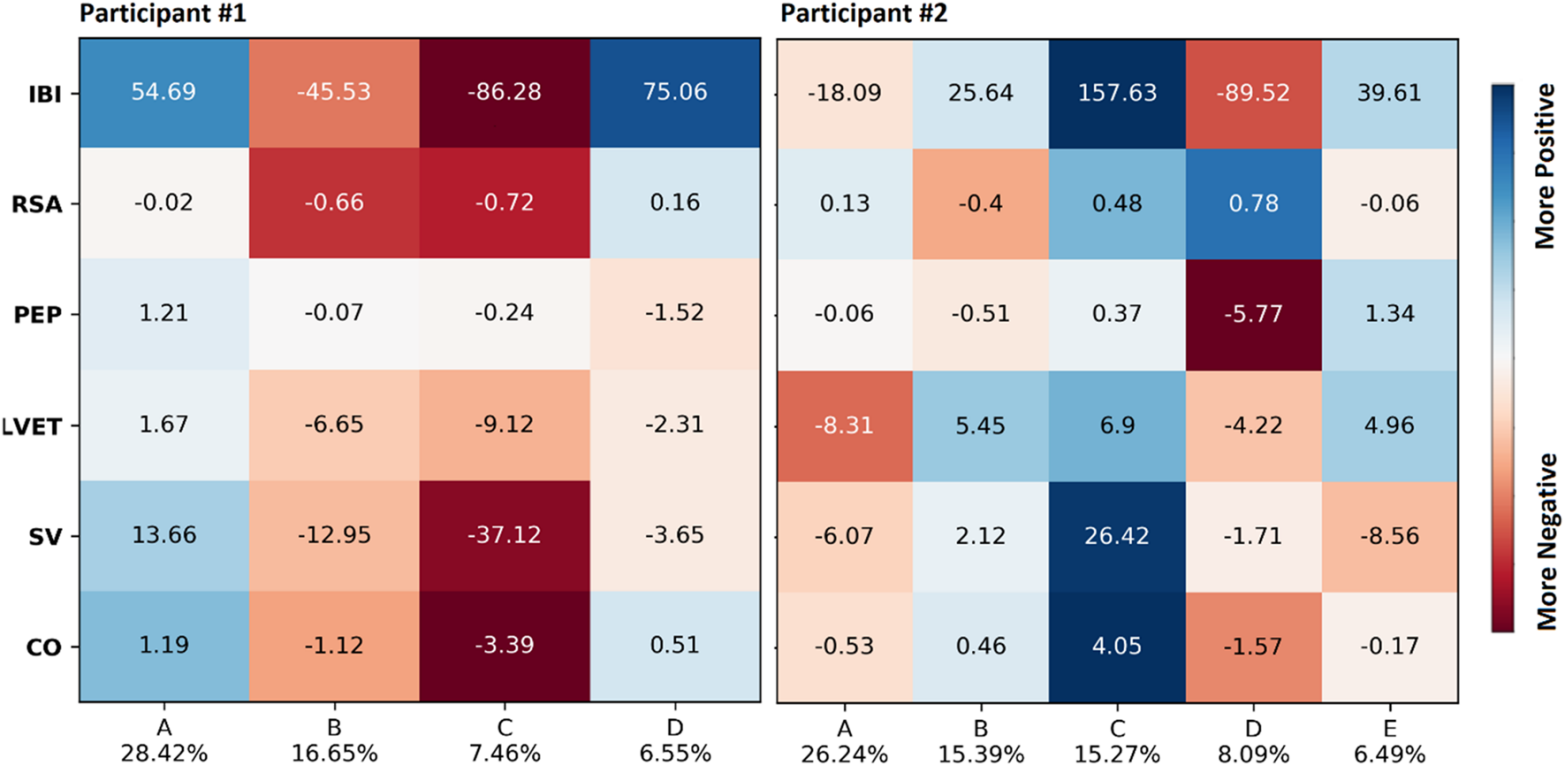
Mean weighted change scores for physiological features across identified clusters for example participants #1 (left panel) and #2 (right panel). Below the figure, each cluster is labeled with a letter and the percentage of that participant’s events that the cluster includes. Physiological features are along the vertical axis. The numbers in each cell are the raw, weighted mean change score for that feature for a given cluster. Cells are colored based on within-person effect size estimates (mean change relative to within-person standard deviation for that feature) on a continuum from red (more negative changes) to blue (more positive changes). *Participant #1:* in cluster A, the heart rate slows modestly (i.e., IBI lengthens) and overall blood flow increases modestly (i.e., CO increases); in cluster B, heart rate is slightly faster (i.e., IBI shortens), and blood flow and RSA decrease modestly; in cluster C, the heart rate is faster, and blood flow and RSA decrease more substantially; in cluster D, the heart rate slows, but there are few changes in other features. *Participant #2:* cluster A shows virtually no change in heart rate with slightly decreased blood volume ejected from the heart (i.e., SV decreases, LVET also decreases); cluster B shows a very slightly slowed heart rate and a slightly increased blood volume; cluster C shows a considerably slowed heart rate, and greatly increased blood flow; cluster D shows a faster heart rate, a notable increase in contractility (i.e., PEP decrease), and modestly decreased blood flow; cluster E shows a slightly slowed heart rate, with a slight decrease in blood flow.

Figure 1 displays the findings for two participants with an average number of clusters. Participant #1′s data was described by four clusters of physiological activity, in contrast to participant #2′s data, which was described by five. For participant #1, there was limited change in sympathetic nervous system (SNS) function (i.e., change in PEP) and modest decreases or no change in parasympathetic (PNS) function across the clusters (i.e., change in RSA), whereas for participant #2, physiological changes were primarily driven by changes in SNS function (especially in clusters D and E), with little overall change in PNS function (except in cluster D where there is coactivation of the SNS and PNS).

### Mental features associated with patterns of change in physiological activity within participants

Because each event was probabilistically associated with multiple identified clusters per person, the freely labeled emotion words and valence and arousal ratings for each event were assigned weights describing their association with each identified cluster. As predicted, there was a many-to-many relationship between clusters of physiological activity and the words used to label events in those clusters. Within participants, the same emotion word was used to label instances associated with multiple clusters, and, correspondingly, multiple words were used to label events in each cluster. Table 2 lists the number of unique words used by participants over the course of experience sampling (minimum = 9, maximum = 147), the average number of per-person clusters associated with each emotion word, and the average number of words associated with each cluster per person.

**Table 2:** Summary statistics for emotion word usage.

	Number unique words	Mean number clusters per word	Mean percent clusters per word	Mean percent unique words per cluster
Mean	37.11	2.00	43.72	13.76
SD	26.57	0.38	11.96	8.42
Median	29	2.00	41.23	10.95
Min	9	1.25	22.84	3.81
Max	147	2.71	78.43	50.36
Range	138	1.46	55.59	46.55

Note: We calculated the number of unique words each participant self-generated to label their affective experience, as well as the mean number and percent of clusters for which these words were used to label events. Additionally, we calculated the weighted percentage of unique emotion words used to label events in each identified cluster.

**Figure 2:**
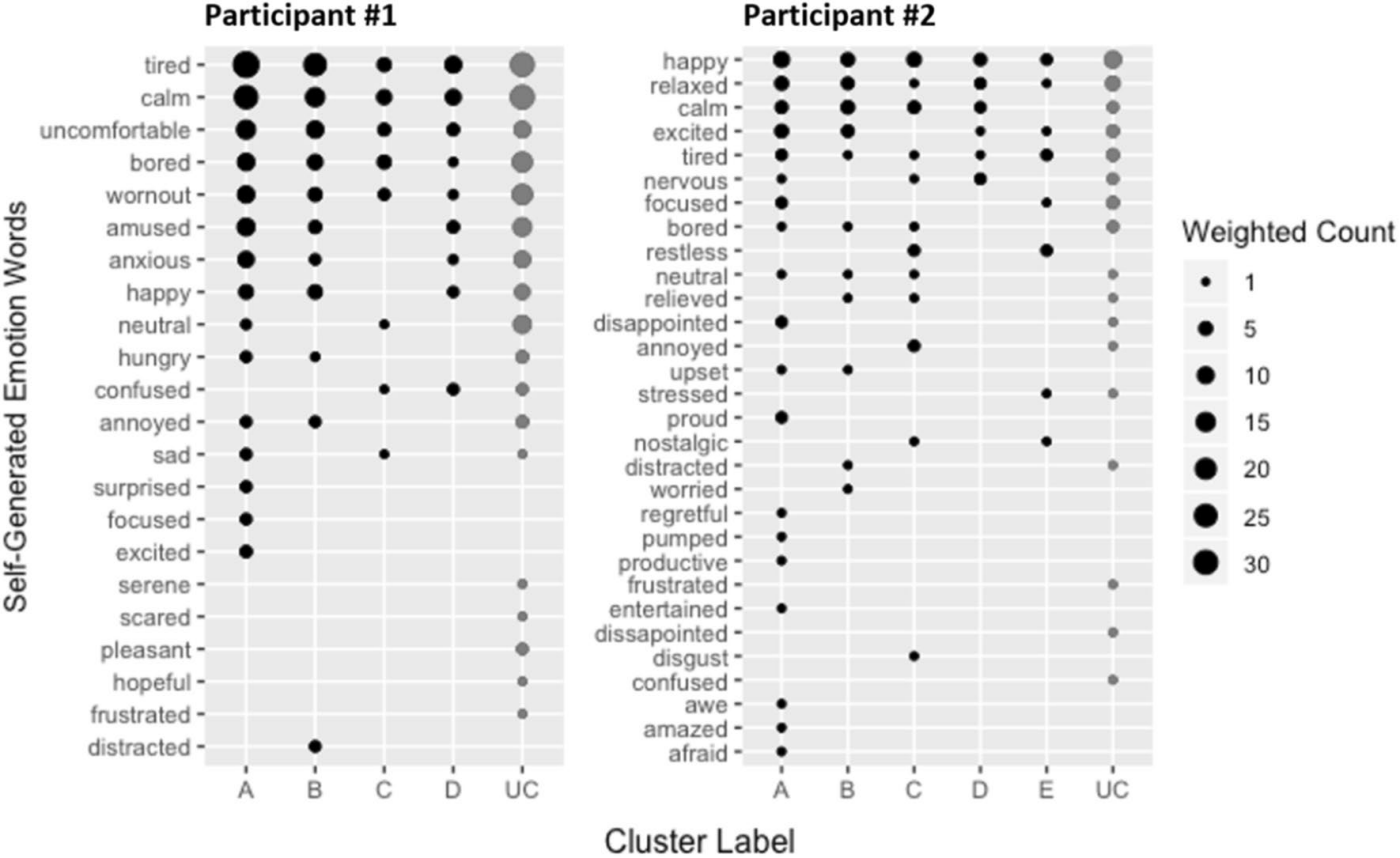
Bubble plot of unique words freely generated to label experience sampling events for example participants #1 (left panel) and #2 (right panel). Words are listed on the vertical axis in descending order of frequency. Clusters of change in physiological activity are shown on the horizontal axis, including unclustered (“UC”) events (dark gray bubbles) that belong to clusters associated with less than five percent of events. Bubbles are sized according to the weighted count of events in each cluster labeled by a given word. Specificity of word usage is illustrated when the same word does not appear across different clusters; consistency of word usage is illustrated when few words appear within a single cluster, and when these are different words than appear in other clusters. There is virtually no evidence of either specificity or consistency.

This lack of consistency and specificity in emotion word usage is well illustrated by our example participants. Participant #1 (Fig. 2; left panel) used “tired”, “calm”, “uncomfortable”, and “bored” (among other words) to refer to a substantial number of events in all four clusters (i.e., no specificity), and events in each cluster were labeled with a large number of words (i.e., low consistency). The number and identity of words used to label events varies by cluster, with cluster C associated with the fewest words. Even though most of the words in this cluster are normatively low arousal experiences (e.g., “tired”, “bored”), the normative valence^52^ of these words does vary (e.g., “calm” vs. “sad”). Participant #2 (Fig. 2; right panel) likewise displays a lack of consistency and specificity in word use. This participant used far more words than participant #1, with each word used relatively infrequently, and words like “happy”, “relaxed”, “calm”, and “excited” being used comparably across most clusters. Taken together, we see virtually no evidence of a one-to-one correspondence between physiological changes and categories of emotional experience. (Bubble plots for all participants are in supplementary Figure S4; these also show virtually no evidence of consistency or specificity.)

A similar pattern of results was observed for ratings of valence and arousal, such that there was a many-to-many relationship between clusters of physiological activity and the affective features of the events in those clusters. Across participants, grand means were large for the within-cluster ranges in valence (*M* = 45.86, *SD* = 15.06) and arousal (*M* = 47.16, *SD* = 13.90), suggesting that these affective features varied substantially within clusters. Within participants, separate tests for valence and arousal ratings confirmed that affective features overlapped across clusters. Only two participants evidenced significant differences in valence between one or more clusters (4.35% of Kruskal–Wallis one-way analysis of variance *H* values were significant at α = 0.05), and only four participants evidenced between-cluster differences in arousal (8.70% of *H* values were significant at α = 0.05). These results were not corrected for multiple comparisons, and do not exceed, or barely exceed, the number of significant differences we would expect to find by chance alone.

**Figure 3:**
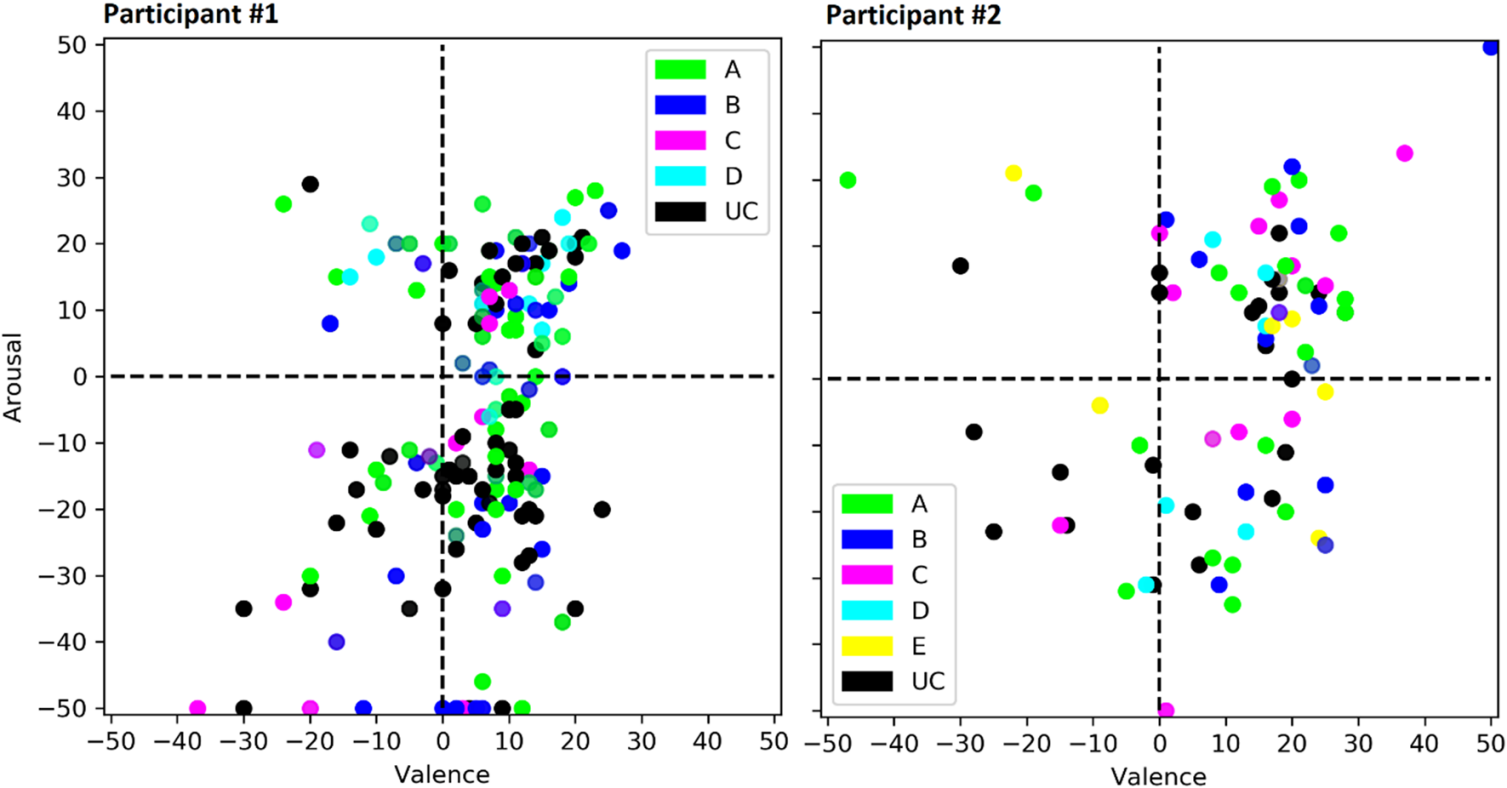
Scatter plot of affect ratings reported during experience sampling events for each cluster for example participants #1 (left panel) and #2 (right panel). Each experience sampling event is plotted as a dot, according to its weighted valence (x-axis) and arousal (y-axis), which are centered on the scale midpoints of 0. Dots are colored according to their highest probability cluster membership, with legends indicating color correspondences. Black dots (indicated in the legend with “UC”) represent unclustered events.

The lack of consistency and specificity in affective features again can be seen in our example participants. For participant #1 (Fig. 3, left panel), experience sampling events from all clusters are densely overlapped (i.e., low specificity) and associated with both positive and negative valence ratings, as well as both high and low arousal ratings (i.e., low consistency). These same observations hold for participant #2 (Fig. 3, right panel).

For comparison, we also assessed between-cluster differences in contextual features capturing type of activity, time of day, and posture. Based on previous literature, we predicted that posture (e.g., the likelihood of being seated during an event) would be more strongly related to differences in peripheral physiology^53^. As expected, we observed a greater number of significant differences in posture across clusters, and found that average effect sizes for posture comparisons were significantly larger than for those for other comparisons, including affect. See page 18 of the supplementary information and supplementary Table S6 for details.

### Common patterns of change in physiological activity across participants

To directly compare patterns of physiological activity across participants, we derived within-person effect size estimates as the mean change relative to the standard deviation for each physiological feature. Following published recommendations^54^, we then classified these effect sizes as indicating ‘negligible change’, ‘decrease’, or ‘increase’ in each feature. Using these classifications, we observed 44 broad patterns of change in physiological activity that occurred at least twice across participants, accounting for 145 or 66.21% of the 219 total clusters (see supplementary Table S7; occurrences of these patterns are also identified in supplementary Table S5′s cluster-level results). These findings suggest that clusters were not completely idiosyncratic, as is expected given the influence of physical context (i.e., most experience sampling prompts occurred while participants were sitting) as well as the constraints of human physiology (e.g., measures of cardiovascular function are not completely independent^55^). A subset of 10 patterns occurred at least five times across participants, accounting for 65 or 29.68% of the 219 total clusters, as listed in Table 3.

**Table 3:** Common patterns of change in physiological activity.

	IBI	RSA	PEP	LVET	SV	CO	Description	Freq	Rel. freq (%)
1	+	+	nc	+	+	+	PNS activation (uncoupled); slower heart rate, decreased contractility, increased blood flow	9	4.11
2	+	+	–	+	+	+	PNS and SNS activation (coactivation); slower heart rate, increased contractility, increased blood flow	8	3.65
3	–	–	nc	–	–	–	PNS withdrawal (uncoupled); faster heart rate, increased contractility, decreased blood flow	7	3.20
4	+	–	–	+	+	+	PNS withdrawal, SNS activation (reciprocal); slower heart rate, increased contractility, increased blood flow	7	3.20
5	+	–	–	nc	+	+	PNS withdrawal, SNS activation (reciprocal); slower heart rate, increased contractility, increased blood flow	7	3.20
6	+	–	nc	nc	+	+	PNS withdrawal (uncoupled); slower heart rate, increased blood flow	7	3.20
7	+	–	nc	+	+	+	PNS withdrawal (uncoupled); slower heart rate, decreased contractility, increased blood flow	5	2.28
8	+	–	nc	nc	nc	+	PNS withdrawal (uncoupled); slower heart rate, increased blood flow	5	2.28
9	+	nc	–	+	+	+	SNS activation (uncoupled); slower heart rate, increased contractility, increased blood flow	5	2.28
10	+	nc	nc	+	+	+	Negligible change in PNS/SNS activity; slower heart rate, decreased contractility, increased blood flow	5	2.28

Change score effect sizes for each feature were calculated similarly to Cohen’s *d* for a one-sample *t* test^56^ and were interpreted according to published recommendations^54^. Change score effect sizes were classified as ‘negligible change’ (nc) if they fell between − .2 and .2, ‘decrease’ (−) if they were equal to or less than −.2, and ‘increase’ ( +) if they were equal to or greater than .2.

**Figure 4:**
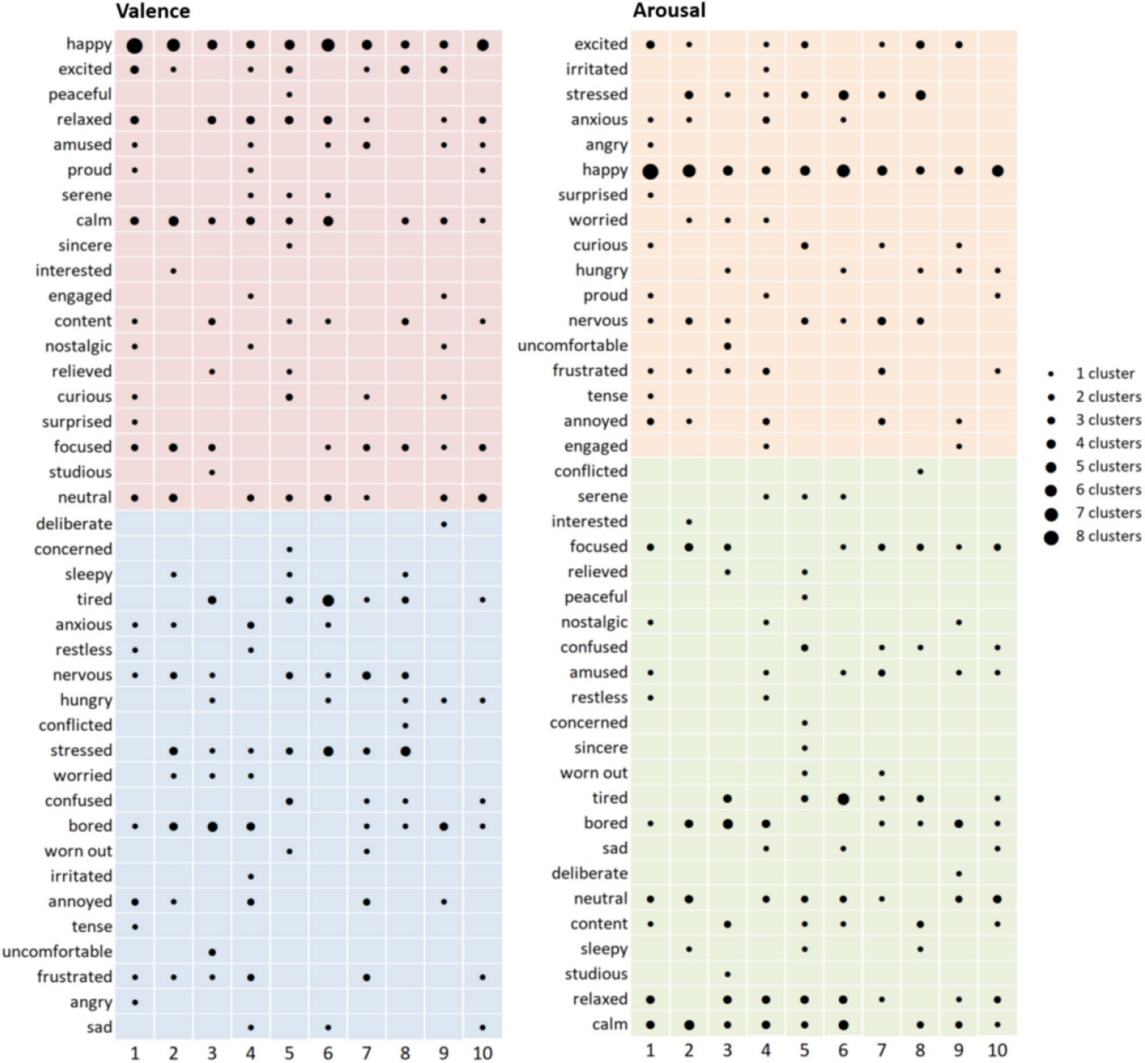
Unique words freely generated to label experience sampling events in 65 clusters corresponding to the top 10 common patterns of change in physiological activity, organized by normative valence (left panel) and arousal (right panel). Common patterns of change in physiological activity are shown on the horizontal axis; numeric identifiers correspond with common pattern descriptions in Table 3. The five most frequent words were documented for each common cluster, and then combined for all clusters matching a given pattern. Bubbles are sized according to the number of common clusters for which a given word was used. Words are listed on the vertical axis in descending order of valence and arousal ratings^52^. In the left panel, rows are shaded according to whether the word represents a normatively pleasant (pink) or unpleasant (blue) experience. In the right panel, rows are shaded according to whether the word represents a normatively high arousal (orange) or low arousal (green) experience. Both plots illustrate the many-to-many relationship between mental features and patterns of change in physiological activity.

We also examined the emotion words used to describe events in the 65 clusters representing these common patterns. Comparing emotion labels across participants in this way allowed us to assess whether mental features might be more consistently and specifically associated with more commonly recurring patterns of change in physiological activity. We found that the same words were used to label clusters associated with different physiological patterns (low specificity) and that a wide range of emotion words was used to label clusters associated with the same pattern (low consistency). Moreover, the words associated with a given pattern varied in terms of both normative valence (Fig. 4, left panel) and arousal (Fig. 4, right panel). The most frequent pattern (Pattern 1), for example, is associated with “happy”, “calm”, “annoyed”, and “bored”. These findings replicate and extend the within-participant analysis of emotion words, and illustrate the scale of variation in affective experience across participants, even when the patterns of change in physiological activity for those experiences are largely similar.

We assessed all identified clusters according to whether they represented changes in each branch of the ANS, using RSA change scores as an estimate of PNS function^57^, and PEP change scores as an inverse estimate of SNS function^53,55,58^ (though it should be noted that RSA is a chronotropic measure and PEP is an inotropic measure of cardiac activity). Changes in PNS function, whether withdrawal or increased activation, were present in about 74% of clusters (withdrawal: 45.21%; activation: 29.38%), whereas changes in SNS function were present in about 55% of clusters (withdrawal: 20.55%; activation: 34.25%). A chi-square test of independence confirmed that the proportion of changes was significantly different between the two branches, *X*^2^(2) = 33.54, *p* < 0.001, suggesting that changes in PNS activity may be a larger contributor to cardiovascular function in daily life. These findings are consistent with prior research on the autonomic space model of cardiovascular function, which found that changes in PNS function alter heart rate over a much larger dynamic range than do changes in SNS function^59^. The larger proportion of PNS changes also might be explained by the fact that all events occurred in the absence of recent gross movement or posture change, and the majority of events in each cluster occurred when participants were sitting (73.88%) versus standing (12.47%) or reclining (8.27%). Postural changes strongly influence autonomic activity^53^, and SNS contributions to changes in heart rate are greater during physical activity^60,61^.

**Figure 5:**
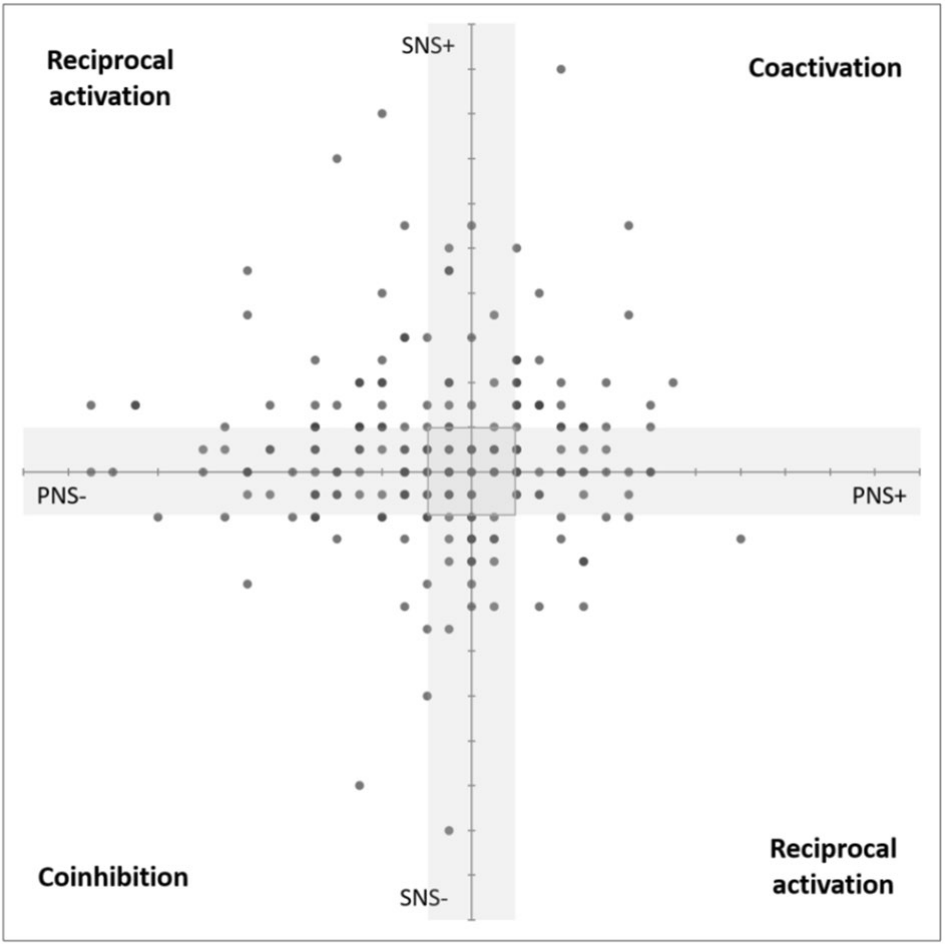
Modes of autonomic cardiac control for all 219 identified clusters. Clusters are plotted according to within-person effect size estimates for changes in PNS activity (x-axis) and SNS activity (y-axis). Quadrants are labeled following established standards^59^: a ‘reciprocal’ mode occurs when activity in the PNS and SNS are changing in opposite directions (i.e., one withdrawal, the other activation); a ‘coactivational’ mode occurs when PNS and SNS activity is changing in the same direction (i.e., both activation or both withdrawal, the latter also referred to as ‘coinhibition’). An ‘uncoupled’ mode occurs when activity in only one branch is changing substantially and is represented by the gray shading around the axes. ‘Negligible change’ occurs when neither branch shows a change in function, and is represented by the shaded square around the origin.

Figure 5 plots clusters according to changes in PNS and SNS activity, to illustrate the potential mode of autonomic cardiac control represented by each^59^. Across all identified clusters, 50.23% represented an uncoupled control mode, such that there was a change in activity of only one branch of the ANS. ANS modes that were reciprocal (i.e., activity in one branch increased while activity in the other decreased) or coactivational (i.e., activity in both branches increased or decreased) occurred in 19.63% and 20.09% of the clusters, respectively. Negligible change in the activity of both branches accounted for 10.05% of clusters across participants (note that, because these classifications were determined using within-person effect size estimates, ‘negligible change’ merely indicates that changes in PNS or SNS activity were small relative to other changes for that participant). This distribution of clusters across different modes of ANS control is particularly interesting in light of the pervasive assumption that the PNS and SNS are inherently reciprocally organized and functionally antagonistic^59^. The occurrence of equivalent proportions of clusters associated with coactivational and reciprocal modes may be reflective of the high ecological validity of the present study and its ability to capture changes in everyday life.

## Discussion

Many of the ongoing debates about emotion center on questions about the amount of variation in the physical and mental features of emotional events, as well as the nature of that variation. To date, studies have typically been designed to observe consistency and specificity in physiological activity across instances of an emotion category—yet have still observed more variation than expected^10,23,28–36^. By using a novel biologically-triggered experience sampling method, the present study builds on a growing body of work to show that empirical approach matters: when studies are designed to observe variation, they find heterogeneity within emotion categories, in addition to similarity across emotion categories^23,40,62^. Here, participants reported affective experience while their peripheral physiological activity was recorded, specifically during instances of substantial change in cardiac activity in the absence of movement. This method enhanced the likelihood of sampling instances having greater psychological salience, and thus enhanced the chances of observing heterogeneity in emotional experience. Unsupervised clustering analyses revealed variation in the number and nature of patterns of physiological activity that recurred within individuals over time, as well as heterogeneity in the affect ratings and emotion labels associated with each pattern. These findings suggest that physiological and mental features are non-redundant measures of emotional episodes, and may help explain the persistent difficulty in observing robust, stable correlations between self-reports and physiological measures^63^. Further, the degree of variation that we observed is inconsistent with classical and prototype views of emotion^1,3,7^. We did not find evidence to support a fingerprints hypothesis. No emotion-specific diagnostic patterns of physiology emerged. Rather, patterns of physiological activity varied in context-dependent ways. These findings support a populations hypothesis positing structure within variation, both within and across participants^10,17^.

Although a number of theoretical views on emotion are more or less consistent with a populations hypothesis^14–20^, it has been most explicitly developed within the theory of constructed emotion^17,18^. The theory of constructed emotion begins with the observation that the primary purpose of the brain is to predictively regulate physiological resources to most efficiently manage the brain’s and body’s energy needs—a process referred to as allostasis^64,65^. To do this, the brain uses prior experience to build and maintain an internal model of the world that includes its own body^66–69^. In a given instance, the brain is hypothesized to construct, ad hoc, a population of temporally dynamic representations that are similar to the current sensory array. Relying on similarity (as opposed to exact matching) is functional, because the representations can vary in their physical and mental features across instances. For example, different patterns of physiological activity are required to support shouting and flailing ones arms versus silently brooding in anger, and only one of these is likely to be functional within a particular anger-relevant context (e.g., being reprimanded by a supervisor). These ad-hoc, in-the-moment representations function as prediction signals that begin as visceromotor^66–69^ and motor control signals^70,71^. Efferent copies of these signals give rise to the predicted sensations that will result from those movements as well as hypotheses about the causes of those sensations^17,18^. In turn, this entire cascade of sensory and (viscero)motor detail is compressed into a low-dimensional, multi-modal abstraction, which the brain can use as needed to issue future situation-specific predictions. As this abstraction process operates over time, a wide range of highly variable affective and physical experiences become functionally associated with each other, forming a population of instances within the same emotion category (e.g., anger). Even within a single individual, these instances will vary considerably in their physiological features because they are coupled to the energetic needs of each specific situation. In this way, variation in patterns of physiological activity across emotion instances is inherently meaningful. Further, the theory of constructed emotion posits that a particular pattern of physiological activity can be associated with mental features that vary substantially (e.g., valence and arousal, evaluations of novelty or controllability), such that these features are neither consistent with nor specific to any particular emotion category.

Critically, the variability predicted by the theory of constructed emotion is thought to be situated, not random^15–18^. This is because peripheral physiological changes are yoked to the energy needs of current or anticipated action^64,72–74^. For example, actions such as freezing and laughing in fear may each be supported by a distinct pattern of physiological activity (resulting in within-category and within-participant variation), whereas smiling in anger and happiness, for example, may share more physiological features (resulting in across-category and across-participant similarity). In the present study, we observed across many participants that a larger proportion of experience sampling events were associated with smaller physiological changes. We also observed some consistency in patterns of change in physiological activity across individuals. These observations suggest that more consistent patterns can occur, but that revealing the sources of these consistencies will require future research to assess a number of situational features, including social and motivational context. Such factors have been shown to influence physiological activity, even for two experiences labeled with the same emotion word^75,76^. Likewise, future research should further explore how physical bodily contexts, such as activity and posture, play a role in constructing affective experience in everyday life^77–79^.

To more fully understand the situation-specific construction of affective experience, scientists must conduct research that extends beyond the narrow confines of the psychology laboratory to sample a broad range of human activities and experiences^39,80^. In the present study, we used physiologically-triggered experience sampling that allowed us to observe a much broader range of ecologically-valid affective experiences and their accompanying physiological activity. Using these data, we identified patterns of change in physiological activity that represented multiple modes of autonomic control (reciprocal, coactivation, coinhibition, and uncoupled modes^58,59^), illustrating the utility of this method for documenting patterns of physiological activity that may be less commonly observed in a controlled laboratory setting. This proof-of-concept study can be expanded in future research to include other measured features, such as more complex motion sensing, self-reported bodily sensations, geolocation data, or additional ambulatory physiological measures. In doing so, future research should also undertake time-dynamic analyses that can account for diurnal trends in physiological activity.

The present study also utilized innovative analytical methods for detecting patterns of change in physiological activity and their association with mental features of affective experience. To date, most studies have used supervised machine learning (i.e., classification) algorithms to deductively assess the relationship between patterns of physiological activity and categories of emotional experience^81–83^. Those using these classification-based approaches often interpret the patterns they identify for each emotion category as a fingerprint of that category^81,84–86^, rather than as the statistical summary that such patterns actually represent^36^. In contrast, we employed person-specific unsupervised machine learning (i.e., clustering) algorithms to discover the number and nature of patterns of change in physiological activity, and then inductively examined the relationships between these patterns and emotional experience. Our group has recently used similar approaches to understand the relationships between change in physiological activity and subjective experience in the more constrained context of a motivated performance laboratory task to assess physiological patterns of challenge or threat^48,87^. The current work demonstrates that this method not only can be used on data collected outside the laboratory, but affords an inductive, data-driven approach that enables the discovery of variability, rather than stipulating experimenter-labelled categories as ground truth.

As with any study, there are limitations and constraints on generalization. One limitation is that we triggered experience sampling prompts solely on the basis of IBI change, which itself can be constrained, particularly during coactivation or coinhibition modes of ANS control^59^. As a result, we may have excluded from sampling some moments of significant psychological salience or of change in physiological features other than IBI. Notwithstanding, the approach here provides a better chance of sampling experience at times salient to the individual than does traditional random sampling. Likewise, by sampling only in the absence of movement, we potentially reduced the number of evocative emotional events available for study. Although this decision was necessary to prevent triggering experience sampling prompts that could have been solely due to movement, future research should consider means of addressing this limitation. For example, future studies could utilize separate triggers for movement- and non-movement-related prompts and assess which, if any, movement-related prompts are also psychologically salient. The homogeneity of our sample and sampling conditions can also be considered a constraint on generalization: most of our participants were university students, whose physiology and self-reports were recorded during weekdays, often while they were in class or working at a desk. At the same time, this limitation gives the advantage to a fingerprints hypothesis, as more constrained sampling should result, if anything, in greater consistency. Further, the relatively small sample size makes it difficult to perform robust between-participants analyses, which are needed to more fully assess the extent of similarities and differences across individuals and to assess the role of theoretically and practically meaningful individual differences (e.g., age, sex, emotional granularity, interoceptive sensitivity) in explaining variability across individuals.

Despite these limitations, however, the current approach opens the empirical door for innovations in emotion research. For over a hundred years, scientists have induced instances of emotion and measured the changes in physiological activity to those inductions^88^ in an attempt to identify the defining biological features of emotion categories. With the empirical approach adopted in the present study, we are finally heeding the advice of William James, who wrote “there is no limit to the number of possible different emotions which may exist, and why the emotions of different individuals may vary indefinitely, both as to their constitution and as to objects which call them forth” (p. 454)^89^. We now have the requisite methodological and analytical tools to better understand the individual- and situation-specific physiological and mental constituents of affective experience in everyday life.

## Methods

All experimental protocols described below were approved by the Northeastern University Institutional Review Board. These methods were carried out in accordance with the relevant guidelines and regulations for research with human subjects.

### Participants

Sixty-seven participants ranging in age from 18–36 years (55% female; 38.8% White, 3.0% Black, 29.8% Asian, 28.4% other; *M*_*age*_ = 22.8 years, *SD*_*age*_ = 4.4 years) were recruited from the greater Boston area through posted advertisements, as well as Northeastern University classrooms and online portals. Eligible participants were non-smoking, fluent English-speakers, and excluded if they had a history of cardiovascular illness or stroke, chronic medical conditions, mental illness, asthma, skin allergies, or very sensitive skin. Eligible participants also confirmed they were not taking any medications known to influence physiological arousal including medications for ADHD, insomnia, anxiety, hypertension, rheumatoid arthritis, epilepsy/seizures, cold/flu, or fever/allergies. Informed consent was obtained from all participants before beginning the study. Participants received $490 as compensation for completing all parts of the study, plus up to an additional $55 in compliance and task incentives, as detailed on page 4 of the supplementary information.

Of the 67 recruited participants, six withdrew and an additional nine were dismissed due to poor compliance. A total of 52 participants completed the full protocol (56% female; 38.5% White, 1.9% Black, 38.5% Asian, 21.2% other; *M*_*age*_ = 22.5 years, *SD*_*age*_ = 4.4 years). Six of these participants were excluded from the main analyses due to a lack of sufficient usable physiological data, bringing the final sample size to 46 (50% female; 43.5% White, 0% Black, 41.3% Asian, 15.2% other; *M*_*age*_ = 22.4 years, *SD*_*age*_ = 4.4 years).

### Procedure Overview

Each participant completed approximately 14 days (*M* = 14.4, *SD* = 0.6) of context-aware experience sampling distributed across a three- to four-week period (*M* = 24.9 days, *SD* = 5.5 days). On each day of experience sampling, participants came into the lab and were instrumented for peripheral physiological recordings. Participants could not begin the experience sampling portion of the study without functioning ambulatory equipment, and so did not complete the daily protocol if they did not attend the morning session to be instrumented for the recordings. In the event a participant was unable to make a scheduled session or the equipment was not functioning properly during instrumentation, they were rescheduled to complete the day of sampling on another day. Participants generally started each experience sampling day between 8 and 9 am, although this varied between 7:30am and 2:30 pm according to participants’ schedules. Participants were provided an Android smartphone with a custom application, MESA (MindWare Technologies LTD, Westerville, OH), which was used to determine when a heart rate response of sufficient duration and amplitude occurred which then triggered an experience sampling prompt. Participants were instructed to continue physiological recordings for eight hours each day, after which they were able to remove and recharge all equipment. Upon completing experience sampling each day, participants automatically received an end-of-day survey via SurveyMonkey (San Mateo, CA), which they used to provide additional details about the prompts they completed throughout the day. Before and after the two-week experience sampling protocol, participants also completed two in-lab sessions. In each session, participants completed tasks and questionnaires that are not reported here (see page 5 of supplementary information and supplementary Table S1 for an overview).

#### Physiological measurement

All ambulatory peripheral physiological measures were recorded at 500 Hz on a mobile impedance cardiograph from MindWare Technologies LTD (Model # 50-2,303-02, Westerville, OH), which participants wore clipped onto their clothing on the hip.

ECG and ICG were obtained using pre-gelled ConMed (Westborough, MA) Cleartrace Ag/AgCl sensors, connected via wires to the cardiograph. Sensor sites were cleaned with alcohol and abraded lightly with gauze. ECG was obtained using a modified lead II configuration, with recording electrodes placed on the distal right collarbone and an inferior left rib, respectively, and a reference electrode placed on an inferior right rib. The ECG signal was acquired using a low cutoff of 0.5 Hz and a high cutoff of 45 Hz.

ICG was obtained using a four-spot electrode configuration^90^. Two inner recording electrodes were placed on the front of the torso: one at the base of the neck at the top of the sternum, and a second at the bottom of the sternum over the xiphisternal junction. Two outer source electrodes were placed on the back along the midline approximately 4 cm above and below the inner recording electrodes, respectively. The source electrodes passed a 4 mA, 100 kHz alternating current across the thorax. The distance between the inner recording electrodes (cm) was measured for each day of experience sampling. Basal impedance (Z_0_) was acquired using a low cutoff of 10 Hz. The first derivative, dZ/dt, was acquired using a low cutoff of 0.5 Hz and a high cutoff of 45 Hz.

Electrodermal activity (EDA) was also recorded over an unobtrusive neck placement, but data were not used in the present analysis due to difficulties in detecting and removing artifacts, as well as generally low mean skin conductance levels across participants.

The mobile impedance cardiograph collected continuous three-axis accelerometry data used to assess movement. Additionally, participants wore two inertial measurement units (IMUs) purchased from LP-Research (Minato-ky, Tokyo, Japan) to derive measures of posture and changes in posture. One IMU was placed medially on the sternum beneath the top inner impedance recording electrode and affixed to the skin using a double-sided adhesive patch. The other IMU was placed on the front of the thigh using either a cloth holder attached to the clothing, or a second adhesive patch affixed to the skin. Participants did not remove sensors until the end of each experience sampling day, unless instructed by the experimenters (e.g., due to synchronizing issues).

#### Context-aware experience sampling

Peripheral physiological data and accelerometric data were recorded continuously throughout the 8-h sampling period and communicated via Bluetooth to a Motorola Moto G^4^ smartphone. The smartphone application, MESA, processed the continuous ECG and accelerometer data in real time, and initiated an experience sampling prompt anytime a substantial, sustained change in heart period was detected in the absence of movement or posture change, with an imposed minimum interval of five minutes between prompts. A substantial, sustained change in heart period was operationalized on the first day of sampling as occurring when the interbeat interval (IBI) changed by more than ± 167 ms over an eight-second period (at a typical resting heart rate of 60 bpm or IBI of 1,000 ms, this is equivalent to a decrease of about 9 bpm or an increase of about 12 bpm). On subsequent days, this IBI parameter was manually adjusted up or down to ensure each participant received approximately 20 prompts per day. The average adjustment was ± 26.17 ms (*SD* = 25.91 ms), with the final parameter setting ranging between ± 65 ms and ± 266 ms across participants. For background on threshold selection and more information on threshold adjustment, see page 4 of the supplementary information.

Movement was determined from the continuous accelerometer data from the mobile impedance cardiograph. Minimal movement was operationalized as any time none of the three accelerometry channels (alone or in aggregate) exceeded a threshold of 10 cm/s^2^ within the preceding 30 s. Posture (standing, sitting, reclining) was determined by comparing the relative orientation of the two IMUs on participants’ torso and thigh using their continuous accelerometry data. Absence of posture change was operationalized as any time when the relative orientation of the two IMUs did not change within the preceding 30 s.

On average, participants received 21.70 (*SD* = 6.90) prompts per day. We observed that prompts were relatively evenly distributed throughout the day within experience sampling days. On average, participants completed 34% of prompts in the morning (before 12 pm), 52% of prompts in the afternoon (12–5 pm), and 14% of prompts in the evening (after 5 pm). This makes sense given the typical start times for participants in our sample. Participants also received on average two ‘random’ prompts per experience sampling day, which occurred in the absence of movement or posture change, but which were not contingent on a change in IBI. Random prompts were spread throughout the experience sampling day, such that they could only receive one in the first four hours and one in the second four hours of experience sampling. Participants were informed that some experience sampling prompts would be generated randomly while others would be generated based on changes in their cardiac activity. By conveying this information, we were able to instruct participants to avoid responding to prompts following specific physiological events (e.g., coughing, sneezing) and minimize the extent to which they paid special attention to their cardiac activity.

To remain in the study, participants were required to complete at least three prompts each day. In addition, as detailed on page 4 of the supplementary information, for the purposes of incentivizing participation and limiting attrition, the experience sampling protocol was broken into three pay periods (days 1–5, 6–10, and 11–14). Participants were required to respond to an average of at least six prompts per day during each pay period to remain in the study, and received a bonus payment for each pay period where they completed an average of eight prompts per day. The average number of completed prompts was 8.80 per day (*SD* = 1.22), which is in line with previous experience sampling studies that have asked participants to complete 10 prompts per day^91,92^.

At each sampling event, participants were prompted to respond to a series of questions presented in the MESA application. Participants first provided a brief free-text description of what was happening at the time they received the prompt. Next, participants rated their current valence and arousal, each on a 100-point continuous slider scale ranging from -50 (very unpleasant or very deactivated) to + 50 (very pleasant or very activated). Participants then self-generated words to label their current affective experience. Specifically, participants were asked to “list any emotion(s) you were feeling when you received the prompt”. Participants were able to provide as many words as they felt necessary to describe their affective experience but were required to input at least one word. For each self-generated word, participants were asked to provide an intensity rating on a five-point scale: “not at all” (1), “a little” (2), “moderately” (3), “a lot” (4), “very much” (5). We asked participants to self-generate emotion words as this allowed us to capture naturally-occurring variation in how participants categorized their affective experience, as opposed to having them select words from an experimenter-stipulated list, which would necessarily constrain variation. Participants also responded to additional questions that are not included in the present report (see page 4 of the supplementary information for details).

At the end of each experience sampling day, participants received a modified day reconstruction survey^93^, in which they were presented with their self-generated brief description and social context from each prompt they completed during the day. For each prompt, participants were asked to provide additional details about the event, its social context, and accompanying affective features. Participants were requested to select three sampling events for which they provided a longer, more detailed description (> 200 words). Full details of the end-of-day survey are provided on pages 5–6 of the supplementary information. Data from these surveys are not reported here.

#### Physiological signal processing and feature extraction

Peripheral physiological signals were processed using an in-house pipeline coded in Python to accommodate the volume of physiological data collected (400 + hours), as well as the variability in signal morphologies and artifacts produced during long-term ambulatory monitoring. Artifacts were identified using a series of quality checks, detailed for each signal below. Data affected by artifacts were excluded from analysis (i.e., artifacts were not corrected). This resulted in an average event-related data loss of 8.18% per day (*SD* = 3.35%) across ECG (5.79% average data loss; *SD* = 3.22%) and ICG (2.56% average daily data loss; *SD* = 2.03%) signals. The research team, including two trained psychophysiologists (J.B.W., K.S.Q.), met regularly throughout development of the processing pipeline to visually examine sample output and provide feedback on quality checks.

#### Electrocardiogram (ECG)

The ECG signal was processed following previous work^94^. First, the raw signal was passed through an elliptic bandpass filter to remove baseline and high frequency noise without affecting the waveform. Initial quality checks were then performed for each beat, checking for overall waveform shape, and acceptable minimum, maximum, and minimum-to-maximum values^94^. The default parameters, originally selected based on data collected in laboratory settings, were found to be too aggressive for this ambulatory data set, so we relaxed these thresholds based on manual review of the results by experts (J.B.W., K.S.Q.) for a random subset of the data. The parameters we implemented are reported in supplementary Table S2, along with the original recommendations^94^.

R-peak detection for ECG was performed using established methods^95^ and implemented using the BioSPPy package in Python^96^. Interbeat interval (IBI) was then derived as the average R-R interval. Additional quality checks (supplementary Table S2) were performed on each IBI series to ensure that values were within acceptable ranges (300–2000 ms), and that expected beat-to-beat differences were consistent with normal beats and unlikely to be artifacts (following established benchmarks^97^). ECG data failing any quality check were excluded from analysis.

Respiratory sinus arrhythmia (RSA) was derived from the IBI series. RSA reflects high-frequency variability in IBI which occurs at the respiratory frequency and is often used as an estimate of parasympathetic influence on the heart^57^. RSA calculations were coded to mimic the processing steps of standard heart rate variability (HRV) analysis software (MindWare Technologies LTD, Westerville, Ohio), including: cubic interpolation of beat-to-beat IBI, detrending to minimize non-stationarity, tapering using a Hamming window, and lastly, fast Fourier transformation (FFT). RSA was calculated as the log of the area under the power spectrogram that lies between 0.12 and 0.4 Hz.

#### Impedance cardiogram (ICG)

ICG feature detection was performed using an in-house implementation based on methods described in previous work^94,98^, and adjusted for ambulatory data using thresholds determined by comparing performance of the original algorithms with hand-scored impedance cardiographic data on a subset of participants. The first derivative of the basal impedance (Z_0_) signal, dZ/dt, was used as the basis for all analyses. The signal was segmented into time windows corresponding to 250 ms before the ECG R-peak to 500 ms after; eight such segments (i.e., 8 beats) were averaged together to form overlapping ensembles^94^. B points were detected in each ensemble by taking the first and second derivatives of the dZ/dt signal and comparing them with thresholds based on signal frequency (supplementary Table S2)^98^. Following standard procedures^98^, forward and reverse autoregressive modeling was then used to perform outlier detection and correction, such that all B points in a series were fit with the autoregressive model. X points were detected by examining the second derivative of the dZ/dt signal within each ensemble^94^. Segments of the ICG signal from which we could not detect B or X points and ICG data that corresponded to periods of unusable ECG data were excluded from analysis.

Two features we used were systolic time intervals, namely, pre-ejection period (PEP) and left ventricular ejection time (LVET). PEP represents the time interval in ms between the electrical event that signals the start of ventricular contraction to the opening of the aortic valve. PEP is often used as an inverse estimate of sympathetic control of the heart^53,55,58^. Here, PEP was calculated as the time in ms between the ECG R peak and the ICG B point (also referred to as PEP_R_^99^). LVET is the time interval in ms between the opening and closing of the aortic valve^55^, and was calculated as the time in ms between the ICG B point and X point. Quality checks (supplementary Table S2) were performed and we retained only values that occurred within an acceptable range (30–200 ms for PEP, 100–500 ms for LVET), and that did not result in changes in the gradient greater than 30 ms from one ensemble to the next.

Two additional features we used were based on volumetric measures of cardiac function, namely, stroke volume (SV) and cardiac output (CO). SV represents the volume of blood ejected by the heart with each heartbeat (in mL), and here we calculated SV using Kubicek’s equation^100^. CO is the volume of blood circulated by the heart in L/min^55^.

### Analysis

#### Within-person clustering analysis

We submitted data from each individual to a separate clustering analysis using Dirichlet Process-Gaussian Mixture Modeling (DP-GMM)^49, 50^. DP-GMM is a specialized variant of Gaussian Mixture Modeling (GMM)^49^. Data points (i.e., six-dimensional vectors of change scores for IBI, RSA, PEP, LVET, SV, and CO) were standardized prior to clustering by subtracting the mean and dividing by the standard deviation of each feature. Specific parameter values are reported in supplementary Table S3.

The first step in implementing standard GMM is to initialize a set of parameters for a predefined number of clusters represented in the data. Parameters (i.e., values) include each cluster’s location (i.e., mean), shape (i.e., covariance), and relative size (i.e., the mixture proportion or the prior probability of a point belonging to that cluster relative to others). Initially, these cluster parameters are chosen at random, with means and covariances selected from a Gaussian distribution that is proportional to the data, and with prior probabilities of equal value. GMM uses these initialized values to calculate the posterior probabilities of each point belonging to each cluster. Under mixture models, each point is given a posterior probability of belonging to *every* cluster; however, if patterns in the data are distinctive, posterior probabilities during later iterations will often be close to 1 for a single cluster and negligible for all others. Based on the posterior probabilities, the means and covariance matrices are updated iteratively using optimization techniques such as Expectation Maximization^49^ or Variational Inference^49^ until reaching convergence (i.e., until the values stop changing).

In the present analyses, we sought to avoid tuning and selecting the number of clusters for each participant. DP-GMM allowed us to accomplish this goal as, in contrast to standard GMM, DP-GMM *discovers* the number of clusters from input data through the use of a Dirichlet Process Prior^101^. The Dirichlet Process Prior enables a dataset to include an unknown (i.e., infinite) number of clusters, where the mixture proportion of each cluster is randomly assigned a value from a continuous probability distribution such that the mixture proportions must sum to 1. This random assignment process is repeated ad infinitum to create a vector of mixing proportions which then serves as the prior probabilities of a standard GMM. To place reasonable bounds on this process, the number of clusters made discoverable through Variational Inference was limited to the number of data points in the sample. Because DP-GMM iteratively optimizes parameters (means, covariances, and mixture proportions), clusters which initially acquire more points become more likely to continue acquiring points in the future. Consequently, as the model runs, some clusters grow while others shrink and are ultimately discarded.

As a fully Bayesian method, DP-GMM is more likely to avoid overfitting because it is self-regularized by the priors^101^. There are, however, model parameters that can constrain the number of clusters discovered. As detailed on page 28 of the supplementary information, we ran additional analyses to validate the values selected for these parameters. We also conducted parallel analyses on the example participants using a standard GMM approach, in which Bayesian information criterion (BIC) values were used to select the optimal number of clusters per participant. These analyses replicated our main findings with regard to the heterogeneity of clusters discovered and their relationship with mental features of affective experience (see pages 29–30 of the supplementary information for details).

#### Effect size estimates

We derived effect size estimates (similar to Cohen’s *d*^56^) for the mean change scores for IBI, RSA, PEP, LVET, SV, and CO for each cluster for each participant using the following formula:

$$d= \frac{m- \mu }{\sigma }$$

where *m* is the weighted cluster mean for a given physiological feature, µ is the assumed population mean corresponding to no change (i.e., 0), and *σ* is the standard deviation for the given physiological feature for that participant across all events included in the analysis. According to published recommendations for interpreting effect sizes^54^, we interpreted the magnitude of the weighted mean change score for each feature for each cluster as: |*d*|< 0.2 (negligible change); 0.2 ≤|*d*|< 0.5 (small change); 0.5 ≤|*d*|< 0.8 (medium change); |*d*|≥ 0.8 (large change).

#### Common patterns of change in physiological activity

We classified change score effect sizes as a negligible change (|*d*|< 0.2), decrease (*d* ≤ −0.2), or increase (*d* ≥ 0.2). Before classification, we rounded the effect size estimates to the nearest tenth (i.e., one decimal place) to facilitate comparison with published recommendations. Logical syntax was used to identify duplicate (i.e., repeated) strings of effect size classifications.

We performed further classifications of RSA and PEP change scores to assess overall trends in the change in PNS and SNS function, respectively. Larger change score effect sizes for RSA (i.e., greater variability in interbeat interval within the respiratory frequency band) indicate increased PNS function, such that: |*d*|< 0.2 (negligible PNS change); *d* ≥ 0.2 (PNS activation); and *d* ≤ −0.2 (PNS withdrawal). For PEP, larger change score effect sizes (i.e., longer pre-ejection periods) indicate SNS withdrawal, such that: |*d*|< 0.2 (negligible SNS change); *d* ≥ 0.2 (SNS withdrawal); and *d* ≤ −0.2 (SNS activation).

## Supplementary information

Supplementary file1 (DOCX 6045 kb)
