# Author Correction: Context-aware experience sampling reveals the scale of variation in affective experience

**DOI:** 10.1038/s41598-021-82415-w

**Published:** 2021-01-26

**Authors:** Katie Hoemann, Zulqarnain Khan, Mallory J. Feldman, Catie Nielson, Madeleine Devlin, Jennifer Dy, Lisa Feldman Barrett, Jolie B. Wormwood, Karen S. Quigley

**Affiliations:** 1 Northeastern University, Boston, USA; 2 University of North Carolina at Chapel Hill, Chapel Hill, USA; 3 Martinos Center for Biomedical Imaging, Massachusetts General Hospital, Charlestown, USA; 4 University of New Hampshire, Durham, USA; 5 Edith Nourse Rogers Memorial Veterans Hospital, Bedford, USA

Correction to: *Scientific Reports* 10.1038/s41598-020-69180-y, published online 27 July 2020

The Supplementary file1 that accompanies this Article contains an error in Supplementary Figure S3, where the error bars for “Posture” and “Time of Day” are reversed. The correct Figure S3 appears below as Figure 1.

**Figure 1:**
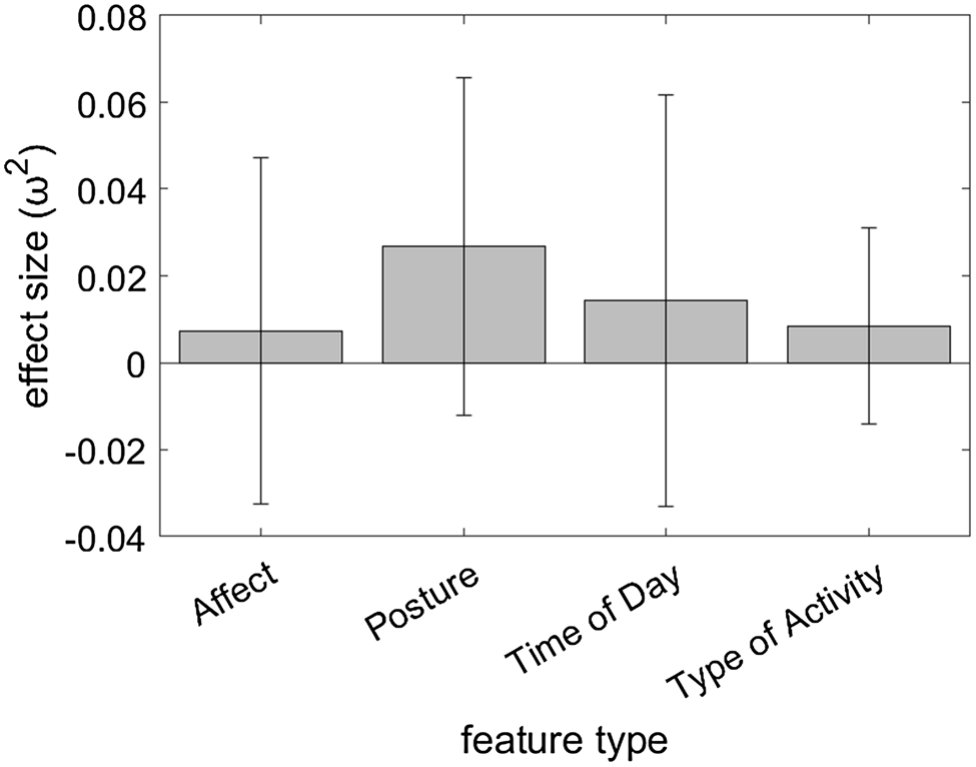
Comparison of effect size (*ω*^2^) by feature type in participant-level comparisons across clusters. Error bars represent standard deviations.

Additionally, the Acknowledgements section in this Article was omitted. The Acknowledgements section should read:

“This work was supported by Army Research Institute grant W911NF-16-1-0191 (PIs: K.S.Q. & J.B.W.). The views, opinions, and/or findings contained in this report (paper) are those of the authors and shall not be construed as an official Department of the Army position, policy, or decision, unless so designated by other documents. K.H. was further supported by the National Heart, Lung, and Blood Institute (grant number 1F31HL140943-01).”

