# Supplementary material for: Context-aware experience sampling reveals the scale of variation in affective experience
Source: Sci Rep. 2020 Jul 27;10:12459. doi: 10.1038/s41598-020-69180-y (PMC7385108; doi:10.1038/s41598-020-69180-y)
Supplement: Supplementary file 1 — Supplementary file1 (DOCX 6045 kb) [file 41598_2020_69180_MOESM1_ESM.docx]

Supplementary information for: “Context-Aware Experience Sampling Reveals the Scale of Variation in Affective Experience”

Katie Hoemann^1*^, Zulqarnain Khan^1*^, Mallory J. Feldman^2^, Catie Nielson^1^, Madeleine Devlin^1^, Jennifer Dy^1^, Lisa Feldman Barrett^1,3^, Jolie B. Wormwood^4,5†^, Karen S. Quigley^1,5†^

1. Northeastern University
2. University of North Carolina at Chapel Hill
3. Massachusetts General Hospital/Martinos Center for Biomedical Imaging
4. University of New Hampshire
5. Edith Nourse Rogers Memorial Veterans Hospital

* Indicates shared first authorship

† Indicates shared senior authorship

**Overview**

This appendix contains information on the following topics:

1. Supplementary Background
   1. Explanatory frameworks in emotion science (Figure S1) (p. 3)
2. Supplementary Methods
   1. Example experience sampling event (Figure S2) (p. 3)
   2. IBI change threshold and adjustment (p. 4)
   3. Experience sampling questions (p. 4)
   4. Payment (p. 4)
   5. In-lab tasks and questionnaires (p. 5)
   6. End-of-day survey (p. 5-6)
   7. Questionnaire measures (Table S1) (p. 7)
   8. Physiological signal processing (Table S2) (p. 8)
   9. Unsupervised cluster analysis (Table S3) (p. 8)
3. Supplementary Results
   1. Participant-level results from within-person clustering analysis (Table S4) (pp. 9-10)
   2. Cluster-level results from within-person clustering analysis (Table S5) (pp. 11-17)
   3. Participant-level comparisons across clusters (p. 18)
   4. Summary of participant-level Kruskal-Wallis tests (Table S6) (pp. 19-20)
   5. All repeated patterns of change in physiological activity (Table S7) (p. 21)
   6. Bubble plots of unique words freely generated to label experience sampling events (Figure S4) (pp. 22-27)
   7. Validation of model parameters (p. 28)
   8. Comparison against standard Gaussian Mixture Modeling (pp. 29-30)
4. References (pp. 31-32)

**Supplementary Background**


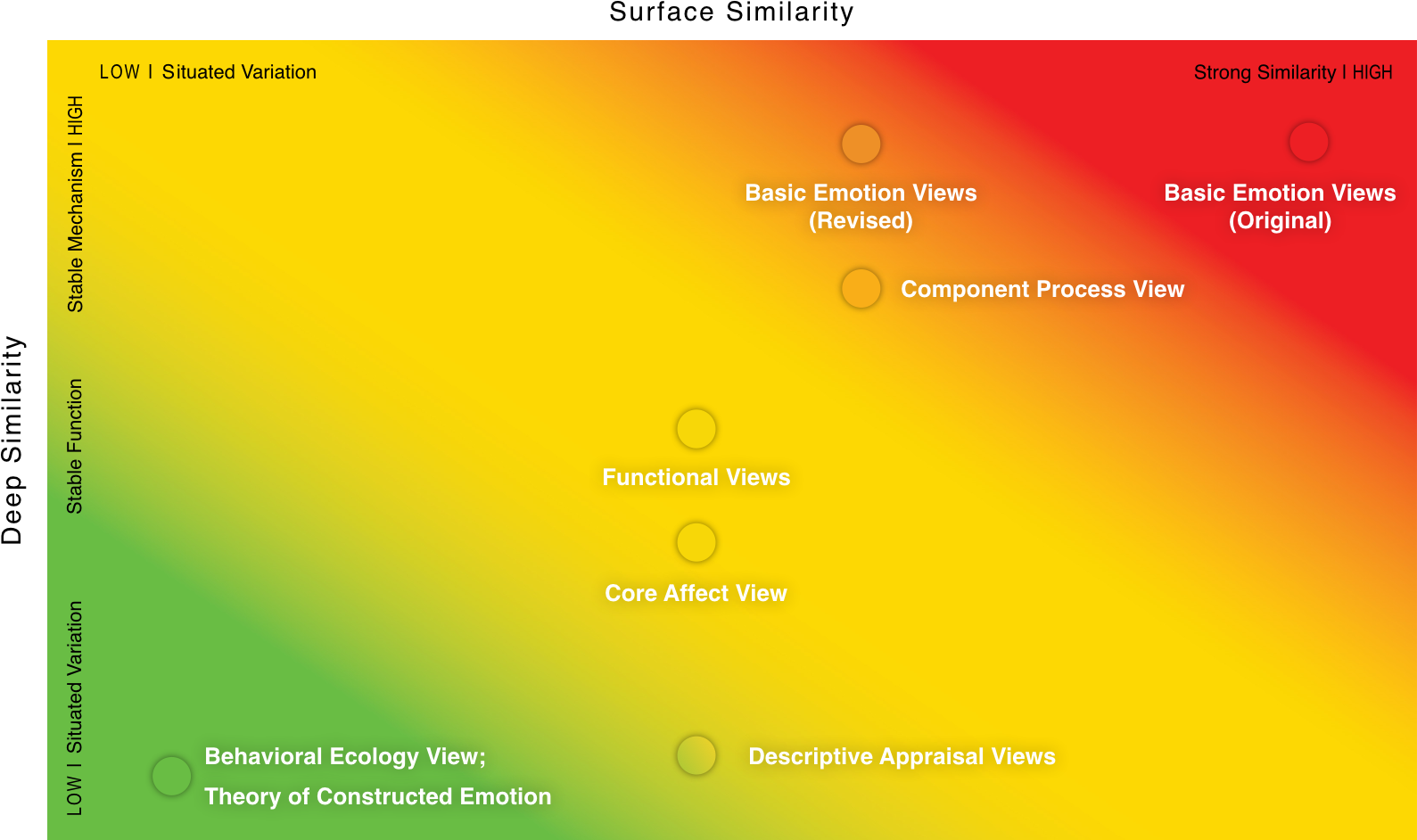


**Figure S1.** Explanatory frameworks in emotion science. Theoretical approaches hypothesize the nature of emotion categories. Surface similarity: hypotheses about the degree to which instances of an emotion category vary in their observable features. Deep similarity: hypotheses about the similarities in the mechanisms that cause instances of the same emotion category (e.g., the neural circuits or assemblies that cause instances of the same emotion category). The colors represent the type of emotion categories proposed: ad hoc, abstract categories (green zone); prototype or theory-based categories (yellow zone); classical or natural-kind categories (red zone). Adapted with permission from Barrett et al (2019), *Psychological Science in the Public Interest*^1^.

**Supplementary Methods**


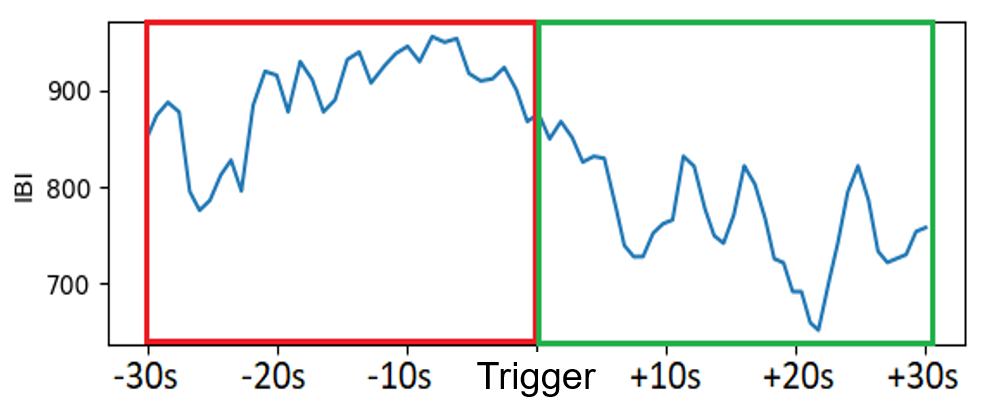


**Figure S2.** Example interbeat interval (IBI) series taken from ECG signal 30 seconds preceding and following an event trigger, or the start of the period of heart rate change preceding an experience sampling prompt. All physiological measures are calculated as change scores, in which the mean of the 30 seconds preceding the trigger is subtracted from the mean of the 30 seconds following the trigger.

**IBI Change Threshold and Adjustment**

To assess the feasibility of our biologically-triggered sampling approach, we completed a pilot project with five individuals. We found that an increase or decrease in heart rate of 10 beats per minute or more sustained over at least 8 seconds was reasonable for initiating a measurement moment during experience sampling. The pilot study showed that these heart rate parameters resulted in a sufficient number of prompts per day (a minimum of 2.5 prompts/hour for five individuals, each of whom were sampled for 2.0-6.5 hours). We used ±167 ms for our study because it corresponds to a heart rate change of ~10 bpm at an average heart rate (~60-65 bpm).

All participants started experience sampling with the standard threshold of ±167ms. If participants needed more prompts during the day, we lowered the threshold in 17ms increments (e.g., from ±167 ms to ±150 ms to ±133 ms). This increment was also established based on pilot testing. If participants needed fewer prompts during the day, we used a data-driven approach to increase the threshold: we examined the participant’s previous days’ data to determine what IBI changes were common, and adjusted the threshold so that the participant would receive approx. 20 prompts/day. The average change was ±26.17 ms (*SD* = 25.91 ms).

**Experience Sampling Questions**

At each sampling event, participants were prompted to respond to a series of questions presented in the MESA application. First, participants provided a brief free-text description of what was going on at the time they received the prompt. Second, participants rated their current valence and arousal, each on a 100-point continuous slider scale ranging from -50 (very unpleasant or deactivated) to +50 (very pleasant or activated). Third, participants provided another brief free-text description of their social context by: writing “alone”, listing the initials of direct interaction partners, and/or writing “group” (to indicate the presence of a large number of other people). Fourth, participants selected an activity from a drop-down list consisting of: “socializing”, “eating”, “exercising”, “watching TV”, “working”, “commuting”, “using computer/email/internet”, “preparing food”, “on the phone”, “praying/meditating/worship”, “napping”, “taking care of children”, “housework”, or “other”. Fifth, participants self-generated words to label their current affective experience. Specifically, participants were asked to “list any emotion(s) you were feeling when you received the prompt”. Participants were able to provide as many words as they felt necessary to describe their affective experience but were required to input at least one word. For each self-generated word, participants were asked to provide an intensity rating on a five-point scale: “not at all” (1), “a little” (2), “moderately” (3), “a lot” (4), “very much” (5). Finally, participants received one of two possible single-item decision tasks: either a temporal discounting problem or a scrambled anagram problem.

**Payment**

Participants received $30 for their first in-lab session, $20 per day for the first five days of experience sampling, $30 per day for the second five days of experience sampling, and $40 per day for the final four days of experience sampling. Participants were incentivized to respond to an average of eight prompts per day during experience sampling, and received a $10 bonus for every pay period in which they made this target (i.e., up to 3 times total). Lastly, participants received $50 for their second and final in-lab session. Participants also received a $25 bonus for completion of an in-lab temporal discounting task.

**In-Lab Tasks and Questionnaires**

Participants completed in-lab sessions before and after experience sampling, each 2-3 hours in length. During these sessions, participants completed a battery of questionnaires, as well as tasks related to cognitive functioning, decision making, and affective experience. Electrocardiogram (ECG), impedance cardiogram (ICG), electrodermal activity (EDA; recorded from the palm, as well as the back of the neck to correspond with ambulatory measurement), and respiration were captured throughout both sessions. As described below, continuous noninvasive arterial pressure (CNAP) and finger photoplethysmography (PPG) were also captured during select segments.

In the initial in-lab session, participants provided informed consent and were provided with an overview of the study. Participants were then hooked up to physiological monitoring equipment and asked to complete health and demographics forms. Participants were then instrumented with blood pressure cuffs (on the arm and finger) and finger PPG after which they sat quietly for a 5-minute resting baseline. The finger PPG was then removed, and participants completed a running letter span (RLS; Broadway & Engle, 2010) task. Both blood pressure cuffs were then removed, and participants completed temporal discounting^2,3^, anagrams^4,5^, and attentional network (ANT^6^) tasks. Next, participants completed a scenario immersion task^7,8^, in which they listened to a series of brief emotional scenarios and rated their felt affect on the dimensions of valence and arousal^9^. Lastly, participants completed the Session 1 questionnaires listed in Table S1. Upon completion of the session, participants were disconnected from the physiological equipment, instructed about the scheduling of their experience sampling days, and paid.

In the second and final in-lab session, participants were first instrumented with physiological monitoring equipment and then asked to complete the Session 2 questionnaires listed in Table S1. Participants were then instrumented with blood pressure cuffs and finger PPG and sat quietly for a 5-minute resting baseline. Immediately following the baseline, participants followed a sequence of stressor tasks. Specifically, participants completed mental math problems from the Trier Social Stress Test^10^ with the understanding that these would be used to assess personality and would be compared against other participants. Over three trials, participants received increasingly difficult mental math problems from a stern experimenter. After the first two trials, the finger PPG was removed and participants again completed the temporal discounting task^2,3^. After the third trial, blood pressure cuffs were removed and participants again completed another version of the anagrams task^4,5^. Participants were led to believe there would be a fourth mental math trial to maintain stress during the anagrams task; however, this additional trial was never administered. Following the stress sequence, participants again completed the attentional network task (ANT^6^) and a spatial arrangement task (SpAM^11^) in which they were asked to arrange emotion words according to their semantic similarity. Upon completion of the session, participants were disconnected from the physiological equipment, debriefed, and paid.

**End-of-Day Survey**

At the end of each experience sampling day, participants automatically received a modified day reconstruction survey^12^ to an email account they provided upon study enrollment. Participants were requested to complete the survey as soon as possible after finishing their day of experience sampling, and to avoid distractions while doing so.

In the survey, participants were presented with some of the information they provided for each of the prompts they completed during the day: the event time, label, social context, and major activity. Using this information as a guide, participants were asked to provide additional details about each experience sampling event. First, participants were asked to detail the social context of the event, including a brief description of any initials provided (e.g., “SB is a coworker”) and how well they knew their interaction partner(s). Second, participants were asked to provide a brief description of what was happening as they received the prompt, including their thoughts and feelings. Participants were requested to selectively detail three sampling events with a longer, more detailed description (>200 words). Next, participants were asked to recall their affective experience at the time of the prompt in two ways: (1) using slider scales to rate their valence and arousal, and (2) using 7-point Likert-style scales to rate their experienced intensity on a standard set of 18 emotions (“afraid”, “amused”, “angry”, “bored”, “calm”, “disgusted”, “embarrassed”, “excited”, “frustrated”, “grateful”, “happy”, “neutral”, “proud”, “relieved”, “sad”, “serene”, “surprised”, “worn out”). Lastly, participants were asked to respond to a series of seven questions developed based on the Geneva Appraisal Questionnaire^13^ that related to appraisal dimensions (e.g., goal relevance, power, control, coping, predictability^14^).

**Table S1.** Questionnaire Measures for In-Lab Sessions 1 and 2

| Questionnaire Name | Acronym | Reference | Session 1 | Session 2 |
| --- | --- | --- | --- | --- |
| Anxiety Sensitivity Index | ASI-3 | Taylor et al, 2007^15^ | x |  |
| Emotion Reactivity Scale | ERS | Nock et al, 2008^16^ | x |  |
| Need for Arousal Scale | NAS | Figner et al, 2009^17^ | x |  |
| Generalized Anxiety Disorder | GAD7 | Spitzer et al, 2006^18^ | x | x |
| Toronto Alexithymia Scale, 20-item version | TAS-20 | Bagby et al, 1994^19^ | x | x |
| Range and Differentiation of Emotional Experience Scale | RDEES | Kang & Shaver, 2004^20^ | x | x |
| Perceived Stress Scale, 4-item version | PSS4 | Cohen et al, 1983^21^ | x | x |
| Patient Health Questionnaire, Severity of Somatic Symptoms scale | PHQ-15 | Kroenke et al, 2002^22^ | x | x |
| Patient Health Questionnaire, Depression scale | PHQ-8 | Kroenke et al, 2009^23^ | x | x |
| Revised NEO Personality Inventory | NEO PI-R | Costa et al, 1992^24^ |  | x |
| Barratt Impulsiveness Scale | BIS | Barratt, 1985^25^ |  | x |
| Domain-Specific Risk Taking Questionnaire | DSRTQ | Weber et al, 2002^26^ |  | x |
| UCLA Loneliness Scale, 3-item version | UCLA-L53 | Hughes et al, 2004^27^ |  | x |
| Stimulating-Instrumental Risk Taking Questionnaire | SIRTQ | Zaleskiewicz, 2001^28^ |  | x |

**Physiological Signal Processing**

**Table S2.** Hyperparameters for Physiological Signal Processing

| Signal | Hyperparameter | Value | Reference Value | Reference Publication |
| --- | --- | --- | --- | --- |
| Electrocardiogram (ECG) | Length of signal for flatness check | 1.2*sampling frequency | 0.8*sampling frequency | Nabian et al (2018)^29^ |
|  | Minimum standard deviation for flatness check | 1.00E-05 | 1.00E-05 |  |
|  | Length for minimum-maximum check | 1.2*sampling frequency | 1.2*sampling frequency |  |
|  | Minimum/maximum for max/min check | -.005/.005 | -.005/.005 |  |
|  | Length of signal for skewness check | 1.2*sampling frequency | 1.2*sampling frequency |  |
|  | Length of sub-chunks for skewness check | .064*sampling frequency | .032*sampling frequency |  |
|  | Minimum skewness | .45 | .45 |  |
| Impedance cardiogram (ICG) | Window for point detection around R-peak | -250 ms to +500 ms | -250 ms to +500 ms | Nabian et al (2018)^29^ |
|  | Number of cycles for ensemble averaging | 8 | 8 |  |
|  | Maximum dZ/dt slope (d^2^Z/dt^2^) for d^3^Z/dt^3^ zero-crossings over first third of detected most prominent monotonically-increasing segment | Start threshold at 2*H/fs, increase in steps of 2 until > 1 zero-crossing; max 10*H/fs | 10*H/fs | Forouzanfar et al (2018)^30^ |
|  | Minimum d^3^Z/dt^3^ value for local maxima over first third of most prominent monotonically-increasing segment | 7*H/fs | 4*H/fs |  |

**Unsupervised Clustering Analyses**

**Table S3.** Hyperparameters for Dirichlet Process-Gaussian Mixture Modeling (DP-GMM)

| Hyperparameter | Value |
| --- | --- |
| Initial number of components | Number of events |
| Number of random restarts | 100 |
| Covariance type | Full |
| Initialization | K-means |
| Weight concentration prior | Dirichlet Process |
| Mean prior | Mean of data |

**Supplementary Results**

**Table S4.** Participant-Level Results from Within-Person Clustering Analysis

| **PP ID** | **Number Events** | **Number Used** | **Percent Used** | **Number Clusters** | **Percent Events Un-clustered** | **Number Unique Words** | **Mean Number Clusters per Word** | **Mean Percent Clusters per Word** | **Number Clusters Common Pattern** | **Percent Clusters Common Pattern** | **Mean Range Valence** | **Mean Range Arousal** |
| --- | --- | --- | --- | --- | --- | --- | --- | --- | --- | --- | --- | --- |
| 010 | 140 | 59 | 42.14 | 7 | 26.06 | 20 | 1.90 | 27.14 | 0 | 0.00 | 20.86 | 35.88 |
| 025 | 123 | 46 | 37.40 | 4 | 38.93 | 19 | 1.74 | 43.42 | 0 | 0.00 | 32.95 | 44.73 |
| 026 | 115 | 50 | 43.48 | 5 | 31.61 | 28 | 1.79 | 35.71 | 0 | 0.00 | 24.55 | 20.09 |
| 038 | 131 | 67 | 51.15 | 6 | 28.04 | 10 | 2.60 | 43.33 | 1 | 16.67 | 30.80 | 29.76 |
| 060 | 153 | 29 | 18.95 | 3 | 27.79 | 9 | 1.44 | 48.15 | 0 | 0.00 | 37.66 | 38.99 |
| 065 | 115 | 59 | 51.30 | 6 | 27.40 | 15 | 1.80 | 30.00 | 0 | 0.00 | 8.41 | 12.22 |
| 002 | 122 | 109 | 89.34 | 7 | 36.83 | 33 | 1.85 | 26.41 | 2 | 28.57 | 47.25 | 34.95 |
| 003 | 114 | 112 | 98.25 | 5 | 48.57 | 29 | 2.21 | 44.14 | 2 | 40.00 | 48.62 | 40.51 |
| 006 | 128 | 121 | 94.53 | 6 | 26.53 | 66 | 2.06 | 34.34 | 5 | 83.33 | 54.91 | 63.42 |
| 007 | 110 | 73 | 66.36 | 3 | 40.71 | 12 | 2.58 | 86.11 | 0 | 0.00 | 24.13 | 44.00 |
| 009 | 122 | 95 | 77.87 | 6 | 38.32 | 22 | 2.14 | 35.61 | 1 | 16.67 | 39.30 | 41.53 |
| 011 | 104 | 88 | 84.62 | 3 | 44.05 | 43 | 1.44 | 48.06 | 1 | 33.33 | 29.57 | 53.84 |
| 012 | 120 | 107 | 89.17 | 3 | 41.22 | 46 | 1.43 | 47.83 | 1 | 33.33 | 41.73 | 40.52 |
| 013 | 166 | 103 | 62.05 | 5 | 38.40 | 20 | 2.85 | 57.00 | 1 | 20.00 | 55.93 | 51.63 |
| 014 | 130 | 110 | 84.62 | 5 | 50.37 | 18 | 1.83 | 36.67 | 1 | 20.00 | 41.25 | 26.39 |
| 015 | 89 | 80 | 89.89 | 5 | 28.52 | 30 | 2.20 | 44.00 | 1 | 20.00 | 50.95 | 68.52 |
| 017 | 143 | 107 | 74.83 | 5 | 44.67 | 51 | 2.12 | 42.35 | 0 | 0.00 | 67.39 | 62.13 |
| 018 | 113 | 104 | 92.04 | 6 | 26.60 | 52 | 2.06 | 34.29 | 3 | 50.00 | 37.15 | 39.25 |
| 019 | 119 | 117 | 98.32 | 5 | 41.56 | 49 | 2.22 | 44.49 | 2 | 40.00 | 47.65 | 52.65 |
| 021 | 187 | 172 | 91.98 | 4 | 40.91 | 37 | 2.16 | 54.05 | 2 | 50.00 | 43.09 | 64.12 |
| 022 | 121 | 112 | 92.56 | 4 | 17.80 | 17 | 2.82 | 70.59 | 2 | 50.00 | 37.94 | 57.32 |
| 023 | 105 | 88 | 83.81 | 4 | 57.28 | 36 | 1.69 | 42.36 | 0 | 0.00 | 57.18 | 50.45 |
| 024 | 119 | 81 | 68.07 | 4 | 33.75 | 52 | 1.69 | 42.31 | 2 | 50.00 | 24.83 | 33.64 |
| 027 | 117 | 108 | 92.31 | 6 | 39.12 | 42 | 2.00 | 33.33 | 1 | 16.67 | 25.68 | 32.58 |
| 028 | 120 | 103 | 85.83 | 4 | 45.16 | 39 | 1.54 | 38.46 | 2 | 50.00 | 50.79 | 65.58 |
| 029 | 100 | 87 | 87.00 | 6 | 38.05 | 22 | 2.59 | 43.18 | 2 | 33.33 | 38.90 | 36.72 |
| 032 | 118 | 107 | 90.68 | 3 | 22.09 | 38 | 2.08 | 69.30 | 1 | 33.33 | 49.23 | 61.49 |
| 033 | 137 | 88 | 64.23 | 5 | 29.80 | 12 | 2.92 | 58.33 | 0 | 0.00 | 40.59 | 30.50 |
| 034 | 113 | 109 | 96.46 | 4 | 36.87 | 28 | 1.89 | 47.32 | 1 | 25.00 | 58.45 | 56.47 |
| 036 | 147 | 87 | 59.18 | 3 | 35.51 | 44 | 1.75 | 58.33 | 1 | 33.33 | 38.71 | 28.72 |
| 037 | 115 | 84 | 73.04 | 6 | 36.18 | 9 | 2.44 | 40.74 | 2 | 33.33 | 38.21 | 35.62 |
| 040 | 125 | 114 | 91.20 | 7 | 48.13 | 9 | 2.56 | 36.51 | 0 | 0.00 | 23.50 | 22.53 |
| 043 | 115 | 92 | 80.00 | 5 | 53.72 | 140 | 1.35 | 27.00 | 2 | 40.00 | 34.72 | 46.13 |
| 045 | 105 | 86 | 81.90 | 5 | 51.28 | 20 | 2.00 | 40.00 | 2 | 40.00 | 20.40 | 36.33 |
| 046 | 151 | 130 | 86.09 | 5 | 54.03 | 18 | 2.06 | 41.11 | 3 | 60.00 | 73.98 | 60.21 |
| 047 | 143 | 97 | 67.83 | 3 | 43.33 | 16 | 2.25 | 75.00 | 1 | 33.33 | 67.02 | 54.25 |
| **PP ID** | **Number Events** | **Number Used** | **Percent Used** | **Number Clusters** | **Percent Events Un-clustered** | **Number Unique Words** | **Mean Number Clusters per Word** | **Mean Percent Clusters per Word** | **Number Clusters Common Pattern** | **Percent Clusters Common Pattern** | **Mean Range Valence** | **Mean Range Arousal** |
| 049 | 116 | 109 | 93.97 | 5 | 47.93 | 109 | 1.60 | 31.93 | 1 | 20.00 | 52.39 | 46.47 |
| 050 | 123 | 105 | 85.37 | 5 | 35.21 | 16 | 2.00 | 40.00 | 1 | 20.00 | 50.55 | 45.66 |
| 051 | 141 | 129 | 91.49 | 6 | 32.48 | 23 | 2.61 | 43.48 | 1 | 16.67 | 54.80 | 73.62 |
| 054 | 132 | 122 | 92.42 | 4 | 32.38 | 50 | 1.88 | 47.00 | 0 | 0.00 | 53.50 | 57.98 |
| 055 | 122 | 103 | 84.43 | 5 | 42.30 | 21 | 2.14 | 42.86 | 3 | 60.00 | 44.95 | 49.05 |
| 056 | 120 | 81 | 67.50 | 8 | 26.43 | 28 | 1.86 | 23.21 | 2 | 25.00 | 26.77 | 30.86 |
| 057 | 160 | 126 | 78.75 | 4 | 35.84 | 16 | 2.00 | 50.00 | 2 | 50.00 | 44.05 | 28.03 |
| 058 | 101 | 70 | 69.31 | 5 | 36.65 | 16 | 2.19 | 43.75 | 2 | 40.00 | 47.71 | 64.43 |
| 059 | 125 | 86 | 68.80 | 3 | 48.11 | 28 | 1.21 | 40.48 | 0 | 0.00 | 80.92 | 45.58 |
| 061 | 119 | 70 | 58.82 | 4 | 19.21 | 21 | 1.76 | 44.05 | 1 | 25.00 | 34.88 | 43.82 |
| 062 | 89 | 81 | 91.01 | 3 | 37.18 | 27 | 1.85 | 61.73 | 1 | 33.33 | 72.59 | 58.36 |
| 063 | 129 | 120 | 93.02 | 7 | 32.29 | 25 | 2.32 | 33.14 | 2 | 28.57 | 38.86 | 25.06 |
| 064 | 125 | 81 | 64.80 | 6 | 33.78 | 13 | 2.69 | 44.87 | 1 | 16.67 | 25.80 | 32.13 |
| 066 | 120 | 105 | 87.50 | 4 | 37.98 | 48 | 1.75 | 43.75 | 1 | 25.00 | 77.42 | 74.27 |
| 067 | 113 | 106 | 93.81 | 4 | 35.93 | 31 | 1.87 | 46.77 | 2 | 50.00 | 30.23 | 39.27 |
| 068 | 113 | 112 | 99.12 | 4 | 34.97 | 13 | 2.62 | 65.38 | 1 | 25.00 | 64.89 | 62.80 |
| *Mean* | 123.17 | 101.67 | 82.92 | 4.76 | 38.22 | 33.37 | 2.07 | 45.69 | 1.41 | 29.76 | 45.86 | 47.16 |
| *SD* | 18.56 | 19.02 | 11.66 | 1.25 | 8.91 | 24.29 | 0.41 | 12.81 | 0.98 | 18.66 | 15.06 | 13.90 |
| *Median* | 120 | 104.50 | 85.96 | 5 | 37.58 | 28 | 2.06 | 43.62 | 1.00 | 30.95 | 44.50 | 45.90 |
| *Min* | 89 | 70 | 58.82 | 3 | 17.80 | 9 | 1.21 | 23.21 | 0.00 | 0.00 | 20.40 | 22.53 |
| *Max* | 187 | 172 | 99.12 | 8 | 57.28 | 140 | 2.92 | 86.11 | 5.00 | 83.33 | 80.92 | 74.27 |
| *Range* | 98 | 102 | 40.30 | 5 | 39.48 | 131 | 1.70 | 62.90 | 5.00 | 83.33 | 60.52 | 51.74 |
| *Mean* | 123.90 | 95.90 | 78.05 | 4.81 | 37.27 | 31.46 | 2.05 | 44.80 | 1.27 | 26.64 | 43.55 | 45.21 |
| *SD* | 18.18 | 24.44 | 17.86 | 1.27 | 8.91 | 23.54 | 0.41 | 12.56 | 1.01 | 19.69 | 15.89 | 14.66 |
| *Median* | 120 | 103.00 | 84.62 | 5 | 36.85 | 26 | 2 | 43.38 | 1.00 | 25.00 | 41.49 | 44.37 |
| *Min* | 89 | 29 | 18.95 | 3 | 17.80 | 9 | 1.21 | 23.21 | 0.00 | 0.00 | 8.41 | 12.22 |
| *Max* | 187 | 172 | 99.12 | 8 | 57.28 | 140 | 2.92 | 86.11 | 5.00 | 83.33 | 80.92 | 74.27 |
| *Range* | 98 | 143 | 80.17 | 5 | 39.48 | 131 | 1.70 | 62.90 | 5.00 | 83.33 | 72.51 | 62.05 |

Note: Dark gray rows at top denote the six participants with fewer than 70 usable events (i.e., no physiological data devoid of major artifact); these participants were excluded from the main analyses. Summary statistics for all participant-level results are provided at the bottom of the table in light gray rows, for the 46 participants included in the main analyses, and in dark gray rows, for all 52 participants. “Number Clusters Common Pattern” and “Percent Clusters Common Pattern” represent the number and percent, respectively, of a given participant’s clusters that corresponded with the top 10 common patterns of change in physiological activity. “Mean Range Valence” and “Mean Range Arousal” represent the average range in ratings across events in all clusters for a given participant.

**Table S5.** Cluster-Level Results from Within-Person Clustering Analysis

| **PP ID** | **Cluster** | **Percent Events** | **Number Events** | **Percent Sitting** | **Percent Standing** | **Percent Reclining** | **Percent Words** | **Mean Valence** | **Mean Arousal** | | | **IBI Change** | | **RSA Change** | **PEP Change** | **LVET Change** | **SV Change** | **CO Change** | **Common Pattern** | **Autonomic Control Mode** |
| --- | --- | --- | --- | --- | --- | --- | --- | --- | --- | --- | --- | --- | --- | --- | --- | --- | --- | --- | --- | --- |
| 010 | A | 25.73 | 15.67 | 60.74 | 38.25 | 0 | 28.5 | 6.54 | 12.95 | | | 0.562 | | -0.777 | 0.07 | -0.246 | 0.074 | 0.24 |  | uncoupled |
| 010 | B | 10.62 | 5.48 | 54.49 | 36.22 | 0 | 8.83 | 11.42 | 16.77 | | | -0.088 | | -0.513 | 0.034 | 0.727 | 0.153 | 0.104 |  | uncoupled |
| 010 | C | 8.68 | 5.66 | 70.29 | 29.11 | 0 | 10.71 | 17.59 | 7.42 | | | 0.651 | | 1.25 | -0.195 | 0.651 | -0.454 | -0.106 |  | coactivation |
| 010 | D | 8.42 | 4.24 | 94.07 | 0 | 0 | 6.82 | 8.21 | 15.01 | | | 0.59 | | 0.321 | 0.04 | -0.162 | -1.063 | -0.728 |  | uncoupled |
| 010 | E | 7.49 | 4.01 | 99.62 | 0 | 0 | 6.44 | 20.94 | 5.28 | | | -0.407 | | -0.283 | 1.925 | -0.138 | -0.4 | -0.499 |  | coactivation |
| 010 | F | 6.90 | 5.96 | 99.76 | 0 | 0 | 9.6 | 13.63 | -15.84 | | | -1.184 | | -1.05 | 0.221 | -0.662 | -1.384 | -1.548 |  | coactivation |
| 010 | G | 6.10 | 3.89 | 99.03 | 0 | 0 | 6.23 | 17.30 | 21.38 | | | -0.52 | | -0.017 | -0.02 | -0.686 | 0.096 | -0.06 |  | negligible change |
| 025 | A | 25.79 | 12.99 | 91.86 | 0 | 7.7 | 30.53 | 14.46 | -4.48 | | | -0.095 | | -0.916 | -0.322 | 0.12 | -0.061 | -0.122 |  | reciprocal |
| 025 | B | 21.27 | 12.00 | 66.29 | 33.31 | 0 | 28.56 | 15.12 | -1.83 | | | -0.041 | | 0.365 | -0.361 | -0.042 | -0.382 | -0.315 |  | coactivation |
| 025 | C | 7.61 | 3.02 | 98.99 | 0 | 0 | 6.15 | 12.01 | -7.04 | | | -0.423 | | 0.418 | 0.489 | -0.192 | -0.555 | -0.702 |  | reciprocal |
| 025 | D | 6.40 | 2.01 | 99.51 | 0 | 0 | 4.08 | 2.55 | 0.44 | | | 0.572 | | -0.299 | -0.074 | 0.366 | 0.404 | 0.565 |  | uncoupled |
| 026 | A | 20.40 | 9.64 | 87.96 | 0 | 10.38 | 17.11 | -4.22 | -11.43 | | | -0.003 | | -0.792 | 0.066 | -0.083 | 0.026 | -0.063 |  | uncoupled |
| 026 | B | 14.70 | 6.89 | 57.74 | 29.02 | 0 | 15.2 | -2.08 | -13.24 | | | -0.671 | | -0.82 | -0.208 | -0.796 | -0.557 | -0.53 |  | reciprocal |
| 026 | C | 14.19 | 6.25 | 91.04 | 0 | 0 | 10.75 | 1.35 | -13.21 | | | -0.262 | | 0.124 | 0.032 | 0.085 | 0.104 | 0.099 |  | negligible change |
| 026 | D | 14.06 | 7.11 | 68.4 | 27.88 | 0 | 15.64 | 0.79 | -4.53 | | | 0.559 | | 0.459 | 0.416 | 0.045 | 0.039 | 0.113 |  | reciprocal |
| 026 | E | 5.04 | 2.06 | 48.63 | 0 | 48.63 | 3.22 | 12.61 | -16.94 | | | 0.107 | | -0.14 | -0.243 | -0.107 | -0.501 | -0.355 |  | uncoupled |
| 038 | A | 31.10 | 20.83 | 68.7 | 28.69 | 0 | 31.63 | -4.28 | -6.52 | | | 0.53 | | 0.19 | -0.19 | 0.20 | 0.23 | 0.47 | 2 | coactivation |
| 038 | B | 12.48 | 7.62 | 51.62 | 47.8 | 0 | 11.04 | -4.17 | -5.37 | | | -0.51 | | -0.33 | 0.25 | 0.59 | 0.51 | 0.12 |  | coactivation |
| 038 | C | 9.23 | 7.03 | 41.24 | 56.81 | 0 | 10.19 | 0.43 | -2.83 | | | 0.76 | | -0.42 | 0.13 | -0.56 | -0.19 | 0.23 |  | uncoupled |
| 038 | D | 7.91 | 5.02 | 95 | 0 | 0 | 7.27 | -9.13 | -11.30 | | | 0.19 | | 0.14 | 0.19 | 0.20 | -0.41 | -0.11 |  | uncoupled |
| 038 | E | 6.01 | 4.04 | 74.23 | 0 | 24.78 | 5.84 | -9.18 | -3.98 | | | -0.83 | | -0.53 | -0.04 | -0.51 | -0.11 | -0.67 |  | uncoupled |
| 038 | F | 5.23 | 5.35 | 74.03 | 18.53 | 0 | 9.2 | -1.14 | -3.61 | | | 0.12 | | -1.42 | 0.06 | -0.24 | -0.30 | -0.11 |  | uncoupled |
| 060 | A | 50.43 | 14.15 | 28.26 | 28.26 | 42.25 | 47.18 | 1.31 | -3.08 | | | 0.327 | | 0.095 | -0.041 | 0.367 | 0.312 | 0.296 |  | uncoupled |
| 060 | B | 15.94 | 4.84 | 41.13 | 41.31 | 17.19 | 16.12 | 4.22 | 11.49 | | | -0.604 | | -0.196 | 0.508 | 0.038 | -0.463 | -0.493 |  | uncoupled |
| 060 | C | 5.85 | 1.00 | 99.76 | 0 | 0 | 3.33 | 6.98 | 2.00 | | | -0.268 | | -1.043 | -0.081 | 0.766 | 0.202 | 0.045 |  | coactivation |
| 065 | A | 20.12 | 11.28 | 67.72 | 0 | 29.74 | 22.6 | 17.29 | 12.12 | | | 0.048 | | 0.325 | 0.13 | 0.15 | -0.19 | -0.127 |  | uncoupled |
| 065 | B | 18.55 | 10.84 | 36.09 | 9.17 | 53.35 | 19.94 | 21.22 | 8.91 | | | -0.965 | | -0.388 | -0.533 | -0.462 | -0.078 | -0.462 |  | coactivation |
| 065 | C | 14.30 | 9.05 | 77.29 | 0 | 21.73 | 15.33 | 7.14 | 28.77 | | | 0.229 | | 0.001 | -1.632 | 0.422 | 0.302 | 0.329 |  | coactivation |
| 065 | D | 8.41 | 4.25 | 70.31 | 0 | 22.69 | 7.2 | 19.98 | 11.21 | | | 0.501 | | -0.668 | 0.02 | 0.072 | -0.015 | 0.15 |  | negligible change |
| 065 | E | 5.77 | 4.04 | 24.75 | 49.5 | 24.73 | 10.23 | 21.82 | 37.84 | | | -0.029 | | -1.312 | -0.775 | -0.305 | 0.832 | 0.63 |  | reciprocal |
| 065 | F | 5.45 | 3.02 | 66.22 | 0 | 33.13 | 5.09 | 34.18 | 8.40 | | | -0.525 | | -0.181 | 0.055 | 1.676 | 0.525 | 0.224 |  | coactivation |
| 002 | A | 17.73 | 21.99 | 63.7 | 25.58 | 8.68 | 18.88 | 11.98 | 18.50 | | | 0.19 | | -0.86 | 0.15 | 0.16 | 0.13 | 0.18 |  | uncoupled |
| 002 | B | 11.65 | 15.58 | 58.14 | 30.68 | 6.42 | 13.52 | 9.66 | 13.34 | | | 0.36 | | 0.09 | -0.15 | 0.30 | 0.50 | 0.51 | 10 | negligible change |
| 002 | C | 9.38 | 10.21 | 68 | 29.39 | 0 | 8.35 | 9.76 | 12.03 | | | -0.34 | | -0.17 | -0.26 | 1.85 | 1.26 | 0.62 |  | reciprocal |
| 002 | D | 8.38 | 8.58 | 57.6 | 34.93 | 0 | 7.84 | 20.33 | 16.01 | | | -0.52 | | -1.58 | -0.04 | 0.27 | -0.06 | -0.32 |  | uncoupled |
| 002 | E | 5.71 | 6.30 | 60.05 | 15.76 | 15.85 | 5.97 | 15.99 | 9.16 | | | -1.04 | | -0.23 | -0.07 | -0.45 | -0.76 | -1.20 | 3 | uncoupled |
| 002 | F | 5.24 | 4.88 | 95.38 | 0 | 0 | 4.8 | 22.65 | 11.46 | | | -0.67 | | -0.52 | 1.37 | -0.49 | -0.41 | -0.71 |  | coactivation |
| 002 | G | 5.09 | 7.22 | 96.01 | 0 | 0 | 7.54 | 14.11 | 5.76 | | | -1.08 | | 0.52 | -0.18 | -0.06 | 0.24 | -0.98 |  | coactivation |
| 003 | A | 15.80 | 16.87 | 80.27 | 15.62 | 0 | 13.7 | 16.11 | 14.30 | | | -0.07 | | -0.64 | -0.26 | 0.04 | 0.07 | 0.02 |  | reciprocal |
| 003 | B | 10.34 | 14.15 | 61.57 | 28.26 | 7.06 | 11.87 | 17.72 | 12.54 | | | 0.91 | | -1.16 | -0.02 | 0.28 | 0.18 | 0.72 | 7 | uncoupled |
| 003 | C | 10.22 | 12.35 | 49.61 | 40.26 | 0 | 11.5 | 17.64 | 17.95 | | | 0.50 | | -0.14 | -0.39 | 0.48 | 0.63 | 0.71 | 9 | uncoupled |
| **Table S5.** Cluster-Level Results (Continued) | | | | | | | | | | |  |  |  |  |  |  |  |  |  |  |
| **PP ID** | **Cluster** | **Percent Events** | **Number Events** | **Percent Sitting** | **Percent Standing** | **Percent Reclining** | **Percent Words** | **Mean Valence** | **Mean Arousal** | | | **IBI Change** | | **RSA Change** | **PEP Change** | **LVET Change** | **SV Change** | **CO Change** | **Common Pattern** | **Autonomic Control Mode** |
| 003 | D | 9.93 | 11.12 | 80.35 | 8.95 | 8.99 | 10.32 | 11.86 | 10.54 | | | -0.71 | | 0.57 | -0.04 | -0.08 | -0.33 | -0.63 |  | uncoupled |
| 003 | E | 5.13 | 7.06 | 70.73 | 28.13 | 0 | 5.39 | 23.42 | 16.45 | | | 0.42 | | -0.09 | 0.00 | 0.20 | -0.86 | -0.44 |  | negligible change |
| 006 | A | 27.43 | 32.77 | 83.74 | 2.98 | 6.1 | 28.91 | 19.82 | 17.51 | | | 0.70 | | -0.70 | 0.07 | 0.13 | -0.06 | 0.34 | 8 | uncoupled |
| 006 | B | 13.63 | 17.72 | 87.89 | 0 | 5.15 | 15.09 | 25.37 | 22.80 | | | 0.48 | | 0.39 | 0.16 | 0.17 | 0.26 | 0.47 |  | reciprocal |
| 006 | C | 12.93 | 14.79 | 94.16 | 0 | 0 | 14.08 | 25.16 | 23.77 | | | 0.53 | | -0.42 | -0.52 | 0.38 | 1.13 | 1.03 | 4 | reciprocal |
| 006 | D | 8.10 | 10.40 | 86.48 | 9.58 | 0 | 10.9 | 24.64 | 12.10 | | | 0.66 | | 0.29 | -0.53 | 1.56 | 0.62 | 0.78 | 2 | coactivation |
| 006 | E | 6.29 | 7.08 | 97.27 | 0 | 0 | 4.82 | 20.87 | 10.73 | | | 1.15 | | 0.15 | -0.42 | 0.77 | 0.59 | 1.22 | 9 | uncoupled |
| 006 | F | 5.09 | 7.19 | 68.01 | 27.83 | 0 | 5.65 | 26.78 | 30.97 | | | 1.03 | | -0.33 | 0.00 | -0.01 | 1.06 | 1.08 | 6 | uncoupled |
| 007 | A | 26.16 | 19.06 | 74.44 | 24.4 | 0 | 26.24 | 23.86 | 25.48 | | | 0.00 | | -0.29 | -0.01 | 0.07 | 0.01 | 0.01 |  | uncoupled |
| 007 | B | 23.75 | 16.57 | 66.06 | 29.44 | 0 | 19.94 | 25.50 | 18.23 | | | 1.02 | | -0.21 | -0.13 | -0.21 | -0.20 | 0.11 |  | uncoupled |
| 007 | C | 9.37 | 10.07 | 88.19 | 9.93 | 0 | 18.51 | 27.04 | 23.81 | | | 0.27 | | 0.76 | 0.02 | 0.05 | 0.67 | 0.64 |  | uncoupled |
| 009 | A | 17.67 | 15.97 | 88.73 | 3.06 | 6.26 | 15.95 | 13.00 | -0.35 | | | 0.66 | | 0.41 | -0.44 | 0.48 | 0.78 | 0.88 | 2 | coactivation |
| 009 | B | 12.69 | 11.65 | 75.39 | 0 | 16.66 | 13.6 | 16.11 | 3.21 | | | 0.87 | | 0.38 | -0.31 | 0.27 | 0.15 | 0.81 |  | coactivation |
| 009 | C | 10.74 | 12.47 | 78.73 | 15.91 | 0 | 13.48 | 13.59 | 14.33 | | | -0.66 | | 0.07 | 0.28 | -0.30 | -0.13 | -0.48 |  | uncoupled |
| 009 | D | 7.12 | 13.56 | 81.25 | 14.75 | 0 | 14.56 | 14.22 | 5.77 | | | -0.34 | | -0.26 | 0.30 | -0.19 | -0.57 | -0.64 |  | coactivation |
| 009 | E | 7.06 | 8.28 | 72.33 | 24.07 | 0 | 8.25 | 4.40 | 1.45 | | | 1.09 | | 0.27 | 0.12 | -0.18 | 0.15 | 0.58 |  | uncoupled |
| 009 | F | 6.40 | 8.82 | 77.9 | 0 | 9.82 | 8.81 | 12.41 | 10.09 | | | -0.10 | | -0.72 | 0.23 | -0.08 | -0.14 | -0.16 |  | coactivation |
| 011 | A | 39.22 | 34.34 | 90.34 | 2.84 | 5.67 | 35.79 | 10.79 | 6.95 | | | 0.11 | | -0.14 | 0.08 | 0.08 | 0.08 | 0.08 |  | negligible change |
| 011 | B | 9.20 | 7.18 | 77.83 | 0 | 13.92 | 7.49 | 2.72 | 18.10 | | | 0.58 | | 0.08 | -0.11 | -0.30 | -0.30 | -0.12 |  | negligible change |
| 011 | C | 7.54 | 5.94 | 97.65 | 0 | 0 | 6.52 | 7.15 | 2.37 | | | 1.18 | | 0.27 | -0.77 | 0.44 | 0.81 | 0.99 | 2 | coactivation |
| 012 | A | 32.85 | 34.87 | 74.19 | 4.96 | 16.71 | 31.69 | 11.25 | 6.70 | | | -0.06 | | -0.05 | -0.05 | 0.04 | 0.01 | -0.06 |  | negligible change |
| 012 | B | 14.80 | 16.63 | 52.18 | 38.42 | 6 | 15.15 | 7.46 | 11.46 | | | 0.31 | | -1.10 | 0.22 | -0.12 | -0.37 | -0.01 |  | coactivation |
| 012 | C | 11.13 | 11.02 | 41.98 | 35.59 | 18.14 | 10.92 | 15.63 | 13.23 | | | -0.78 | | -0.69 | 0.12 | -0.43 | -0.28 | -0.53 | 3 | uncoupled |
| 013 | A | 21.41 | 24.21 | 84.37 | 8.23 | 4.01 | 22.21 | 8.67 | 14.93 | | | 0.29 | | -0.95 | -0.02 | -0.23 | -0.12 | 0.06 |  | uncoupled |
| 013 | B | 13.15 | 15.83 | 86.45 | 6.29 | 0 | 15.72 | 7.31 | 8.18 | | | -0.81 | | -0.12 | -0.90 | -0.28 | -0.25 | -0.51 |  | uncoupled |
| 013 | C | 10.71 | 10.58 | 95.18 | 0 | 0 | 8.85 | 10.71 | 9.16 | | | 0.66 | | -0.01 | 0.21 | 0.07 | 0.54 | 0.85 |  | uncoupled |
| 013 | D | 10.54 | 11.23 | 77.05 | 17.7 | 0 | 11.16 | 8.01 | 11.46 | | | -0.22 | | 0.52 | 0.38 | -0.27 | -0.62 | -0.57 |  | reciprocal |
| 013 | E | 5.79 | 5.14 | 72.45 | 17.72 | 0 | 5.37 | 4.60 | 25.62 | | | 0.78 | | 0.18 | -1.01 | 0.84 | 0.52 | 0.89 | 2 | coactivation |
| 014 | A | 18.97 | 21.10 | 82.39 | 9.04 | 4.16 | 19.18 | 17.85 | -17.89 | | | -0.46 | | -0.44 | -0.15 | 0.12 | 0.11 | -0.15 |  | reciprocal |
| 014 | B | 9.39 | 10.09 | 59.99 | 28.71 | 7.98 | 9.17 | 16.90 | -23.49 | | | 0.17 | | 0.63 | 0.15 | -0.14 | -0.43 | -0.17 |  | reciprocal |
| 014 | C | 9.01 | 11.97 | 64.02 | 33.17 | 0 | 10.87 | 17.32 | -15.23 | | | 0.58 | | -0.05 | 0.63 | -0.52 | 0.06 | 0.28 |  | uncoupled |
| 014 | D | 6.76 | 6.63 | 59.33 | 30.15 | 0 | 6.03 | 28.62 | -20.69 | | | 0.50 | | 0.43 | -0.15 | 1.13 | 0.55 | 0.60 | 1 | uncoupled |
| 014 | E | 5.50 | 6.48 | 60.18 | 15.43 | 14.53 | 5.88 | 19.31 | -24.41 | | | 0.30 | | 0.06 | 0.17 | 0.04 | -0.01 | 0.21 |  | uncoupled |
| 015 | A | 26.24 | 20.52 | 82.36 | 13.8 | 0 | 27.08 | 12.20 | 4.97 | | | -0.13 | | 0.12 | -0.01 | -0.57 | -0.22 | -0.16 |  | negligible change |
| 015 | B | 15.39 | 13.00 | 74.43 | 23.04 | 0 | 14.92 | 19.16 | 7.65 | | | 0.18 | | -0.38 | -0.10 | 0.37 | 0.08 | 0.14 |  | uncoupled |
| 015 | C | 15.27 | 11.96 | 88.51 | 0 | 8.36 | 14.35 | 12.29 | 5.48 | | | 1.12 | | 0.46 | 0.07 | 0.47 | 0.96 | 1.24 | 1 | uncoupled |
| 015 | D | 8.09 | 6.01 | 82.76 | 16.63 | 0 | 7.91 | 8.72 | -4.62 | | | -0.64 | | 0.75 | -1.09 | -0.29 | -0.06 | -0.48 |  | coactivation |
| 015 | E | 6.49 | 6.43 | 61.56 | 30.46 | 0 | 8.28 | 10.10 | 3.28 | | | 0.28 | | -0.06 | 0.25 | 0.34 | -0.31 | -0.05 |  | uncoupled |
| 017 | A | 17.17 | 17.73 | 95.28 | 0 | 0 | 16.48 | 5.10 | 0.62 | | | 0.77 | | -0.59 | 0.26 | -0.32 | -0.25 | 0.00 |  | coactivation |
| 017 | B | 11.43 | 12.23 | 79.74 | 16.36 | 0 | 12.62 | 6.91 | 16.09 | | | -0.64 | | 0.31 | 0.10 | -0.54 | -0.16 | -0.32 |  | uncoupled |
| 017 | C | 10.67 | 11.04 | 44.22 | 9.06 | 18.12 | 10.58 | 5.35 | -1.35 | | | 0.59 | | -0.43 | 0.19 | 0.23 | 0.57 | 0.79 |  | coactivation |
| **Table S5.** Cluster-Level Results (Continued) | | | | | | | | | | |  |  |  |  |  |  |  |  |  |  |
| **PP ID** | **Cluster** | **Percent Events** | **Number Events** | **Percent Sitting** | **Percent Standing** | **Percent Reclining** | **Percent Words** | **Mean Valence** | **Mean Arousal** | | | **IBI Change** | | **RSA Change** | **PEP Change** | **LVET Change** | **SV Change** | **CO Change** | **Common Pattern** | **Autonomic Control Mode** |
| 017 | D | 8.97 | 10.05 | 78.93 | 0 | 9.95 | 9.29 | 0.17 | -12.10 | | | -0.23 | | -1.17 | -0.12 | 0.23 | 0.24 | 0.15 |  | uncoupled |
| 017 | E | 7.09 | 8.35 | 75.12 | 16.02 | 7.9 | 8.71 | 7.82 | 0.82 | | | 0.85 | | 0.65 | 0.07 | 0.32 | 0.10 | 0.33 |  | uncoupled |
| 018 | A | 24.41 | 31.03 | 78.62 | 9.02 | 8.05 | 32.93 | 13.48 | 1.56 | | | 0.78 | | -0.33 | -0.13 | 0.18 | 0.27 | 0.55 | 7 | uncoupled |
| 018 | B | 18.89 | 20.55 | 56.98 | 14.56 | 9.02 | 17.79 | 7.05 | 4.42 | | | 0.50 | | -0.29 | 0.10 | 0.04 | 0.12 | 0.35 | 8 | uncoupled |
| 018 | C | 10.20 | 12.95 | 76.27 | 0 | 13.96 | 12.77 | 14.46 | 0.65 | | | -0.17 | | 0.89 | -0.43 | 0.35 | 0.50 | 0.32 |  | coactivation |
| 018 | D | 8.22 | 9.41 | 69.34 | 20.51 | 6.02 | 8.8 | 14.03 | 1.67 | | | -0.06 | | 0.24 | 0.14 | 0.15 | -0.45 | -0.38 |  | uncoupled |
| 018 | E | 6.36 | 5.68 | 60.02 | 17.56 | 17.6 | 5.47 | 18.81 | 11.69 | | | 0.66 | | -0.44 | 0.20 | -0.37 | -0.82 | -0.32 |  | coactivation |
| 018 | F | 5.32 | 5.15 | 95.77 | 0 | 0 | 5.63 | 14.85 | 3.07 | | | 0.74 | | -0.14 | -0.02 | 0.30 | 1.63 | 1.49 | 10 | negligible change |
| 019 | A | 22.11 | 26.35 | 81.02 | 11.15 | 3.46 | 22.11 | -4.13 | 12.13 | | | 0.48 | | -0.20 | -0.13 | -0.17 | 0.05 | 0.24 |  | uncoupled |
| 019 | B | 13.38 | 17.06 | 83.82 | 0 | 5.86 | 14.72 | 3.16 | 14.36 | | | -1.04 | | 0.16 | -0.53 | 0.19 | 0.08 | -0.54 |  | coactivation |
| 019 | C | 10.11 | 11.78 | 70.53 | 14.12 | 0 | 11.45 | -9.21 | 18.72 | | | 0.39 | | -0.75 | 0.08 | 0.32 | 0.26 | 0.40 | 7 | uncoupled |
| 019 | D | 7.13 | 9.59 | 79.44 | 0 | 10.43 | 7.54 | -6.74 | 18.78 | | | -0.56 | | 0.43 | 0.26 | -0.15 | -0.14 | -0.35 |  | reciprocal |
| 019 | E | 5.70 | 5.72 | 93.81 | 0 | 0 | 4.13 | -10.79 | 15.64 | | | 0.20 | | 0.73 | -0.01 | 0.40 | 0.69 | 0.63 | 1 | uncoupled |
| 021 | A | 28.42 | 52.41 | 70.01 | 11.93 | 13.65 | 32.01 | 6.31 | -0.19 | | | 0.63 | | -0.02 | 0.11 | 0.06 | 0.33 | 0.43 |  | negligible change |
| 021 | B | 16.65 | 33.21 | 59.13 | 4.79 | 24.88 | 18.07 | 6.51 | -9.09 | | | -0.53 | | -0.72 | -0.01 | -0.25 | -0.32 | -0.41 | 3 | uncoupled |
| 021 | C | 7.46 | 12.63 | 58.31 | 15.74 | 15.83 | 6.06 | -1.89 | -14.27 | | | -1.00 | | -0.78 | -0.02 | -0.34 | -0.91 | -1.23 | 3 | uncoupled |
| 021 | D | 6.55 | 13.51 | 53.65 | 14.3 | 6.32 | 8.31 | 7.21 | 9.69 | | | 0.87 | | 0.18 | -0.14 | -0.09 | -0.09 | 0.18 |  | uncoupled |
| 022 | A | 26.79 | 34.01 | 72.84 | 11.68 | 7.93 | 32.1 | 22.66 | 12.98 | | | 0.77 | | 0.21 | -0.11 | 0.51 | 0.33 | 0.42 | 1 | uncoupled |
| 022 | B | 26.48 | 32.76 | 87.72 | 5.8 | 0 | 27.25 | 17.61 | 16.23 | | | 0.17 | | -0.54 | -0.23 | -0.13 | 0.43 | 0.29 | 5 | reciprocal |
| 022 | C | 22.22 | 25.85 | 78.52 | 11.55 | 3.79 | 21.8 | 15.77 | 16.15 | | | -0.28 | | 0.04 | 0.41 | -0.65 | -0.59 | -0.42 |  | uncoupled |
| 022 | D | 6.71 | 6.58 | 75.9 | 0 | 14.89 | 7.43 | 26.25 | 20.84 | | | -0.29 | | -1.67 | -0.35 | 0.15 | 0.02 | -0.13 |  | reciprocal |
| 023 | A | 18.98 | 16.10 | 66.07 | 11.92 | 18.51 | 20.86 | 6.61 | -3.48 | | | 0.30 | | 0.52 | 0.02 | 0.15 | 0.21 | 0.33 |  | uncoupled |
| 023 | B | 9.55 | 8.00 | 45.66 | 5.78 | 45.99 | 9.03 | 4.08 | -18.05 | | | -0.72 | | 0.19 | -0.39 | -0.33 | -0.03 | -0.50 |  | coactivation |
| 023 | C | 8.12 | 7.06 | 54.41 | 14.12 | 28.02 | 8.41 | 3.03 | -7.86 | | | 0.58 | | -0.47 | -0.10 | -0.15 | -0.74 | -0.24 |  | uncoupled |
| 023 | D | 6.07 | 5.94 | 63.74 | 14.94 | 16.77 | 6.33 | 7.08 | -14.90 | | | 0.14 | | -0.81 | 0.23 | -0.31 | 0.46 | 0.40 |  | coactivation |
| 024 | A | 26.52 | 23.08 | 89.35 | 8.4 | 0 | 29.25 | 7.00 | 10.01 | | | 0.49 | | 0.15 | 0.03 | -0.09 | 0.01 | 0.29 |  | uncoupled |
| 024 | B | 26.05 | 20.62 | 84.85 | 9.59 | 4.8 | 25.41 | 8.99 | 12.63 | | | -0.78 | | -0.16 | -0.58 | -0.11 | -0.04 | -0.49 |  | reciprocal |
| 024 | C | 7.15 | 5.03 | 58.12 | 19.87 | 19.79 | 6.18 | 11.15 | 9.52 | | | 0.70 | | 0.66 | -0.66 | 0.64 | 1.74 | 1.53 | 2 | coactivation |
| 024 | D | 6.54 | 6.09 | 49.13 | 49.27 | 0 | 7.42 | 12.96 | 7.29 | | | 0.82 | | 0.19 | -0.14 | 0.88 | 0.72 | 0.83 | 1 | uncoupled |
| 027 | A | 20.98 | 24.65 | 84.66 | 6.77 | 4.06 | 21.99 | 9.58 | -0.31 | | | 0.08 | | -0.22 | 0.08 | 0.06 | -0.02 | 0.04 |  | uncoupled |
| 027 | B | 11.17 | 11.17 | 66.86 | 8.84 | 17.87 | 9.8 | 7.97 | -3.20 | | | 0.73 | | -0.49 | -0.19 | 0.12 | 0.37 | 0.78 | 5 | reciprocal |
| 027 | C | 9.84 | 11.22 | 67.16 | 16.07 | 8.37 | 10.78 | 13.52 | -0.34 | | | -0.81 | | -0.08 | -0.03 | -0.25 | -0.30 | -0.66 |  | negligible change |
| 027 | D | 7.02 | 9.09 | 64.74 | 11 | 22 | 8.79 | 13.65 | 3.20 | | | 1.09 | | -0.03 | 0.46 | -0.09 | -0.39 | 0.76 |  | uncoupled |
| 027 | E | 6.48 | 6.21 | 96.65 | 0 | 0 | 6.72 | 5.33 | -1.03 | | | -0.47 | | -0.35 | -1.14 | 0.53 | 0.89 | 0.54 |  | reciprocal |
| 027 | F | 5.39 | 8.04 | 73.71 | 24.56 | 0 | 7.51 | 8.70 | 2.12 | | | -0.11 | | 0.16 | -0.43 | 0.32 | -0.77 | -0.72 |  | coactivation |
| 028 | A | 25.31 | 26.15 | 89.54 | 6 | 0 | 26.19 | 16.45 | 5.10 | | | 0.49 | | 0.22 | -0.04 | 0.28 | 0.24 | 0.40 | 1 | uncoupled |
| 028 | B | 16.23 | 17.22 | 68.89 | 22.54 | 5.28 | 16.84 | 11.73 | 12.25 | | | 0.74 | | -0.31 | -0.56 | 0.32 | 0.24 | 0.36 | 4 | reciprocal |
| 028 | C | 7.34 | 11.49 | 92.57 | 0 | 0 | 10.61 | 15.75 | -11.59 | | | 1.10 | | 0.20 | 0.03 | -0.25 | 0.27 | 0.77 |  | uncoupled |
| 028 | D | 5.96 | 5.25 | 90.7 | 0 | 0 | 5.62 | 22.28 | 9.48 | | | -0.34 | | -0.18 | 0.50 | 0.14 | 0.13 | 0.01 |  | coactivation |
| 029 | A | 17.43 | 15.61 | 78.89 | 18.13 | 0 | 16.66 | 2.10 | 2.64 | | | 1.04 | | -0.55 | 0.13 | 0.10 | 0.35 | 0.76 | 6 | uncoupled |
| 029 | B | 12.95 | 10.98 | 88.76 | 8.76 | 0 | 13.33 | -6.28 | 4.37 | | | 0.11 | | 0.02 | -0.06 | -0.17 | -0.33 | -0.20 |  | negligible change |
| **Table S5.** Cluster-Level Results (Continued) | | | | | | | | | |  | | |  |  |  |  |  |  |  |  |
| **PP ID** | **Cluster** | **Percent Events** | **Number Events** | **Percent Sitting** | **Percent Standing** | **Percent Reclining** | **Percent Words** | **Mean Valence** | **Mean Arousal** | | | **IBI Change** | | **RSA Change** | **PEP Change** | **LVET Change** | **SV Change** | **CO Change** | **Common Pattern** | **Autonomic Control Mode** |
| 029 | C | 12.38 | 10.59 | 84.8 | 9.44 | 0 | 10.89 | 1.72 | 12.12 | | | 0.59 | | -0.03 | 0.14 | 0.03 | 1.27 | 1.12 |  | negligible change |
| 029 | D | 6.59 | 6.05 | 98.77 | 0 | 0 | 7.76 | -9.89 | -6.81 | | | 0.22 | | -0.25 | 0.05 | -1.85 | -0.82 | -0.57 |  | uncoupled |
| 029 | E | 6.36 | 6.42 | 62.31 | 31.17 | 0 | 7.35 | 15.20 | 13.35 | | | 0.71 | | -1.70 | 0.00 | 0.14 | 0.17 | 0.44 | 6 | uncoupled |
| 029 | F | 6.24 | 8.17 | 72.68 | 24.43 | 0 | 9.15 | -4.16 | 10.20 | | | -0.93 | | 0.37 | 0.01 | -0.42 | -0.49 | -0.88 |  | uncoupled |
| 032 | A | 36.12 | 38.43 | 70.4 | 19.37 | 2.29 | 37.52 | -3.12 | 9.01 | | | 0.00 | | -0.01 | -0.11 | -0.12 | 0.01 | -0.02 |  | negligible change |
| 032 | B | 31.20 | 33.97 | 79.09 | 11.77 | 0 | 30.78 | -1.48 | 7.78 | | | 0.66 | | -0.32 | 0.03 | 0.01 | -0.12 | 0.42 | 8 | uncoupled |
| 032 | C | 10.59 | 11.57 | 60.46 | 14.85 | 17.25 | 9.41 | 0.06 | 4.71 | | | 0.87 | | 0.13 | 0.31 | 0.12 | -0.04 | 0.25 |  | uncoupled |
| 033 | A | 27.50 | 23.45 | 65.16 | 16.32 | 10.54 | 26.64 | 18.08 | 20.65 | | | 0.03 | | 0.26 | -0.02 | -0.07 | 0.02 | 0.00 |  | uncoupled |
| 033 | B | 19.55 | 20.61 | 57.85 | 0 | 32.82 | 23.42 | 17.48 | 18.60 | | | -0.06 | | -0.22 | 0.00 | -0.11 | -0.11 | -0.14 |  | uncoupled |
| 033 | C | 9.84 | 8.49 | 58.43 | 35.34 | 0 | 9.64 | 17.73 | 21.76 | | | 0.58 | | -1.08 | -0.15 | -0.40 | 0.42 | 0.44 |  | uncoupled |
| 033 | D | 6.85 | 6.18 | 48.46 | 0 | 48.51 | 7.02 | 16.62 | 21.17 | | | -0.56 | | 0.53 | 0.37 | -0.47 | 0.04 | -0.09 |  | reciprocal |
| 033 | E | 6.47 | 5.03 | 79.43 | 19.8 | 0 | 5.7 | 10.88 | 10.51 | | | -0.15 | | -0.14 | 1.56 | -0.40 | -0.26 | -0.16 |  | uncoupled |
| 034 | A | 32.79 | 38.93 | 88.74 | 7.67 | 0 | 35.71 | 10.82 | 3.18 | | | 0.81 | | -0.75 | -0.07 | -0.08 | 0.28 | 0.68 | 6 | uncoupled |
| 034 | B | 12.08 | 17.34 | 86.93 | 0 | 11.16 | 15.91 | 14.54 | 7.28 | | | -0.13 | | -0.94 | -0.13 | 0.01 | -0.37 | -0.39 |  | uncoupled |
| 034 | C | 10.54 | 11.32 | 88 | 8.71 | 0 | 10.37 | 10.07 | 8.15 | | | -0.84 | | -0.06 | 0.05 | -0.69 | -0.83 | -1.09 |  | negligible change |
| 034 | D | 7.72 | 7.63 | 89 | 0 | 9.45 | 6.97 | 10.57 | 12.59 | | | -0.16 | | 0.27 | -0.27 | 0.89 | 0.34 | 0.23 |  | coactivation |
| 036 | A | 41.55 | 41.92 | 84.02 | 7.06 | 4.77 | 50.36 | 7.26 | 6.81 | | | -0.03 | | -0.04 | -0.08 | -0.01 | 0.08 | 0.08 |  | negligible change |
| 036 | B | 17.48 | 15.94 | 90.36 | 0 | 0 | 17.01 | 8.38 | 7.84 | | | -0.24 | | 0.52 | 0.57 | -0.22 | -0.39 | -0.44 |  | reciprocal |
| 036 | C | 5.46 | 4.09 | 94.77 | 0 | 0 | 4.52 | 12.67 | 10.98 | | | 0.65 | | 0.36 | -0.23 | 0.82 | 1.05 | 1.18 | 2 | coactivation |
| 037 | A | 17.89 | 15.02 | 78.75 | 6.66 | 13.27 | 18.05 | 35.10 | 12.31 | | | 0.61 | | -0.11 | -0.43 | -0.18 | 0.71 | 0.72 |  | uncoupled |
| 037 | B | 12.80 | 10.15 | 61.95 | 19.71 | 7.23 | 11.94 | 43.94 | 8.73 | | | 0.78 | | -1.55 | -0.26 | 0.09 | 0.45 | 0.63 | 5 | reciprocal |
| 037 | C | 11.37 | 9.53 | 41.91 | 20.09 | 30.74 | 11.11 | 40.74 | 9.61 | | | 1.07 | | -0.61 | 0.03 | -0.12 | -0.26 | 0.31 |  | uncoupled |
| 037 | D | 7.92 | 8.14 | 36.83 | 24.56 | 36.07 | 10.33 | 42.37 | 14.74 | | | 0.25 | | 0.03 | -0.33 | -0.04 | -0.64 | -0.37 |  | uncoupled |
| 037 | E | 7.49 | 7.14 | 27.63 | 0 | 68.56 | 8.03 | 37.35 | 12.46 | | | 0.82 | | -0.71 | -0.49 | 0.56 | 1.02 | 1.32 | 4 | reciprocal |
| 037 | F | 6.36 | 7.79 | 50.76 | 24.59 | 23.45 | 8.76 | 29.46 | 7.28 | | | -0.09 | | 0.42 | -0.17 | 0.27 | 0.41 | 0.25 |  | coactivation |
| 040 | A | 10.77 | 12.59 | 44.49 | 31.56 | 15.86 | 12.06 | 12.08 | 15.81 | | | 0.46 | | -0.58 | 0.06 | 0.36 | -0.46 | -0.09 |  | uncoupled |
| 040 | B | 7.79 | 8.59 | 52.88 | 45.59 | 0 | 6.81 | 14.92 | 19.41 | | | -0.60 | | -0.74 | 0.20 | 0.07 | -0.41 | -0.52 |  | coactivation |
| 040 | C | 7.16 | 7.60 | 62.66 | 5.04 | 25.18 | 6.67 | 14.30 | 14.14 | | | 0.91 | | -0.03 | 0.38 | -0.02 | 0.30 | 0.83 |  | uncoupled |
| 040 | D | 7.11 | 9.38 | 72.86 | 21.26 | 0 | 8.58 | 14.74 | 15.75 | | | -0.35 | | 0.24 | -0.10 | 1.17 | 0.37 | 0.12 |  | uncoupled |
| 040 | E | 6.57 | 12.35 | 39.74 | 49.04 | 7.54 | 10.6 | 17.06 | 19.67 | | | 0.40 | | -1.35 | 0.23 | -0.16 | -0.26 | -0.01 |  | coactivation |
| 040 | F | 6.30 | 6.86 | 72.89 | 22.51 | 0 | 6.28 | 9.20 | 13.68 | | | 0.33 | | -0.63 | -1.40 | -0.10 | -0.10 | 0.07 |  | reciprocal |
| 040 | G | 6.17 | 6.35 | 97.69 | 0 | 0 | 5.39 | 17.65 | 15.68 | | | -0.11 | | 0.28 | -0.26 | -0.73 | -0.23 | -0.18 |  | coactivation |
| 043 | A | 15.24 | 15.53 | 98.61 | 0 | 0 | 15.91 | 2.14 | 4.89 | | | 0.72 | | -0.38 | -0.76 | 0.52 | 0.49 | 0.81 | 4 | reciprocal |
| 043 | B | 12.49 | 12.31 | 96.87 | 0 | 0 | 13.62 | -1.26 | -4.87 | | | 0.33 | | -0.96 | -0.91 | -0.30 | -0.26 | 0.04 |  | reciprocal |
| 043 | C | 7.22 | 6.10 | 98.29 | 0 | 0 | 6.04 | 5.18 | -0.09 | | | -1.07 | | -0.06 | 0.02 | -0.83 | 0.15 | -0.44 |  | negligible change |
| 043 | D | 5.92 | 4.96 | 96.9 | 0 | 0 | 6.19 | 0.01 | 7.28 | | | 1.19 | | -0.04 | -1.06 | 0.38 | 0.82 | 1.05 | 9 | uncoupled |
| 043 | E | 5.41 | 6.04 | 99.36 | 0 | 0 | 6.31 | -4.34 | 10.30 | | | -0.78 | | 0.44 | -1.78 | -0.47 | 0.41 | -0.14 |  | coactivation |
| 045 | A | 13.12 | 12.89 | 98.17 | 0 | 0 | 16.88 | 2.42 | 4.34 | | | 0.45 | | -0.06 | -0.06 | 0.74 | 0.56 | 0.65 | 10 | negligible change |
| 045 | B | 12.03 | 11.17 | 85.69 | 0 | 8.93 | 13.34 | 3.93 | 10.81 | | | -0.54 | | 0.71 | 0.16 | -0.11 | 0.00 | -0.31 |  | reciprocal |
| 045 | C | 9.02 | 8.71 | 81.06 | 0 | 18.66 | 9.87 | 7.56 | 4.24 | | | -0.87 | | -0.13 | 0.21 | 0.14 | -0.15 | -0.59 |  | uncoupled |
| 045 | D | 7.60 | 6.57 | 89.82 | 0 | 0 | 7.3 | 2.68 | 7.22 | | | 0.95 | | 0.50 | -0.03 | 0.30 | 0.22 | 0.84 | 1 | uncoupled |
| **Table S5.** Cluster-Level Results (Continued) | | | | | | | | | | |  |  |  |  |  |  |  |  |  |  |
| **PP ID** | **Cluster** | **Percent Events** | **Number Events** | **Percent Sitting** | **Percent Standing** | **Percent Reclining** | **Percent Words** | **Mean Valence** | **Mean Arousal** | | | **IBI Change** | | **RSA Change** | **PEP Change** | **LVET Change** | **SV Change** | **CO Change** | **Common Pattern** | **Autonomic Control Mode** |
| 045 | E | 6.95 | 6.72 | 73.27 | 0 | 14.88 | 8.18 | 3.62 | 11.15 | | | -0.04 | | -0.71 | -0.10 | 0.13 | 0.12 | 0.10 |  | uncoupled |
| 046 | A | 14.58 | 18.14 | 84.75 | 4.93 | 5.49 | 13.21 | 9.48 | 26.11 | | | 0.71 | | -0.57 | -0.05 | -0.10 | 0.28 | 0.40 | 6 | uncoupled |
| 046 | B | 10.79 | 16.94 | 60.22 | 17.56 | 16.48 | 13.74 | 16.90 | 26.98 | | | 0.57 | | -0.06 | -0.27 | 0.08 | -0.13 | 0.17 |  | uncoupled |
| 046 | C | 8.40 | 13.20 | 66.96 | 0 | 22.47 | 10.84 | 7.50 | 23.99 | | | 0.47 | | -1.51 | -0.25 | 0.55 | 0.43 | 0.54 | 4 | reciprocal |
| 046 | D | 6.17 | 7.31 | 78.4 | 13.67 | 0 | 5.8 | 19.13 | 30.81 | | | 0.96 | | 0.25 | -0.47 | 0.66 | 0.64 | 0.70 | 2 | coactivation |
| 046 | E | 6.02 | 11.37 | 58.43 | 29.54 | 8.58 | 8.36 | 19.07 | 24.36 | | | 0.96 | | -0.17 | 0.12 | -0.36 | -0.03 | 0.45 |  | uncoupled |
| 047 | A | 25.60 | 25.15 | 51.23 | 35.72 | 11.69 | 24.92 | -2.72 | 0.11 | | | -0.38 | | 0.06 | 0.02 | -0.09 | -0.20 | -0.36 |  | negligible change |
| 047 | B | 16.29 | 16.15 | 48.79 | 33.91 | 12.37 | 16.09 | -10.31 | -4.86 | | | 0.74 | | 0.53 | -0.23 | -0.11 | -0.25 | 0.24 |  | coactivation |
| 047 | C | 14.79 | 15.50 | 75.88 | 22.5 | 0 | 15.71 | -1.22 | -5.37 | | | 0.76 | | -0.47 | -0.21 | 0.51 | 0.74 | 0.91 | 4 | reciprocal |
| 049 | A | 16.88 | 20.19 | 69.61 | 19.74 | 8 | 18.28 | 14.27 | -15.17 | | | -0.17 | | -1.02 | -0.04 | 0.02 | -0.08 | -0.16 |  | uncoupled |
| 049 | B | 13.99 | 26.13 | 74.47 | 10.64 | 7.01 | 26.02 | 18.27 | -16.25 | | | 0.86 | | -0.01 | 0.02 | 0.20 | 0.25 | 0.64 | 10 | negligible change |
| 049 | C | 8.47 | 9.38 | 78.25 | 17.72 | 0 | 8.56 | 7.35 | -22.08 | | | 0.49 | | 0.02 | 0.33 | -0.29 | -1.03 | -0.56 |  | uncoupled |
| 049 | D | 6.74 | 7.06 | 84.69 | 14.15 | 0 | 5.93 | 11.42 | -7.69 | | | 0.44 | | -0.43 | -1.63 | -0.03 | -0.53 | -0.07 |  | reciprocal |
| 049 | E | 5.99 | 8.14 | 23.8 | 55.19 | 12.19 | 7.5 | 12.13 | -10.89 | | | 0.84 | | 0.81 | -0.03 | 0.26 | -0.01 | 0.43 |  | uncoupled |
| 050 | A | 17.73 | 23.26 | 82.63 | 4.3 | 4.25 | 21.65 | 14.05 | 3.72 | | | -0.52 | | -0.34 | -0.24 | 0.20 | 0.27 | -0.01 |  | reciprocal |
| 050 | B | 15.54 | 15.50 | 56.44 | 12.74 | 20.66 | 13.85 | 13.53 | -4.75 | | | 0.00 | | -0.89 | -0.32 | -0.31 | -0.18 | -0.11 |  | reciprocal |
| 050 | C | 14.11 | 14.13 | 65.37 | 24.08 | 7.08 | 13.48 | 15.91 | 5.16 | | | 0.96 | | 0.21 | -0.01 | 0.40 | 0.50 | 0.66 | 1 | uncoupled |
| 050 | D | 9.90 | 10.65 | 83.33 | 0 | 13.52 | 11.28 | 22.40 | -1.33 | | | -0.33 | | 0.22 | -0.24 | -0.29 | -0.79 | -0.53 |  | coactivation |
| 050 | E | 7.52 | 8.30 | 47.25 | 24.06 | 23.92 | 7.41 | 16.12 | 4.54 | | | -0.15 | | 1.25 | 0.28 | 0.11 | 0.01 | -0.08 |  | reciprocal |
| 051 | A | 20.11 | 35.44 | 68.35 | 12.8 | 13.77 | 26.28 | 30.48 | 28.54 | | | 0.48 | | -0.65 | -0.16 | -0.14 | 0.30 | 0.49 | 5 | reciprocal |
| 051 | B | 17.74 | 22.61 | 73.3 | 13.19 | 4.23 | 19.47 | 29.00 | 21.91 | | | 0.56 | | -0.23 | 0.21 | 0.51 | 0.21 | 0.39 |  | coactivation |
| 051 | C | 9.43 | 13.50 | 77.1 | 0 | 13.63 | 12.44 | 29.26 | 35.10 | | | -0.89 | | -0.12 | -0.17 | 0.15 | 0.11 | -0.35 |  | uncoupled |
| 051 | D | 7.96 | 11.81 | 80.19 | 0 | 16.92 | 8.15 | 28.16 | 18.01 | | | -1.02 | | -0.01 | 0.19 | 0.08 | 0.22 | -0.16 |  | uncoupled |
| 051 | E | 6.24 | 8.02 | 55.69 | 24.6 | 12.47 | 5.25 | 38.79 | 37.76 | | | 1.01 | | 0.14 | 0.59 | -0.32 | -0.78 | -0.01 |  | uncoupled |
| 051 | F | 6.04 | 7.19 | 55.44 | 13.91 | 27.83 | 5.36 | 22.23 | 13.90 | | | -0.15 | | -0.49 | -0.40 | 0.59 | 0.18 | -0.15 |  | reciprocal |
| 054 | A | 25.55 | 32.02 | 64.56 | 12.01 | 20.22 | 24.68 | 1.64 | 3.31 | | | 0.18 | | 0.09 | -0.17 | 0.02 | -0.05 | 0.05 |  | uncoupled |
| 054 | B | 20.89 | 26.42 | 69.66 | 25.85 | 0 | 19.81 | 4.33 | 3.71 | | | 0.58 | | -0.07 | 0.37 | 0.15 | 0.43 | 0.79 |  | uncoupled |
| 054 | C | 15.54 | 18.10 | 35.57 | 44 | 13.13 | 14.06 | 10.19 | 4.49 | | | 0.66 | | -0.97 | -0.66 | 0.53 | -0.01 | 0.50 |  | reciprocal |
| 054 | D | 5.64 | 6.21 | 64.44 | 16.07 | 15.97 | 6.22 | 4.94 | 10.09 | | | -0.76 | | -0.75 | -0.16 | -0.09 | -0.35 | -0.88 |  | reciprocal |
| 055 | A | 16.83 | 17.71 | 83.72 | 11.15 | 0 | 17.03 | 4.99 | 6.72 | | | -0.20 | | -0.27 | 0.11 | -0.26 | -0.27 | -0.30 | 3 | uncoupled |
| 055 | B | 16.81 | 20.23 | 91.16 | 0 | 3.44 | 19.44 | 3.83 | 5.63 | | | 0.69 | | -0.46 | -0.14 | 0.80 | 0.62 | 0.79 | 7 | uncoupled |
| 055 | C | 11.44 | 11.41 | 72.2 | 8.77 | 8.77 | 10.95 | 6.12 | 6.54 | | | 0.22 | | 0.82 | -0.19 | 0.13 | 0.20 | 0.26 |  | coactivation |
| 055 | D | 6.67 | 6.01 | 80.12 | 16.65 | 0 | 5.76 | -1.28 | 7.72 | | | 0.07 | | -0.30 | 0.60 | -0.98 | 0.14 | 0.14 |  | coactivation |
| 055 | E | 5.95 | 5.30 | 89.79 | 0 | 0 | 5.09 | 3.72 | -1.46 | | | 1.14 | | -0.52 | -0.37 | -0.03 | 1.06 | 1.30 | 5 | reciprocal |
| 056 | A | 22.98 | 19.25 | 82.34 | 14.74 | 0 | 26.2 | 19.93 | 11.95 | | | 0.25 | | -0.17 | -0.22 | 0.14 | 0.25 | 0.31 | 5 | reciprocal |
| 056 | B | 13.03 | 10.73 | 77.99 | 9.32 | 9.22 | 13.22 | 14.84 | 4.43 | | | -0.38 | | 0.62 | -0.20 | -0.13 | 0.18 | -0.09 |  | coactivation |
| 056 | C | 9.74 | 7.32 | 95.15 | 0 | 0 | 11.49 | 20.69 | 14.02 | | | -0.12 | | -0.87 | -0.11 | 0.09 | -0.28 | -0.26 |  | uncoupled |
| 056 | D | 6.21 | 6.25 | 45.49 | 32.01 | 16.01 | 7.7 | 16.35 | 9.70 | | | 0.61 | | -0.06 | -0.22 | 0.40 | 0.77 | 1.09 | 9 | uncoupled |
| 056 | E | 6.09 | 4.13 | 70.67 | 0 | 23.44 | 5.09 | 2.66 | 6.22 | | | 0.72 | | -1.01 | 0.48 | 0.48 | 0.42 | 0.80 |  | coactivation |
| 056 | F | 5.41 | 4.02 | 98.04 | 0 | 0 | 4.94 | 19.57 | 12.92 | | | -0.58 | | -0.15 | -0.94 | -0.03 | 0.43 | 0.10 |  | uncoupled |
| 056 | G | 5.11 | 4.07 | 98.35 | 0 | 0 | 4.98 | 14.49 | 18.98 | | | -0.83 | | 0.57 | -0.39 | 0.12 | 0.87 | 0.32 |  | coactivation |
| **Table S5.** Cluster-Level Results (Continued) | | | | | | | | | |  | | |  |  |  |  |  |  |  |  |
| **PP ID** | **Cluster** | **Percent Events** | **Number Events** | **Percent Sitting** | **Percent Standing** | **Percent Reclining** | **Percent Words** | **Mean Valence** | **Mean Arousal** | | | **IBI Change** | | **RSA Change** | **PEP Change** | **LVET Change** | **SV Change** | **CO Change** | **Common Pattern** | **Autonomic Control Mode** |
| 056 | H | 5.00 | 3.10 | 96.11 | 0 | 0 | 3.81 | 17.38 | 10.95 | | | -0.74 | | 0.16 | 0.11 | 1.41 | 0.59 | 0.05 |  | uncoupled |
| 057 | A | 27.81 | 32.13 | 69.3 | 18.35 | 5.97 | 26.12 | 5.00 | 16.62 | | | 0.87 | | -0.32 | -0.01 | 0.11 | 0.01 | 0.38 | 8 | uncoupled |
| 057 | B | 15.61 | 18.25 | 68.94 | 13.35 | 15.55 | 14.83 | 8.43 | 18.49 | | | -0.06 | | 0.04 | 0.30 | -0.23 | 0.12 | 0.06 |  | uncoupled |
| 057 | C | 10.71 | 12.45 | 79.76 | 0 | 8.03 | 11.74 | 7.94 | 18.16 | | | 1.49 | | 0.59 | -0.08 | -0.01 | 0.06 | 0.59 |  | uncoupled |
| 057 | D | 10.03 | 11.21 | 71.23 | 8.89 | 17.84 | 9.11 | 9.60 | 18.00 | | | -0.31 | | -0.22 | -0.01 | -1.17 | -0.59 | -0.62 | 3 | uncoupled |
| 058 | A | 20.05 | 16.41 | 97.76 | 0 | 0 | 23.44 | -2.05 | -11.94 | | | 0.77 | | -0.26 | 0.11 | 0.06 | -0.02 | 0.33 | 8 | uncoupled |
| 058 | B | 16.93 | 11.03 | 96.98 | 0 | 0 | 15.73 | -1.17 | -9.00 | | | 0.17 | | -0.96 | 0.07 | 0.00 | 0.53 | 0.51 | 6 | uncoupled |
| 058 | C | 9.67 | 10.08 | 85.56 | 9.91 | 0 | 14.38 | -3.72 | -16.19 | | | 0.84 | | 0.15 | -0.66 | -0.06 | 0.23 | 0.56 |  | uncoupled |
| 058 | D | 9.45 | 8.12 | 98.19 | 0 | 0 | 11.6 | -1.12 | 3.08 | | | -0.90 | | -0.43 | -0.36 | 0.27 | -0.19 | -0.56 |  | reciprocal |
| 058 | E | 7.25 | 5.17 | 96.78 | 0 | 0 | 7.37 | -12.35 | -21.32 | | | -0.09 | | 0.40 | 0.05 | 0.82 | 0.28 | 0.20 |  | uncoupled |
| 059 | A | 24.85 | 23.57 | 58.41 | 23.05 | 14.81 | 27.92 | 8.51 | -2.28 | | | 0.44 | | -0.07 | -0.14 | -0.10 | 0.05 | 0.27 |  | negligible change |
| 059 | B | 18.73 | 17.89 | 79.58 | 5.46 | 11.16 | 20.32 | 8.40 | -5.43 | | | -0.37 | | -0.37 | -0.20 | 0.16 | 0.12 | -0.16 |  | reciprocal |
| 059 | C | 8.30 | 6.22 | 61.16 | 32.1 | 0 | 8.2 | 8.10 | 0.09 | | | -0.80 | | 0.77 | -0.31 | -0.31 | 0.48 | 0.02 |  | coactivation |
| 061 | A | 50.64 | 34.96 | 68.11 | 2.86 | 27.92 | 47.17 | 3.93 | -6.80 | | | 0.25 | | -0.26 | -0.12 | 0.09 | 0.28 | 0.30 | 6 | uncoupled |
| 061 | B | 14.02 | 11.03 | 62.7 | 0 | 35.98 | 16.15 | 5.48 | -11.58 | | | -0.74 | | 0.14 | -0.09 | -0.29 | -0.59 | -0.60 |  | negligible change |
| 061 | C | 9.71 | 8.24 | 36.21 | 12.04 | 48.53 | 14.52 | 12.72 | -26.15 | | | -0.04 | | -0.19 | 1.03 | -0.40 | -0.27 | -0.26 |  | coactivation |
| 061 | D | 6.42 | 3.69 | 73.22 | 0 | 25.34 | 5.09 | 6.83 | -2.32 | | | -0.82 | | -0.75 | -0.22 | -0.18 | -0.67 | -0.80 |  | reciprocal |
| 062 | A | 29.17 | 23.22 | 71.43 | 12.49 | 8.61 | 30.16 | -5.67 | 8.24 | | | -0.66 | | 0.16 | -0.29 | -0.04 | -0.15 | -0.34 |  | coactivation |
| 062 | B | 25.78 | 25.56 | 62.84 | 19.7 | 15.48 | 35.38 | -1.71 | 12.53 | | | 0.25 | | -0.28 | 0.14 | -0.01 | 0.07 | 0.14 |  | uncoupled |
| 062 | C | 7.87 | 9.07 | 77.05 | 11.03 | 11.02 | 10.65 | -5.32 | -2.68 | | | 1.55 | | 0.52 | -0.06 | 0.77 | 0.16 | 0.64 | 1 | uncoupled |
| 063 | A | 19.38 | 23.80 | 49.6 | 19.18 | 17.89 | 19.57 | 9.91 | 11.35 | | | -0.06 | | 0.19 | -0.01 | 0.09 | -0.27 | -0.16 |  | uncoupled |
| 063 | B | 10.69 | 12.88 | 52.62 | 9.8 | 25.52 | 10.39 | 11.10 | 12.40 | | | -0.42 | | 0.03 | -0.59 | 0.02 | -0.03 | -0.17 |  | uncoupled |
| 063 | C | 9.57 | 12.14 | 77.71 | 16.4 | 0 | 10.86 | 13.05 | 12.08 | | | 0.28 | | -0.05 | 0.12 | -1.11 | -0.47 | -0.18 |  | negligible change |
| 063 | D | 9.19 | 10.41 | 57.37 | 18.44 | 18.98 | 9.2 | 13.14 | 16.42 | | | 0.97 | | 0.10 | 0.36 | 0.37 | 0.28 | 0.58 |  | uncoupled |
| 063 | E | 8.10 | 9.25 | 73.86 | 16.8 | 0 | 7.49 | 14.25 | 11.83 | | | -0.98 | | -0.53 | 0.00 | -0.74 | -0.42 | -0.66 | 3 | uncoupled |
| 063 | F | 5.74 | 8.71 | 58.38 | 30 | 5.46 | 7.04 | 11.09 | 15.49 | | | 0.56 | | -0.13 | -1.00 | 0.39 | 0.56 | 0.59 | 9 | uncoupled |
| 063 | G | 5.04 | 5.15 | 19.38 | 52.33 | 18.61 | 4.15 | 23.20 | 11.01 | | | 0.05 | | -0.21 | 0.70 | -0.66 | -1.17 | -0.70 |  | coactivation |
| 064 | A | 23.99 | 19.21 | 79.12 | 15.52 | 5.11 | 25.39 | -0.60 | -3.28 | | | 0.55 | | -0.05 | 0.28 | -0.02 | 0.16 | 0.46 |  | uncoupled |
| 064 | B | 12.40 | 9.19 | 93.81 | 0 | 0 | 12.52 | 3.98 | 5.34 | | | 0.66 | | -0.14 | 0.13 | 0.29 | 0.49 | 0.64 | 10 | negligible change |
| 064 | C | 11.38 | 8.78 | 74.72 | 0 | 22.41 | 9.63 | -0.70 | -3.22 | | | -1.13 | | 0.21 | -0.33 | 0.06 | -0.26 | -0.71 |  | coactivation |
| 064 | D | 7.28 | 5.70 | 87.74 | 0 | 0 | 6.51 | -0.75 | -2.05 | | | -0.05 | | 0.27 | 0.55 | -0.23 | -0.91 | -0.76 |  | reciprocal |
| 064 | E | 6.05 | 4.73 | 95.51 | 0 | 0 | 4.59 | -0.33 | -2.95 | | | 0.70 | | -0.41 | -0.44 | 0.55 | 0.06 | 0.41 |  | reciprocal |
| 064 | F | 5.14 | 7.16 | 97.8 | 0 | 0 | 10.87 | 6.34 | 1.06 | | | 1.22 | | -0.13 | 0.72 | 0.67 | 0.30 | 0.65 |  | uncoupled |
| 066 | A | 35.54 | 37.21 | 55.54 | 7.27 | 31.06 | 35.64 | 6.09 | -6.49 | | | 0.25 | | -0.31 | 0.05 | 0.02 | -0.18 | 0.02 |  | uncoupled |
| 066 | B | 12.33 | 12.34 | 84.52 | 0 | 11.41 | 11.37 | 0.23 | 1.86 | | | -0.32 | | -0.95 | 0.02 | -0.21 | 0.08 | -0.14 |  | uncoupled |
| 066 | C | 8.42 | 12.19 | 43.36 | 16.38 | 32.8 | 10.04 | -2.24 | -14.91 | | | 0.38 | | -0.29 | -0.63 | 0.29 | 0.38 | 0.51 | 4 | reciprocal |
| 066 | D | 5.73 | 6.51 | 73.66 | 13.76 | 0 | 7.72 | -3.17 | -5.64 | | | -0.96 | | 0.23 | -0.12 | -0.42 | -0.41 | -0.73 |  | uncoupled |
| 067 | A | 19.44 | 23.38 | 76.01 | 3.86 | 8.55 | 21.96 | 6.69 | 10.03 | | | 0.66 | | -1.13 | -0.19 | 0.12 | 0.65 | 0.84 | 5 | reciprocal |
| 067 | B | 19.25 | 19.82 | 92.61 | 0 | 0 | 19.65 | 9.13 | 11.80 | | | -0.24 | | -0.73 | -0.28 | -0.14 | -0.06 | -0.17 |  | reciprocal |
| 067 | C | 19.00 | 21.58 | 85.16 | 8.59 | 0 | 19.44 | 6.10 | 12.28 | | | 0.57 | | -0.40 | 0.09 | 0.60 | 0.35 | 0.56 | 7 | uncoupled |
| 067 | D | 6.39 | 6.38 | 61.03 | 29.71 | 0 | 6.62 | 5.15 | 0.15 | | | 0.55 | | -0.64 | 0.10 | -1.06 | -0.89 | -0.40 |  | uncoupled |
| **Table S5.** Cluster-Level Results (Continued) | | | | | | | | | | |  |  |  |  |  |  |  |  |  |  |
| **PP ID** | **Cluster** | **Percent Events** | **Number Events** | **Percent Sitting** | **Percent Standing** | **Percent Reclining** | **Percent Words** | **Mean Valence** | **Mean Arousal** | | | **IBI Change** | | **RSA Change** | **PEP Change** | **LVET Change** | **SV Change** | **CO Change** | **Common Pattern** | **Autonomic Control Mode** |
| 068 | A | 25.50 | 27.82 | 82.89 | 9.96 | 3.59 | 24.84 | 4.98 | -3.98 | | | -0.35 | | -0.36 | -0.26 | -0.01 | -0.12 | -0.29 |  | reciprocal |
| 068 | B | 20.96 | 22.97 | 81.97 | 8.61 | 4.29 | 20.51 | 9.36 | -0.42 | | | 0.90 | | -0.25 | 0.24 | 0.00 | 0.26 | 0.77 |  | coactivation |
| 068 | C | 10.81 | 12.29 | 98.5 | 0 | 0 | 10.97 | 6.12 | -2.15 | | | 0.71 | | 0.30 | -0.26 | 0.57 | 0.41 | 0.65 | 2 | coactivation |
| 068 | D | 7.76 | 8.02 | 99.75 | 0 | 0 | 7.15 | 11.37 | 6.37 | | | 0.34 | | 0.26 | -0.08 | -1.78 | -0.43 | -0.10 |  | uncoupled |

Note: A total of 250 clusters were identified across all 52 participants. Dark gray rows at top denote clusters from the six participants with fewer than 70 usable events (i.e., with physiological data devoid of major artifact); these participants were excluded from main analyses. Cells in the “Common Pattern” column are filled according to whether the cluster represents a pattern of physiological activity that occurred at least twice (light orange) or at least five times (medium orange). Clusters corresponding to the 10 most common patterns are further denoted by an identifying number.

**Participant-Level Comparisons across Clusters**

To contextualize comparisons of affective features (i.e., valence and arousal) across clusters, we also examined how clusters corresponded with three types of features: posture (i.e., weighted percentage of events that occurred while sitting, standing, or reclining); time of day (i.e., weighted percentage of events that occurred in the morning [before 12 pm], afternoon [12-5 pm], and evening [after 5 pm]); and type of activity (i.e., weighted percentage of events that occurred during the nine activities that participants most frequently endorsed during experience sampling [working, using computer, socializing, relaxing, eating, commuting, watching TV, on the phone, other]). We selected these features because they represent physical and psychological contexts that are known or likely to have bearing on peripheral physiological activity^31,32^.

We assessed the extent to which these features corresponded with the clustering solution discovered for each individual in our sample. We did this by conducting separate within-participant, across-clusters comparisons for each feature using a Kruskal-Wallis one-way ANOVA (as these variables were non-normally distributed). We quantified the influence of each feature on the clustering solution as the effect size of the Kruskal-Wallis test. We then compared the effect sizes for the different feature types: posture, time of day, type of activity, and affect. This allowed us to assess whether other physical and psychological contexts more strongly corresponded with the observed patterns of physiological activity (i.e., clusters) than affective features. Based on previous literature^31,32^, we predicted that physical contexts (i.e., posture, time of day) would have larger effect sizes than psychological contexts (i.e., affect, type of activity), indicating greater correspondence with patterns of physiological activity.

The results of all of these tests for each participant are reported in Table S6. We observed a larger number of significant Kruskal-Wallis tests (*H* values) for posture and time-of-day features than for affect and type of activity. For example, 10 (21.74%) of 46 participants had one or more clusters that significantly differed on the percentage of events while sitting; in contrast, only 2 (4.35% of) participants had one or more clusters that significantly differed on valence. We compared the average effect sizes (*ω^2^*)^33^ of the Kruskal-Wallis tests and found a significant effect of feature type, *F*(3,204) = 2.84, *p* < .05, two-tailed. Pairwise comparisons revealed that this effect was driven by posture features, which had larger effect sizes than affective and type-of-activity features, both *p*s < .05, Bonferroni corrected, but did not significantly differ from time-of-day features. As such, these results are consistent with our predictions.


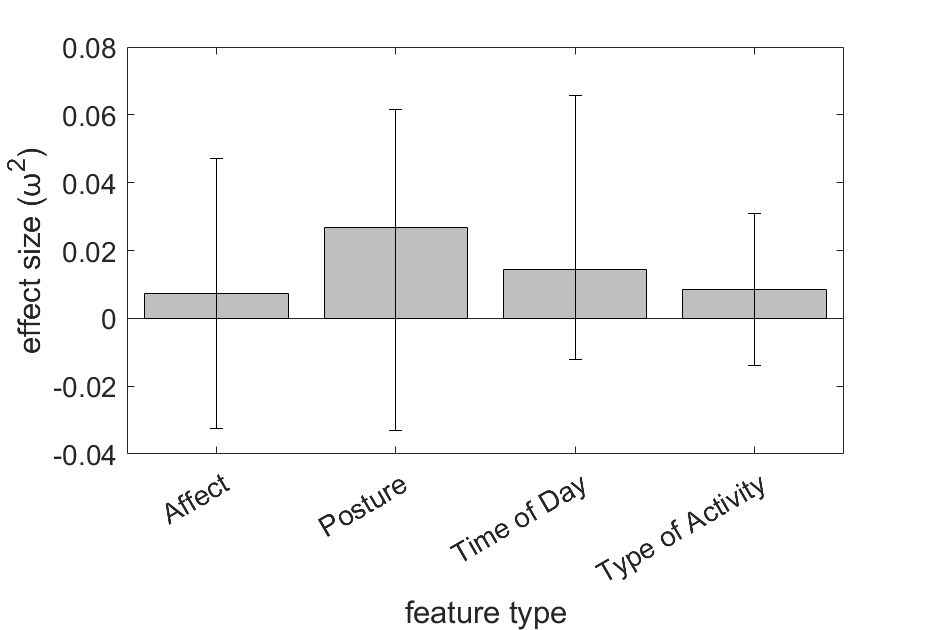


**Figure S3.** Comparison of effect size (*ω^2^*) by feature type in participant-level comparisons across clusters. Error bars represent standard deviations.

**Table S6.** Summary of Participant-Level Kruskal-Wallis Tests for Differences across Clusters

| **PP ID** | **df** | **Valence** | **Arousal** | **Sitting** | **Standing** | **Reclining** | **Morning** | **Afternoon** | **Evening** | **Working** | **Computer** | **Socializing** | **Relaxing** | **Eating** | **TV** | **Phone** | **Commuting** | **Other** |
| --- | --- | --- | --- | --- | --- | --- | --- | --- | --- | --- | --- | --- | --- | --- | --- | --- | --- | --- |
| 010 | 6 | *7.45* | *7.45* | *10.66* | *8.31* | -- | *7.28* | *5.51* | ***20.97*** | *5.56* | *7.49* | *8.33* | *6.50* | *6.50* | *4.89* | -- | -- | *7.34* |
| 025 | 3 | 1.84 | 0.70 | *1.87* | *6.65* | 1.31 | *3.40* | *3.02* | 1.50 | 1.34 | -- | *2.85* | 2.09 | *2.71* | -- | -- | 0.41 | *2.71* |
| 026 | 4 | 4.73 | 7.12 | *1.21* | *6.50* | *8.13* | *2.71* | **12.41** | *2.63* | *6.32* | *3.57* | *3.57* | -- | *6.02* | 1.91 | -- | ***15.00*** | -- |
| 038 | 5 | 5.24 | 2.42 | *5.51* | *7.92* | ***11.50*** | *6.44* | *3.20* | *--* | 3.59 | *5.87* | -- | -- | 1.67 | -- | *3.11* | ***21.32*** | *2.97* |
| 060 | 2 | 0.05 | 1.90 | 0.98 | 0.41 | *1.90* | 0.43 | 0.15 | 0.63 | -- | -- | 1.81 | *1.43* | -- | *3.00* | *3.00* | -- | 0.80 |
| 065 | 5 | 4.27 | ***11.80*** | 10.63 | ***13.83*** | 3.43 | *4.74* | *7.27* | *6.04* | *5.83* | *6.70* | *5.90* | -- | -- | *5.69* | *2.89* | ***13.33*** | *5.75* |
| 002 | 6 | 3.81 | *3.03* | ***13.43*** | *7.93* | *3.71* | 7.45 | 6.94 | *5.52* | *4.75* | *7.57* | *9.71* | -- | *9.38* | -- | -- | -- | 2.26 |
| 003 | 4 | 3.63 | 2.63 | 8.00 | *4.71* | *3.10* | 1.93 | 7.55 | *2.83* | 3.44 | *2.37* | *4.28* | -- | 2.38 | -- | -- | *4.17* | *4.64* |
| 006 | 5 | 2.13 | 6.25 | *7.15* | ***12.69*** | 2.36 | ***11.74*** | *11.01* | *--* | *8.40* | *5.03* | ***27.35*** | *5.84* | *4.92* | -- | -- | -- | 5.27 |
| 007 | 2 | 3.44 | *0.02* | *5.96* | *1.29* | -- | *2.89* | ***11.37*** | 0.73 | *1.27* | -- | 1.38 | 0.34 | *2.03* | 0.75 | -- | -- | -- |
| 009 | 5 | 4.62 | 10.60 | 8.12 | *5.61* | *5.76* | 6.85 | 5.11 | *7.88* | 4.95 | *6.10* | *5.55* | -- | *5.18* | *3.18* | -- | *6.76* | *2.44* |
| 011 | 2 | 3.34 | 4.45 | **6.65** | 0.37 | 1.29 | 1.02 | 0.82 | 0.37 | *2.48* | 0.40 | ***6.94*** | -- | *1.58* | *4.14* | -- | 0.37 | *4.55* |
| 012 | 2 | *3.60* | **9.64** | *4.71* | ***11.26*** | *1.35* | ***8.83*** | ***8.29*** | 0.80 | 0.12 | 0.80 | -- | *1.87* | 0.66 | *1.55* | -- | ***7.88*** | 1.58 |
| 013 | 4 | 1.63 | 6.13 | 9.11 | *2.92* | 1.68 | 1.59 | 1.13 | *2.59* | 2.69 | 2.43 | *0.79* | *2.37* | -- | -- | ***11.00*** | -- | *3.53* |
| 014 | 4 | 7.01 | *5.75* | 2.22 | *3.62* | *3.01* | 5.28 | *5.79* | *4.18* | 2.44 | *3.38* | *8.56* | *5.33* | *5.23* | *3.49* | *4.18* | 1.15 | -- |
| 015 | 4 | 2.51 | 1.32 | 3.74 | *3.99* | *3.83* | *5.78* | *8.62* | *3.14* | ***11.17*** | *2.83* | *7.33* | *6.71* | *3.24* | 1.76 | -- | -- | *7.64* |
| 017 | 4 | *1.80* | *9.46* | *6.07* | *5.17* | *5.48* | 0.80 | 2.28 | *7.93* | *3.70* | 2.18 | *2.09* | *2.44* | *5.00* | -- | *2.67* | *2.67* | 3.15 |
| 018 | 5 | 6.09 | 4.74 | ***11.98*** | *4.15* | 1.58 | *3.25* | 4.06 | 1.40 | *6.81* | *4.63* | *7.41* | 1.95 | ***17.12*** | -- | -- | *3.72* | 3.64 |
| 019 | 4 | 4.62 | 3.77 | 1.73 | *4.94* | 1.95 | *7.02* | *8.12* | *3.66* | -- | 2.40 | *4.26* | *3.18* | *6.41* | -- | *6.89* | *3.18* | 1.83 |
| 021 | 3 | *3.29* | ***9.98*** | 1.81 | *2.07* | *2.99* | **17.37** | 4.12 | ***9.27*** | ***10.30*** | 0.99 | *3.62* | 1.76 | 2.27 | 0.98 | *3.00* | *4.95* | ***12.82*** |
| 022 | 3 | **14.69** | 1.53 | *6.84* | 1.65 | *4.69* | *3.57* | ***16.40*** | *1.72* | -- | 6.67 | ***13.36*** | *2.48* | 1.91 | 1.74 | -- | *3.18* | *3.53* |
| 023 | 3 | 0.37 | 2.62 | 0.54 | 0.16 | 1.70 | 2.40 | 1.96 | *5.33* | 1.31 | 2.60 | 1.96 | 1.38 | *2.57* | -- | *2.31* | -- | *2.80* |
| 024 | 3 | *4.12* | 5.16 | ***14.70*** | ***8.33*** | *5.06* | *2.36* | **8.46** | 1.51 | 1.10 | *2.78* | 1.80 | ***20.37*** | 1.39 | *4.94* | 1.41 | -- | 4.00 |
| 027 | 5 | 7.46 | *5.20* | *9.52* | *3.35* | *5.45* | *5.67* | *6.45* | *6.72* | *5.10* | -- | *4.46* | ***11.52*** | *10.99* | *5.34* | 2.54 | *4.29* | 2.04 |
| 028 | 3 | 3.64 | *6.75* | *6.12* | *5.27* | *2.39* | 1.71 | 1.25 | ***9.14*** | *4.78* | 0.60 | 0.82 | 3.10 | 0.86 | *7.44* | *3.91* | -- | *2.56* |
| 029 | 5 | **13.59** | **12.78** | ***15.38*** | *4.64* | -- | 0.67 | **13.08** | *4.58* | 7.53 | *3.08* | *6.13* | -- | *2.80* | -- | *5.55* | -- | 1.94 |
| 032 | 2 | 0.74 | 1.17 | *2.73* | 0.65 | ***8.31*** | 0.05 | 3.55 | *--* | 2.18 | 1.49 | 1.21 | -- | 1.15 | -- | -- | -- | *2.10* |
| 033 | 4 | 2.72 | 5.80 | 2.98 | *9.40* | ***10.98*** | 3.35 | 2.21 | *--* | *4.71* | *2.36* | *4.31* | ***11.04*** | *2.38* | -- | ***10.87*** | -- | *4.46* |
| 034 | 3 | 0.75 | 2.70 | 5.63 | *2.18* | *5.94* | ***8.02*** | *4.56* | *1.92* | 3.54 | *1.58* | 1.53 | 1.07 | *5.62* | *4.44* | -- | *2.64* | ***8.50*** |
| 036 | 2 | 0.90 | 0.79 | 3.66 | *1.48* | 0.97 | 3.22 | 0.47 | *--* | ***6.23*** | 0.97 | -- | -- | *5.92* | -- | -- | -- | 0.48 |
| 037 | 5 | 5.52 | 2.94 | 4.79 | *3.30* | ***11.59*** | 1.97 | 3.23 | *2.87* | -- | *6.08* | -- | *4.92* | *8.25* | *9.32* | *7.29* | *8.79* | 1.13 |
| 040 | 6 | 7.76 | 7.91 | *10.87* | *8.87* | *7.35* | 2.48 | 1.92 | *--* | 2.00 | -- | -- | -- | *5.24* | -- | *6.22* | 4.72 | *2.98* |
| 043 | 4 | 2.06 | *3.51* | *6.95* | -- | -- | ***10.93*** | ***15.28*** | *--* | 2.57 | 0.85 | *3.49* | -- | *6.67* | -- | *3.15* | *6.67* | *6.67* |
| 045 | 4 | 2.56 | 3.55 | *3.19* | -- | *3.88* | 2.13 | 2.90 | *4.22* | *3.70* | *5.45* | *3.27* | 1.88 | *2.36* | -- | *3.27* | -- | -- |
| 046 | 4 | 4.06 | 2.99 | *4.06* | *7.10* | *4.50* | 1.83 | 3.14 | *4.92* | *2.85* | 1.46 | *2.40* | 1.68 | *6.09* | *2.78* | 1.63 | -- | ***10.94*** |
| 047 | 2 | 1.64 | *1.82* | *0.69* | *1.40* | *2.10* | 0.34 | 1.29 | *2.69* | 4.26 | -- | -- | -- | *2.96* | -- | -- | -- | *2.61* |

**Table S6.** Summary of Participant-Level Kruskal-Wallis Tests for Differences across Clusters (Continued)

| **PP ID** | **df** | **Valence** | **Arousal** | **Sitting** | **Standing** | **Reclining** | **Morning** | **Afternoon** | **Evening** | **Working** | **Computer** | **Socializing** | **Relaxing** | **Eating** | **TV** | **Phone** | **Commuting** | **Other** |
| --- | --- | --- | --- | --- | --- | --- | --- | --- | --- | --- | --- | --- | --- | --- | --- | --- | --- | --- |
| 049 | 4 | 4.87 | 3.54 | 8.87 | 5.14 | *2.38* | *6.54* | 2.74 | *5.23* | -- | 2.83 | *1.48* | *7.01* | 2.50 | *3.87* | -- | -- | 1.46 |
| 050 | 4 | 4.68 | 4.28 | *7.17* | *6.15* | *4.68* | *4.75* | 5.74 | *5.64* | 4.17 | -- | *8.00* | 0.36 | *3.45* | 1.60 | -- | 1.20 | 1.28 |
| 051 | 5 | 3.99 | *8.76* | 3.46 | *5.16* | *3.90* | 4.30 | 5.78 | *6.68* | ***12.25*** | *4.29* | *4.85* | ***11.87*** | *2.61* | *3.54* | 1.53 | *5.51* | *6.94* |
| 054 | 3 | 3.50 | 2.70 | 7.08 | ***8.58*** | *5.82* | *2.51* | ***10.46*** | *2.90* | *3.52* | *3.57* | *4.20* | 0.87 | *3.59* | -- | 1.02 | *2.94* | *4.06* |
| 055 | 4 | 1.18 | 2.56 | 4.67 | *3.49* | *2.31* | *5.14* | 6.98 | *2.96* | 3.84 | -- | *4.55* | *4.65* | -- | -- | ***11.20*** | -- | 0.68 |
| 056 | 7 | 8.55 | 6.87 | *10.82* | *6.59* | *7.12* | *3.39* | *12.37* | *5.04* | *11.20* | *8.52* | *3.65* | *4.31* | *6.19* | *4.84* | 1.95 | *12.29* | *7.67* |
| 057 | 3 | 2.11 | 2.88 | ***10.03*** | *2.77* | 1.81 | 4.05 | 1.75 | *5.73* | *5.73* | -- | *2.66* | 1.31 | *2.70* | -- | -- | -- | **18.33** |
| 058 | 4 | 1.00 | 5.87 | ***12.14*** | *4.10* | -- | 2.07 | 1.90 | 2.00 | *4.12* | *5.13* | -- | *3.81* | -- | -- | *4.10* | -- | 8.51 |
| 059 | 2 | 0.35 | 1.39 | *1.03* | *3.77* | 1.13 | 1.03 | 1.65 | *1.60* | *3.40* | *1.98* | ***13.27*** | 1.00 | -- | *2.01* | ***6.02*** | 1.00 | 3.02 |
| 061 | 3 | 5.64 | **9.78** | 1.79 | *2.40* | 3.40 | 0.66 | 0.77 | ***7.87*** | *3.99* | *6.14* | *2.58* | *3.93* | -- | -- | -- | -- | ***8.12*** |
| 062 | 2 | 2.56 | 2.70 | **6.43** | 0.58 | 0.22 | 0.54 | ***6.51*** | ***7.24*** | -- | 0.07 | 1.15 | 0.41 | 1.15 | -- | 2.42 | -- | 0.81 |
| 063 | 6 | 8.35 | 6.46 | *4.19* | 5.07 | *7.34* | *12.16* | 5.55 | *4.51* | ***15.83*** | 9.30 | *4.01* | 2.48 | *12.33* | *8.22* | *4.16* | -- | 1.74 |
| 064 | 5 | 5.23 | *2.77* | ***20.25*** | *5.45* | *6.29* | 1.86 | 10.17 | *3.90* | *3.96* | *3.64* | *4.84* | *3.28* | 2.54 | *6.61* | *3.46* | -- | *6.69* |
| 066 | 3 | 1.12 | 3.42 | *3.14* | *2.72* | *5.01* | 0.59 | 1.11 | *--* | ***8.91*** | *1.91* | 1.72 | *4.28* | *2.71* | *2.62* | 1.84 | -- | 1.42 |
| 067 | 3 | 1.40 | 3.34 | *0.79* | ***8.55*** | *4.32* | ***9.05*** | *0.47* | 1.29 | 1.63 | 1.79 | 2.12 | *3.23* | -- | *2.43* | *3.06* | -- | *1.77* |
| 068 | 3 | 2.34 | 0.41 | ***16.29*** | *2.25* | 0.88 | *2.81* | 7.26 | *4.31* | 1.41 | -- | 0.89 | 0.45 | ***10.25*** | *2.26* | -- | 1.54 | 0.72 |
| *% significant* | | 4.35% | 8.70% | 21.74% | 10.87% | 6.52% | 13.04% | 17.39% | 8.70% | 13.04% | 0.00% | 8.70% | 8.70% | 4.35% | 0.00% | 8.70% | 2.17% | 10.87% |
| *% unequal var.* | | 8.70% | 23.91% | 58.70% | 80.43% | 65.22% | 41.30% | 30.43% | 69.57% | 52.17% | 45.65% | 60.87% | 47.83% | 60.87% | 41.30% | 41.30% | 28.26% | 47.83% |
| *% significant* | | 3.85% | 9.62% | 19.23% | 11.54% | 7.69% | 11.54% | 17.31% | 9.62% | 11.54% | 0.00% | 7.69% | 7.69% | 3.85% | 0.00% | 7.69% | 7.69% | 9.62% |
| *% unequal var.* | | 9.62% | 25.00% | 59.62% | 80.77% | 63.46% | 46.15% | 34.62% | 67.31% | 51.92% | 48.08% | 61.54% | 44.23% | 63.46% | 42.31% | 42.31% | 34.62% | 50.00% |

Note: Dark gray rows at top denote clusters from the six participants with fewer than 70 usable events (i.e., with physiological data devoid of major artifact); these participants were excluded from main analyses. **bold** Kruskal-Wallis (*H*) significant at p < .05; *italics* Levene (*F*) test for equality of variances significant at p < .05; -- Unable to assess because there were no events corresponding with the given physiological or psychological feature

**Table S7.** All Repeated Patterns of Change in Physiological Activity

|  | **RSA** | **IBI** | **PEP** | **LVET** | **SV** | **CO** | **Freq. Included PPs** | **Rel. Freq. Included PPs** | **Freq. Excluded PPs** | **Rel. Freq. All PPs** |
| --- | --- | --- | --- | --- | --- | --- | --- | --- | --- | --- |
| 1 | + | + | NC | + | + | + | 9 | 4.11% | 0 | 3.60% |
| 2 | + | + | - | + | + | + | 8 | 3.65% | 1 | 3.60% |
| 3 | - | - | NC | - | - | - | 7 | 3.20% | 0 | 2.80% |
| 4 | - | + | - | + | + | + | 7 | 3.20% | 0 | 2.80% |
| 5 | - | + | - | NC | + | + | 7 | 3.20% | 0 | 2.80% |
| 6 | - | + | NC | NC | + | + | 7 | 3.20% | 0 | 2.80% |
| 7 | - | + | NC | + | + | + | 5 | 2.28% | 0 | 2.00% |
| 8 | - | + | NC | NC | NC | + | 5 | 2.28% | 0 | 2.00% |
| 9 | NC | + | - | + | + | + | 5 | 2.28% | 0 | 2.00% |
| 10 | NC | + | NC | + | + | + | 5 | 2.28% | 0 | 2.00% |
| 11 | - | - | - | NC | NC | - | 4 | 1.83% | 0 | 1.60% |
| 12 | - | - | + | - | - | - | 2 | 0.91% | 2 | 1.60% |
| 13 | - | NC | NC | NC | NC | NC | 4 | 1.83% | 0 | 1.60% |
| 14 | NC | NC | NC | NC | NC | NC | 4 | 1.83% | 0 | 1.60% |
| 15 | - | + | + | + | + | + | 3 | 1.37% | 0 | 1.20% |
| 16 | - | + | NC | - | - | - | 3 | 1.37% | 0 | 1.20% |
| 17 | + | - | + | - | - | - | 2 | 0.91% | 1 | 1.20% |
| 18 | + | - | NC | - | - | - | 3 | 1.37% | 0 | 1.20% |
| 19 | + | + | NC | NC | NC | + | 3 | 1.37% | 0 | 1.20% |
| 20 | NC | - | NC | - | - | - | 3 | 1.37% | 0 | 1.20% |
| 21 | NC | + | + | + | + | + | 3 | 1.37% | 0 | 1.20% |
| 22 | NC | + | + | NC | + | + | 3 | 1.37% | 0 | 1.20% |
| 23 | - | - | - | - | - | - | 1 | 0.46% | 1 | 0.80% |
| 24 | - | - | - | + | + | + | 2 | 0.91% | 0 | 0.80% |
| 25 | - | - | - | + | + | NC | 2 | 0.91% | 0 | 0.80% |
| 26 | - | - | + | NC | - | - | 0 | 0.00% | 2 | 0.80% |
| 27 | - | + | - | + | NC | + | 2 | 0.91% | 0 | 0.80% |
| 28 | - | + | + | - | - | NC | 2 | 0.91% | 0 | 0.80% |
| 29 | - | + | NC | - | NC | + | 2 | 0.91% | 0 | 0.80% |
| 30 | - | + | NC | - | NC | + | 0 | 0.00% | 2 | 0.80% |
| 31 | - | NC | + | - | - | - | 2 | 0.91% | 0 | 0.80% |
| 32 | - | NC | NC | NC | - | - | 2 | 0.91% | 0 | 0.80% |
| 33 | - | NC | - | NC | NC | NC | 0 | 0.00% | 2 | 0.80% |
| 34 | - | NC | NC | + | + | NC | 0 | 0.00% | 2 | 0.80% |
| 35 | + | - | - | - | + | NC | 2 | 0.91% | 0 | 0.80% |
| 36 | + | - | - | - | NC | - | 2 | 0.91% | 0 | 0.80% |
| 37 | + | - | - | + | + | + | 2 | 0.91% | 0 | 0.80% |
| 38 | + | - | + | NC | NC | - | 2 | 0.91% | 0 | 0.80% |
| 39 | + | - | NC | + | + | NC | 2 | 0.91% | 0 | 0.80% |
| 40 | + | + | - | + | - | NC | 0 | 0.00% | 2 | 0.80% |
| 41 | + | + | NC | + | NC | + | 2 | 0.91% | 0 | 0.80% |
| 42 | + | + | NC | NC | + | + | 2 | 0.91% | 0 | 0.80% |
| 43 | + | + | NC | - | - | - | 0 | 0.00% | 2 | 0.80% |
| 44 | + | NC | NC | NC | - | - | 2 | 0.91% | 0 | 0.80% |
| 45 | + | NC | - | NC | - | - | 0 | 0.00% | 2 | 0.80% |
| 46 | NC | - | - | NC | NC | - | 2 | 0.91% | 0 | 0.80% |
| 47 | NC | - | + | - | - | - | 2 | 0.91% | 0 | 0.80% |
| 48 | NC | + | + | + | - | NC | 1 | 0.46% | 1 | 0.80% |
| 49 | NC | + | + | NC | NC | + | 2 | 0.91% | 0 | 0.80% |
| 50 | NC | + | NC | NC | + | + | 2 | 0.91% | 0 | 0.80% |
| 51 | NC | NC | NC | - | - | - | 2 | 0.91% | 0 | 0.80% |
| 52 | NC | - | NC | - | NC | NC | 0 | 0.00% | 2 | 0.80% |

Note: A total of 52 repeated patterns accounted for 166 (66.40%) of the 250 clusters identified for the full sample of 52 participants. Of these patterns, the 10 most common (light gray rows at top) accounted for 66 (26.40%) of clusters. + increase; - decrease; nc negligible change on the given physiological feature


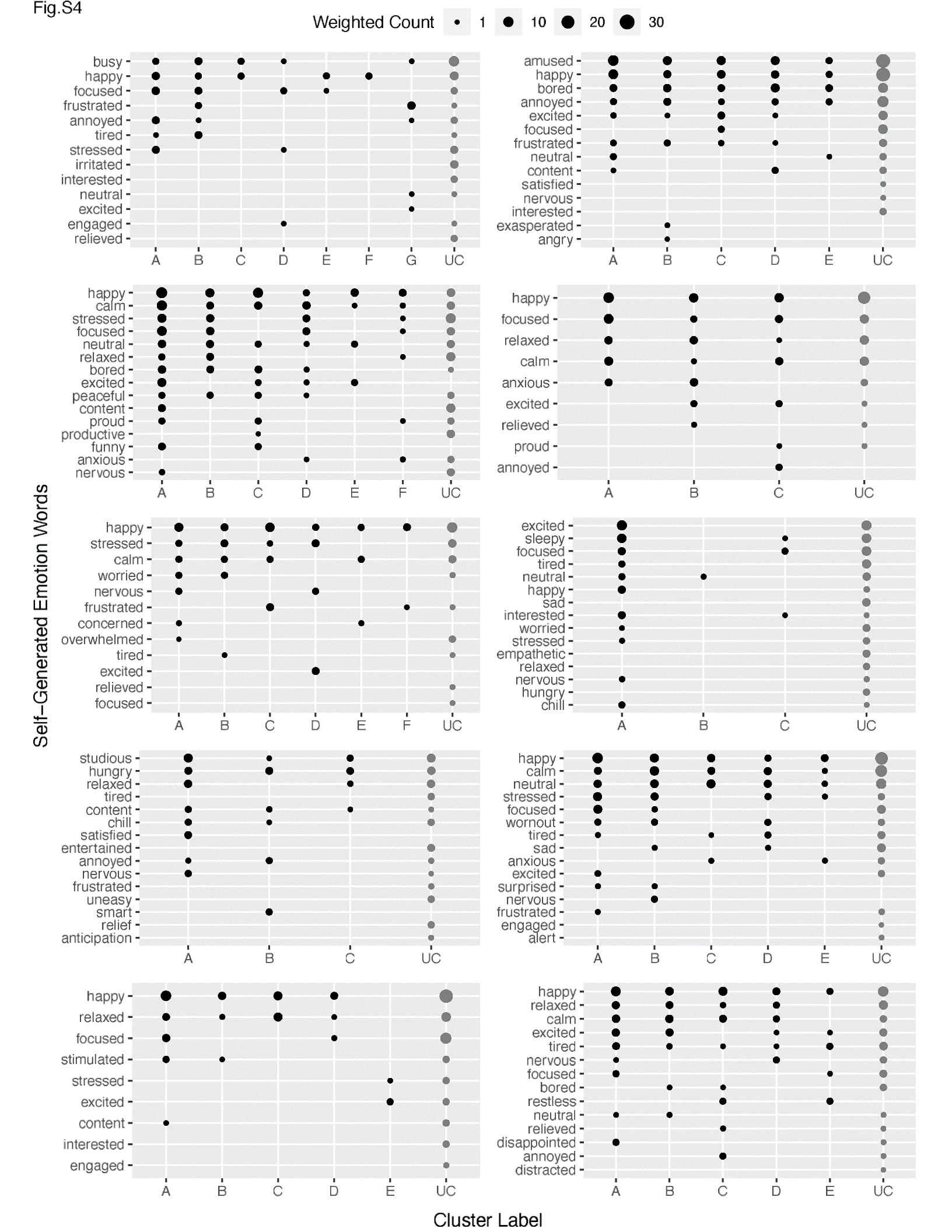


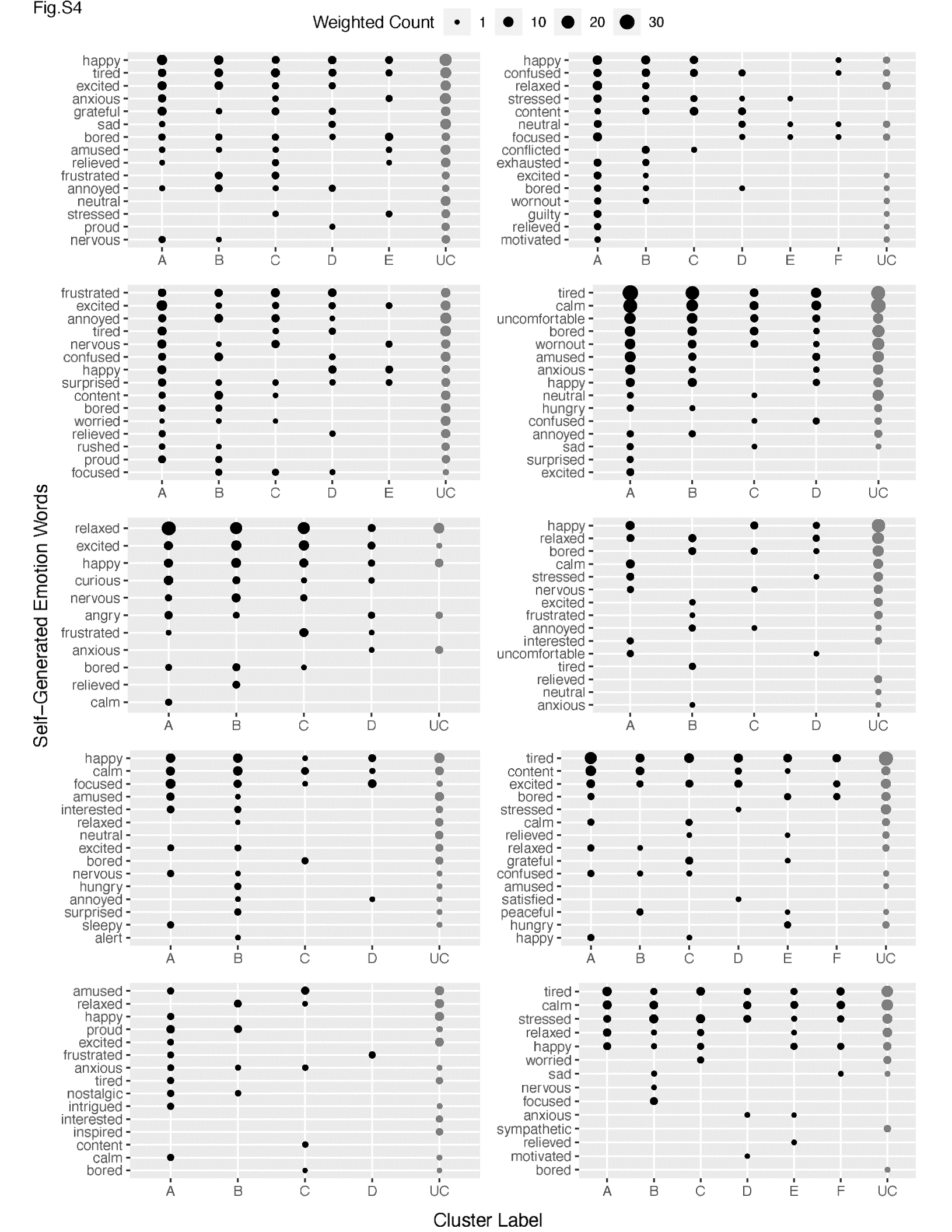


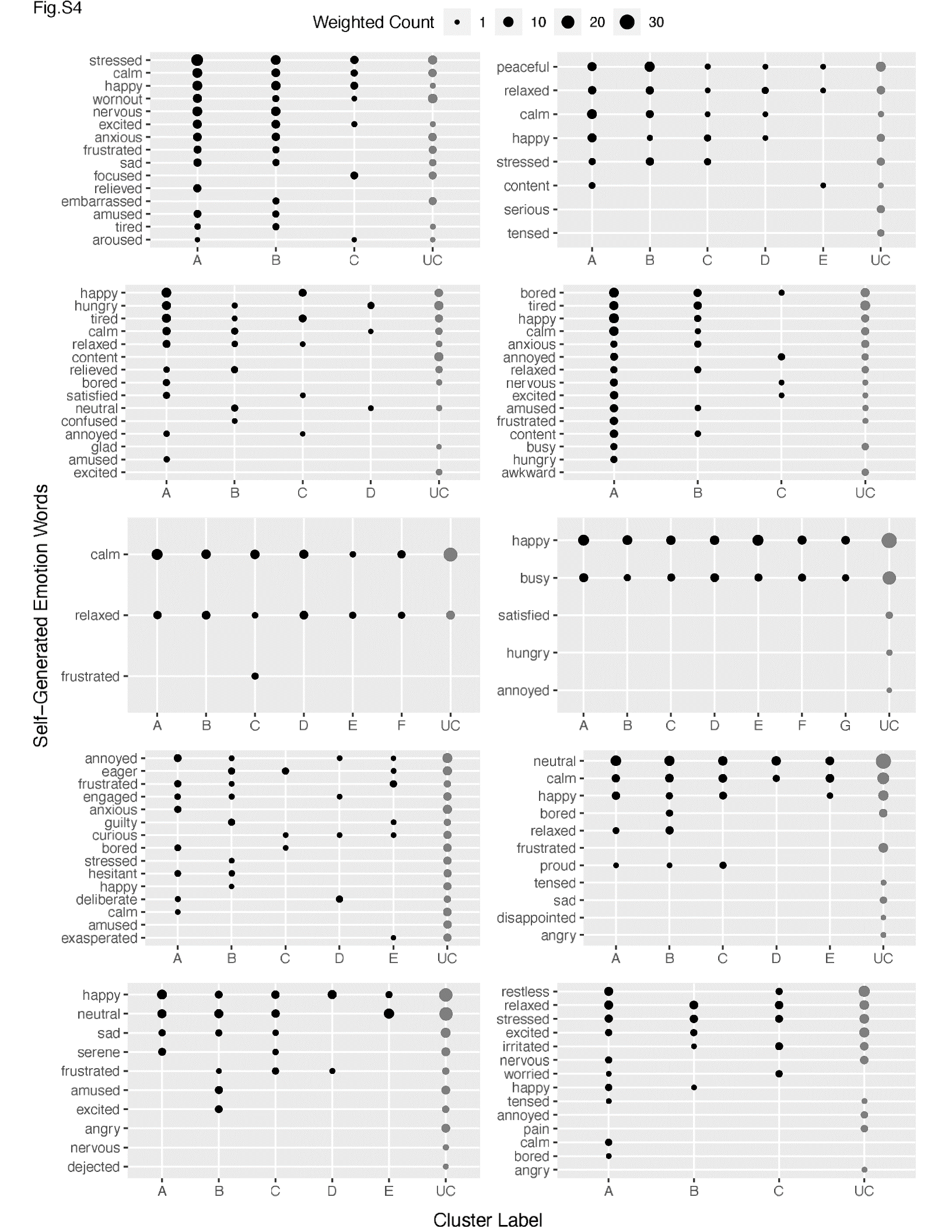


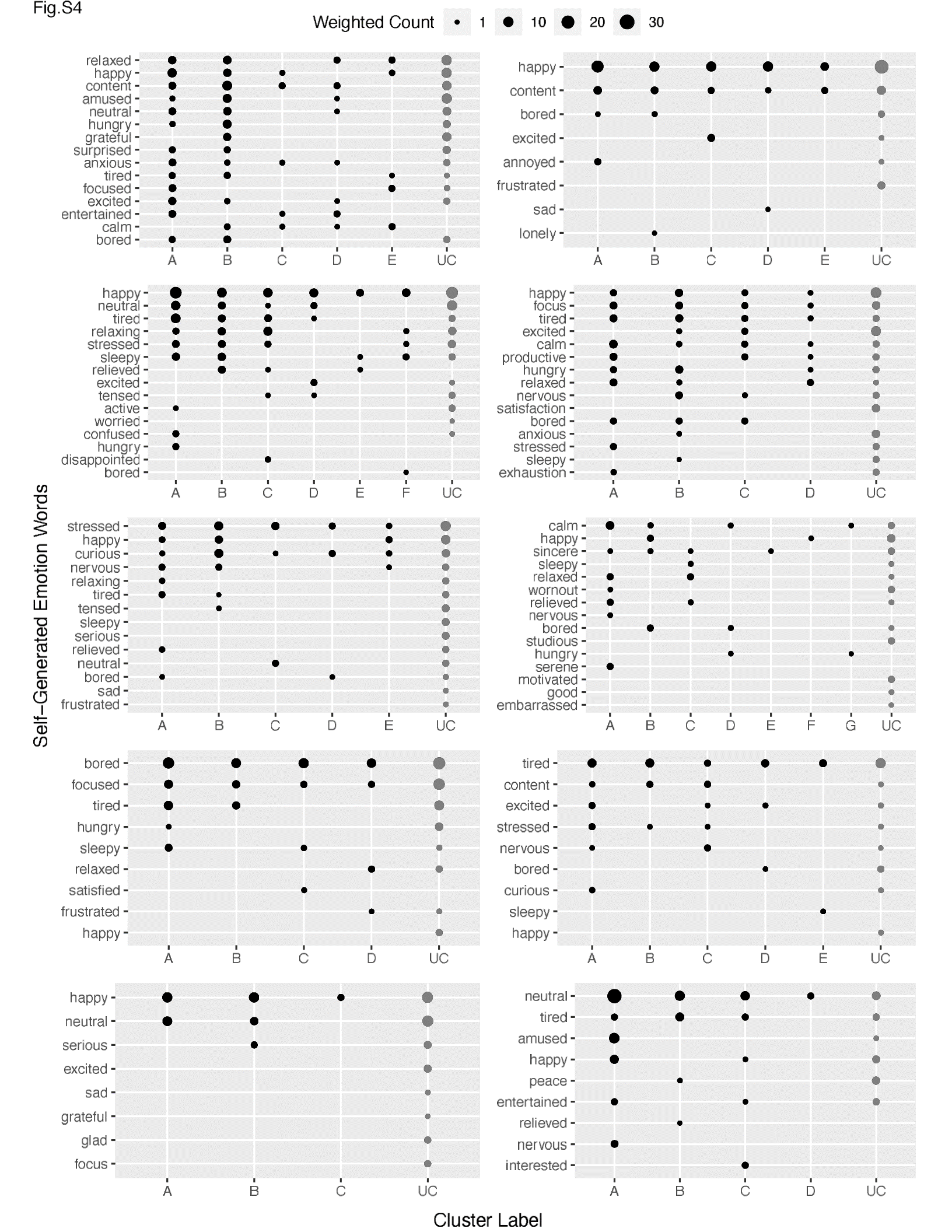


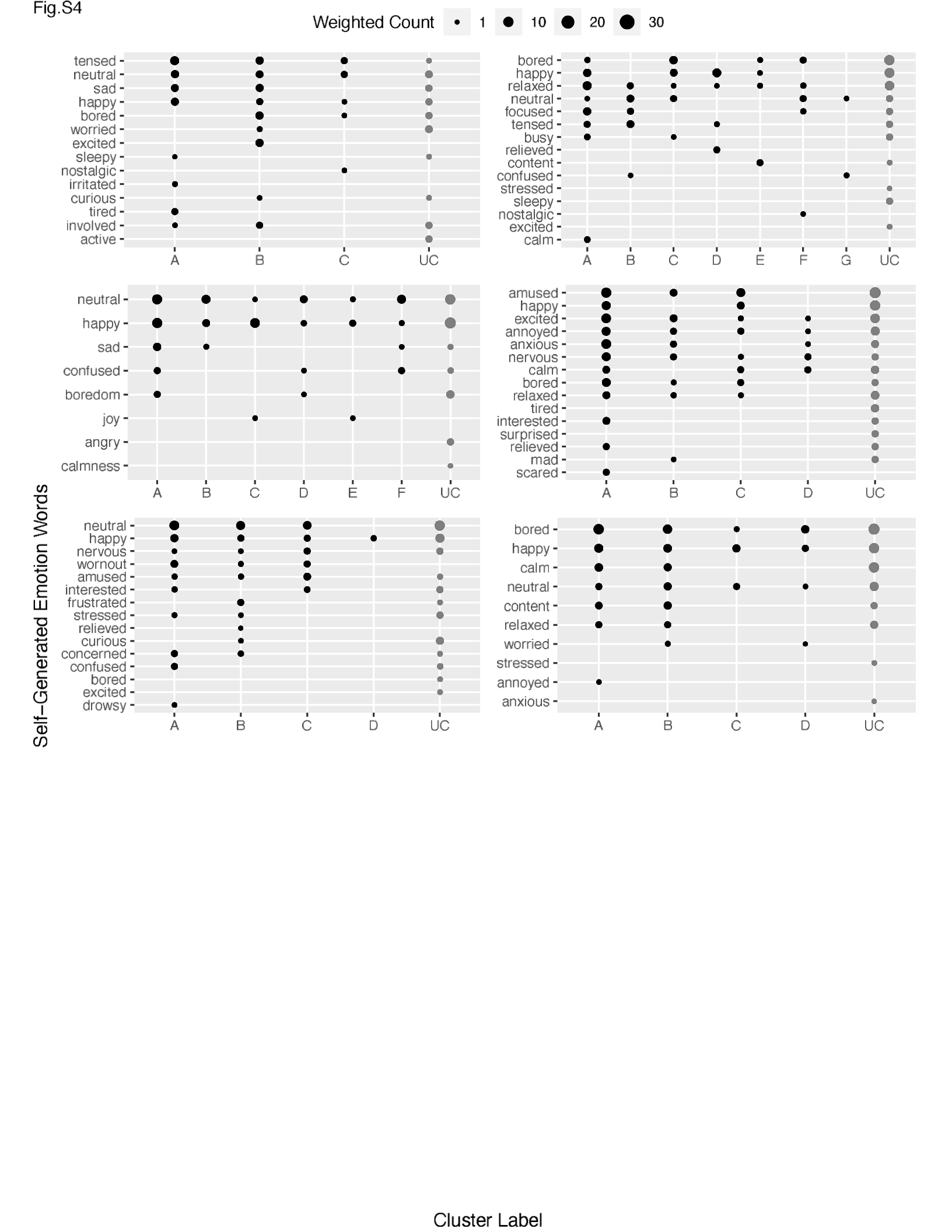

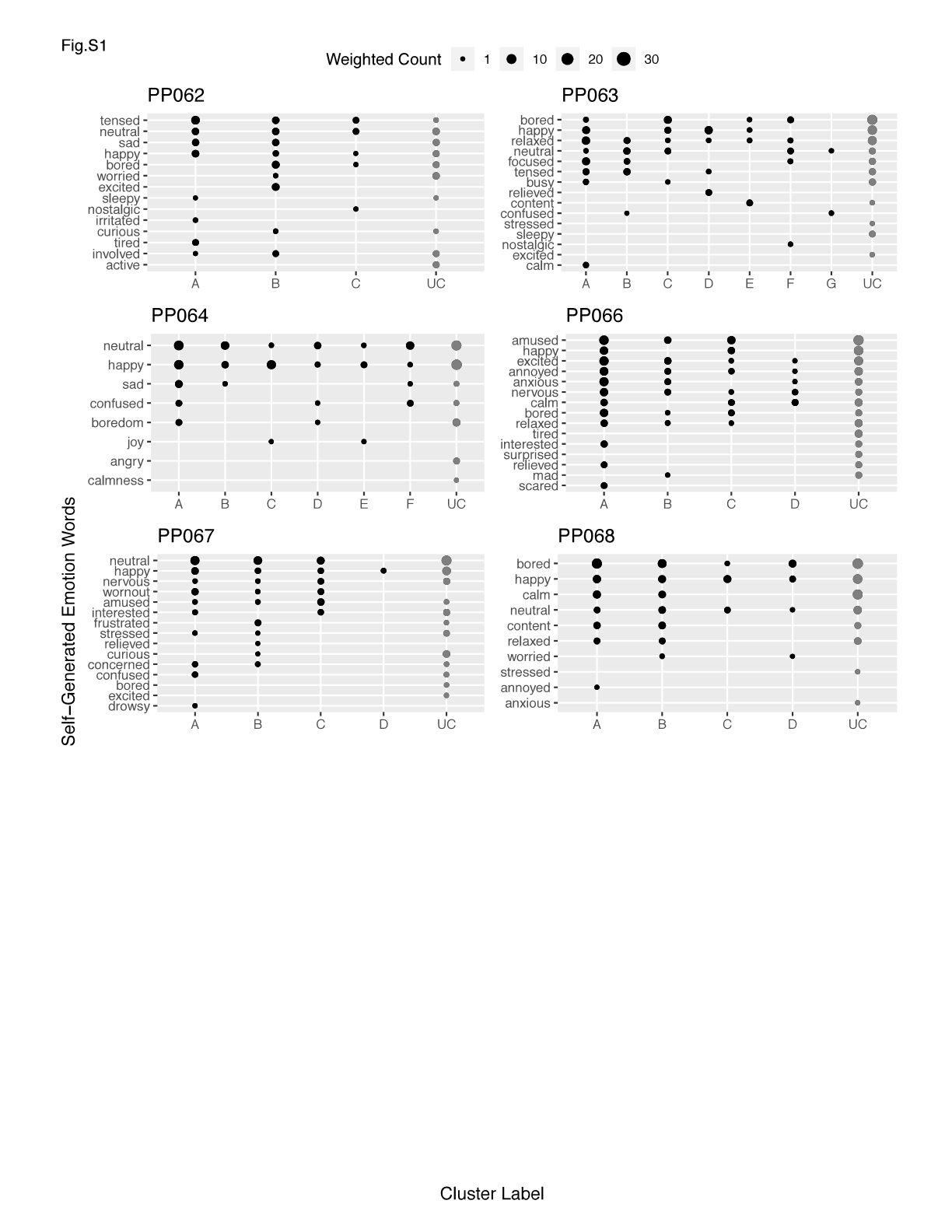

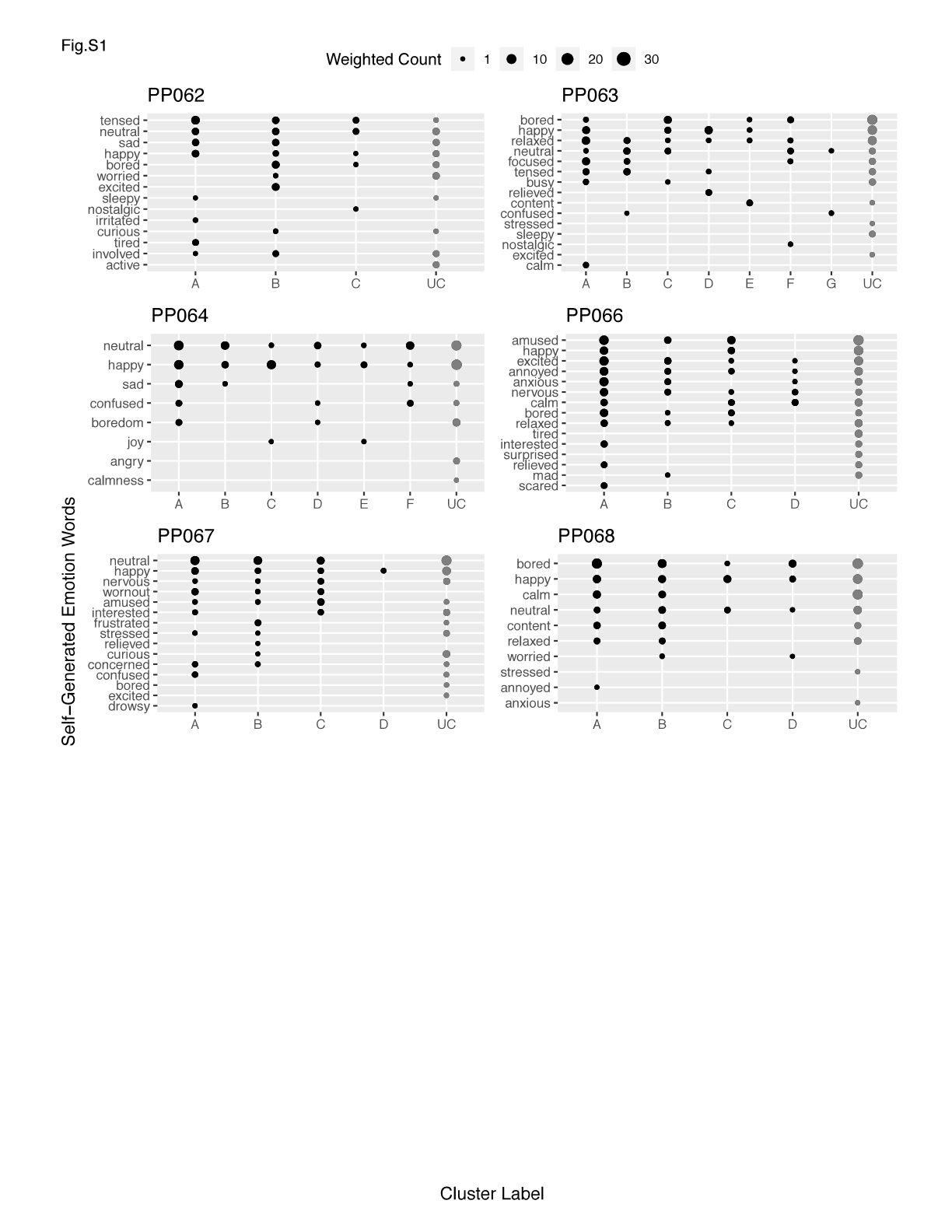

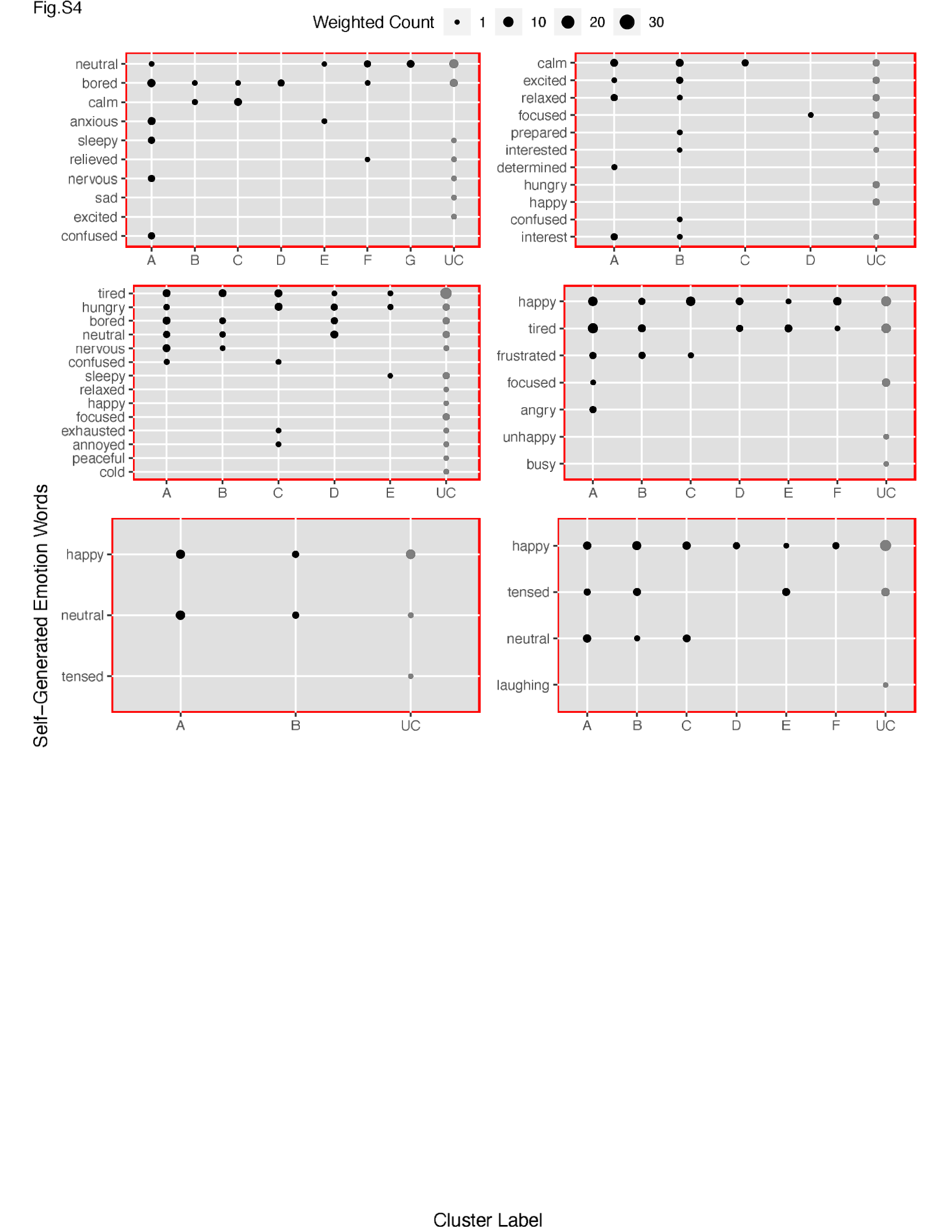

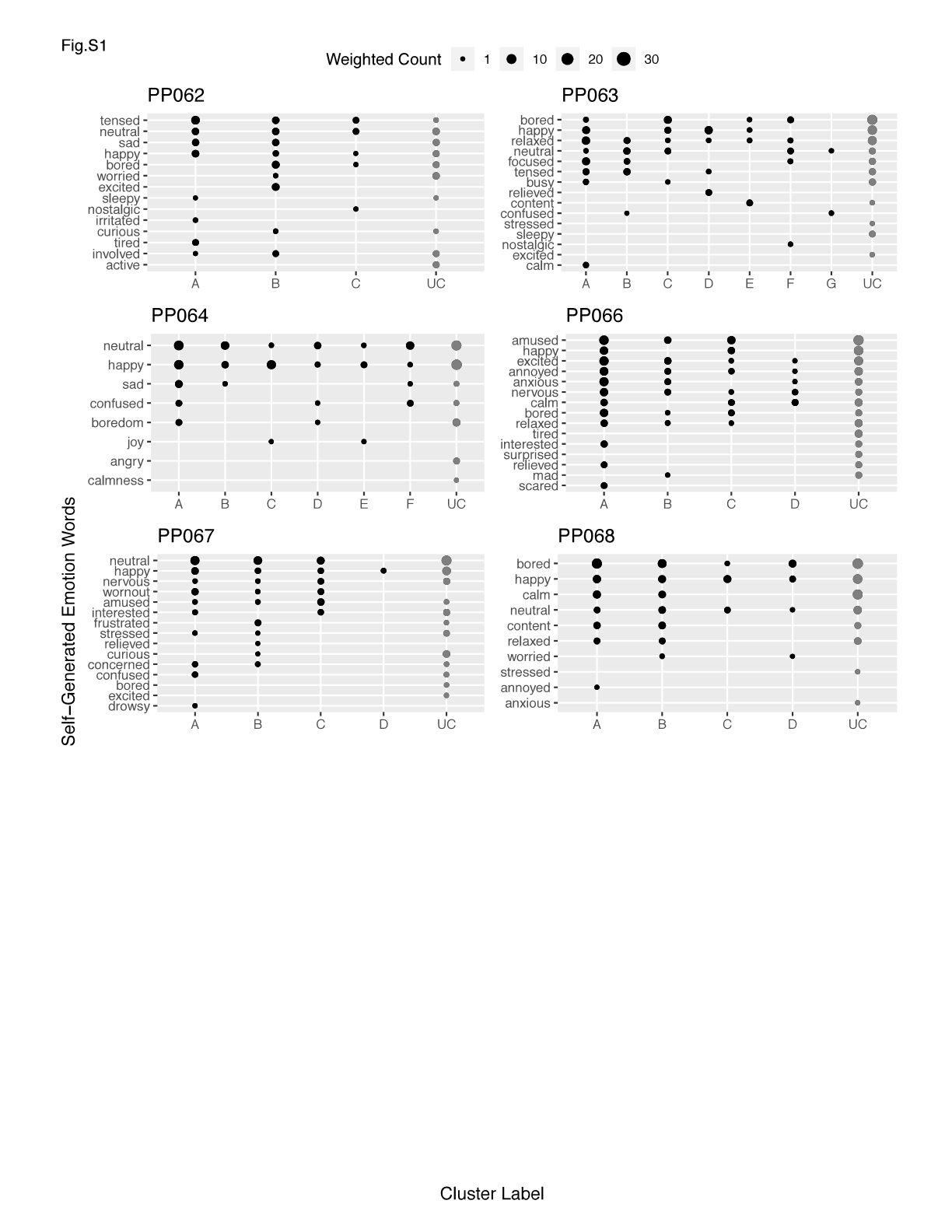

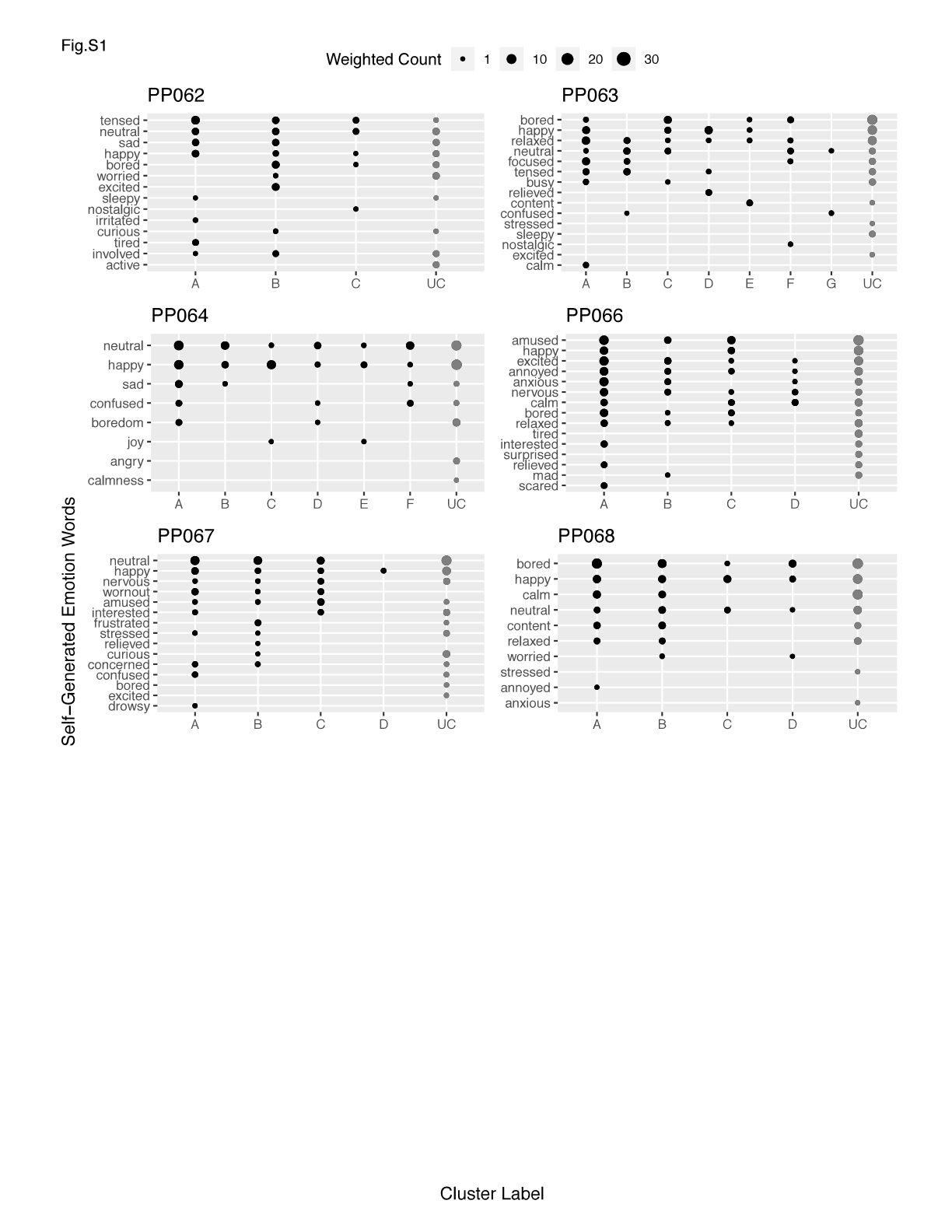


**Figure S4.** Bubble plots of unique words freely generated to label experience sampling events. Words are limited to the 15 most commonly used, and are listed on the vertical axis in descending order of frequency. Clusters based on physiological reactivity are listed on the horizontal axis, included unclustered (“UC”) events (in dark gray) that belong to clusters with less than five percent of events. Bubbles are sized according to the weighted count of events in each cluster labeled by a given word. Specificity of word usage is illustrated by the vertical relationships across words within a cluster. Plots for the six participants excluded from the main analyses due to insufficient usable data are bordered in red.

**Validation of Model Parameters**

Dirichlet Process-Gaussian Mixture Models (DP-GMMs) have a hyperparameter,alpha (i.e., the weight concentration prior in the sklearn package), that indirectly controls the expected minimum number of clusters. Lower values of alpha constrain the model toward finding fewer clusters, while higher values constrain the model less. However, the actual number of clusters discovered by DP-GMM almost always depends on the data, provided that alpha is set to a small enough value and the upper bound for the possible number of clusters is set to a large enough value^34^. Our goal was to avoid tuning parameters for each participant, as this would limit our ability to compare results across participants. Accordingly, we used alpha = (1/N) and an upper bound on number of clusters = N, where N is the number of data points (i.e., vectors of change scores from experience sampling events) submitted to clustering. Setting alpha = (1/ log(N)) corresponds to allowing the model to learn just a single cluster in the data. By selecting a value less than 1/log(N), we effectively constrained the model to discover as few clusters as possible. We did not constrain the model at the upper bound (set to N), as this would have biased the model toward finding fewer clusters and would no longer have been fully data-driven. DP-GMM was developed under the assumption that there could be infinite clusters in the data^34^. By setting the upper bound to N, the only limit we imposed is that the model could not discover more clusters than data points.

To validate our choice of alpha, we used the Bayesian hyperparameter selection approach known as Empirical Bayes ^35^. Approaches such as Bayesian information criterion (BIC) examine penalized maximum likelihood to determine model fit. In contrast, Empirical Bayes is fully Bayesian and examines the marginal likelihood (i.e., evidence) of model fit. Empirical Bayes also avoids having to use frequentist approaches such as cross-validation, which would have been infeasible with the number of data points available per participant. Using an Empirical Bayes approach, we implemented a distribution of possible values for alpha for each participant in our data set, and examined which alpha value resulted in higher marginal likelihood (i.e., greater evidence) of model fit. We observed that values smaller than 1/N (our chosen value) did not meaningfully impact clustering results. Increasing alpha to values greater than 1/N, on the other hand, resulted in larger numbers of clusters with less weight each, and worse marginal likelihood overall. Results for two example participants are illustrated in Figure S5.


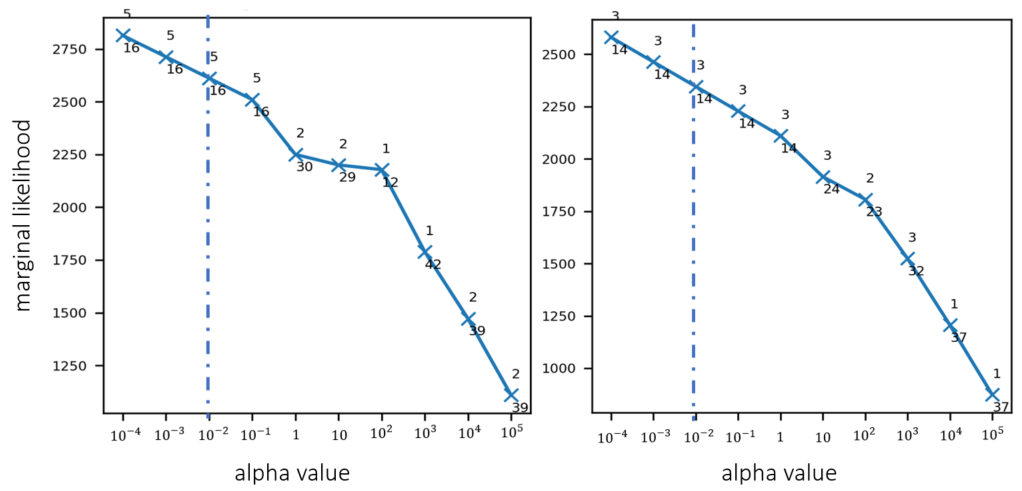


**Figure S5.** Marginal likelihood values (*y*-axis) for different values of the alpha hyperparameter (*x*-axis) for two example participants (015, left; 007, right). The alpha value implemented (1/N) is indicated by the dashed vertical line. Increasing the value of alpha (i.e., moving right on the *x*-axis) results in a drop in marginal likelihood; decreasing (i.e., moving left on the *x*-axis) improves marginal likelihood but does not change the result in any significant way. The numbers at the bottom of each data point along the plotted line reflect the total number of clusters discovered; the numbers at the top reflect the number of clusters retained (i.e., clusters with weights ≥ 0.05). Increasing alpha results in worse solutions (indicated by lower marginal likelihood values) with many small clusters (indicated by larger numbers of discovered clusters).

**Comparison against Standard Gaussian Mixture Modeling**

To further ensure DP-GMM was not producing over-fitted clustering solutions, we compared our approach with a standard mixture model formulation. We implemented a standard Gaussian Mixture Model (GMM) with BIC for each participant in our data set, and set the number of clusters to be between 1 and 10. When we used BIC scores to select the number of clusters, we observed a variable number per participant. Results for two example participants are illustrated in Figure S6.


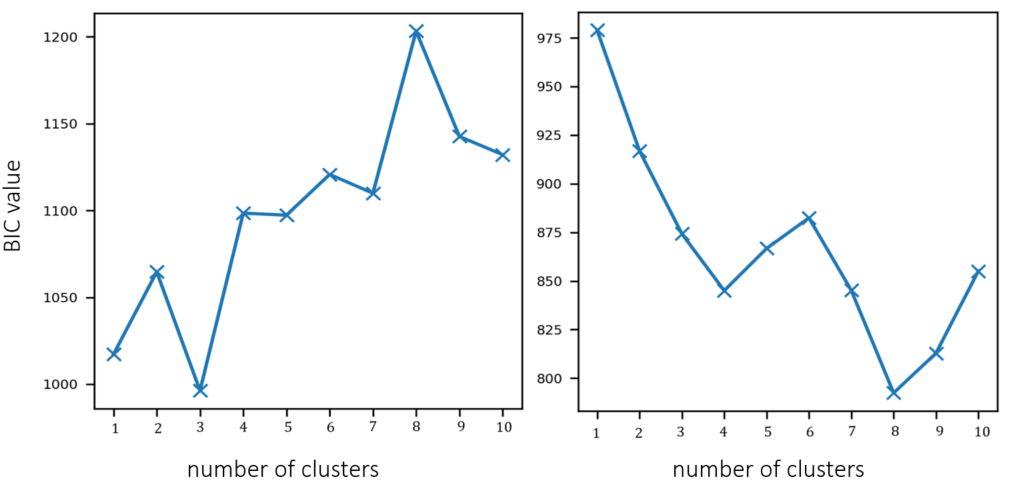


**Figure S6.** Bayesian information criterion (BIC) values (*y*-axis) by number of stipulated clusters (*x*-axis) for two example participants (015, left; 007, right). BIC evaluates model fit while penalizing for model complexity; lower BIC values suggest optimal numbers per participant. *Left panel:* BIC suggests a lower number of clusters (optimally 3). *Right panel:* BIC suggests a higher number of clusters (optimally 8).

As such, our hypothesis that person-specific unsupervised clustering analyses would demonstrate heterogeneity in patterns of change in physiological activity across individuals was supported when using a standard GMM formulation as when using DP-GMM. Our hypotheses about the relationship between patterns of change in physiological activity and mental features of affective experience (i.e., emotion words, self-reported valence and arousal) were also supported when using a standard GMM formulation. As illustrated by the two example participants, we continued to observe a lack of consistency and specificity for emotion word usage (Figure S7) and for ratings of valence and arousal (Figure S8).


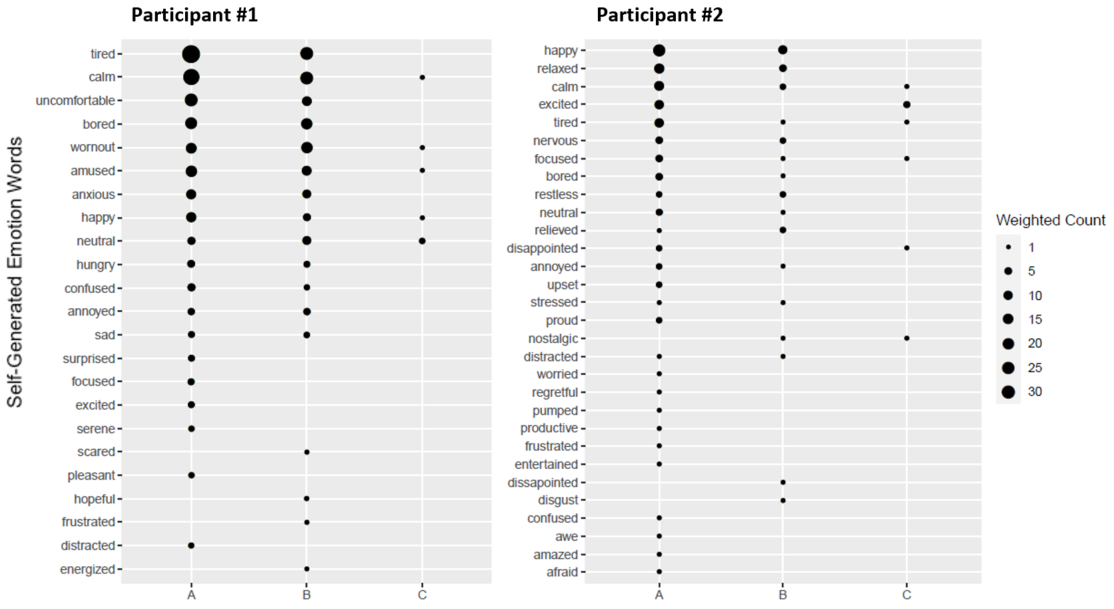


**Figure S7.** Bubble plot of unique words freely generated to label experience sampling events for example participants #1 (left panel) and #2 (right panel). Clusters were discovered using a standard GMM formulation with BIC to select the number of clusters. Words are listed on the vertical axis in descending order of frequency. Clusters of change in physiological activity are shown on the horizontal axis. Bubbles are sized according to the weighted count of events in each cluster labeled by a given word. Specificity of word usage is illustrated when the same word does not appear across different clusters; consistency of word usage is illustrated when few words appear within a single cluster, and when these are different words than appear in other clusters.


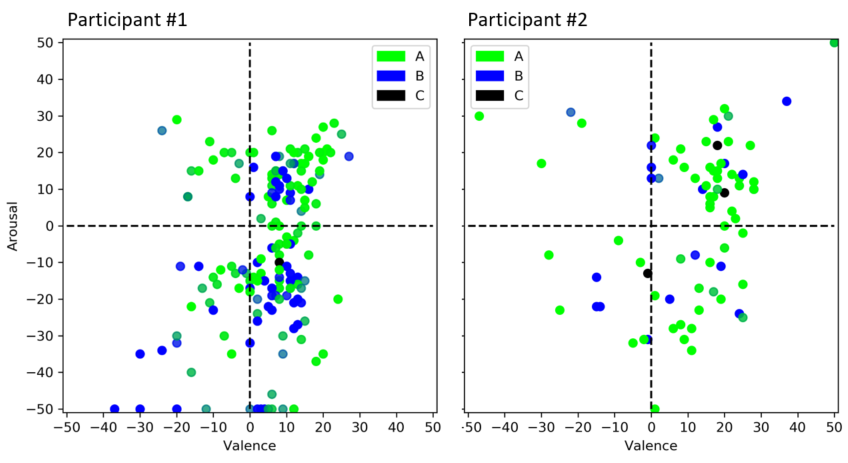


**Figure S8.** Scatter plot of affect ratings reported during experience sampling events for each cluster for example participants #1 (left panel) and #2 (right panel). Clusters were discovered using a standard GMM formulation with BIC to select the number of clusters. Each experience sampling event is plotted as a dot, according to its weighted valence (x-axis) and arousal (y-axis), which are centered on the scale midpoints of 0. Dots are colored according to their highest probability cluster membership, with legends indicating color correspondences.

**References**

1 Barrett, L. F., Adolphs, R., Marsella, S., Martinez, A. & Pollak, S. Emotional expressions reconsidered: Challenges to inferring emotion in human facial movements. *Psychological Science in the Public Interest* **20**, 1-68 (2019).

2 DeSteno, D., Li, Y., Dickens, L. & Lerner, J. S. Gratitude: A tool for reducing economic impatience. *Psychological Science* **25**, 1262-1267 (2014).

3 Kirby, K., Petry, N. & Bickel, W. Heroin addicts have higher discount rates for delayed rewards than non-drug-using controls. *Journal of Experimental Psychology: General* **128**, 78-87 (1999).

4 Beversdorf, D. Q., Hughes, J. D., Steinberg, B. A., Lewis, L. D. & Heilman, K. M. Noradrenergic modulation of cognitive flexibility in problem solving. *Neuroreport* **10**, 2763-2767 (1999).

5 Beversdorf, D. Q., White, D. M., Chever, D. C., Hughes, J. D. & Bornstein, R. A. Central β-adrenergic modulation of cognitive flexibility. *Neuroreport* **13**, 2505-2507 (2002).

6 Fan, J., McCandliss, B. D., Sommer, T., Raz, A. & Posner, M. I. Testing the efficiency and independence of attentional networks. *Journal of Cognitive Neuroscience* **14**, 340-347 (2002).

7 Wilson-Mendenhall, C. D., Barrett, L. F. & Barsalou, L. W. Situating emotional experience. *Frontiers in Human Neuroscience* **7**, 1-16 (2013).

8 Wilson-Mendenhall, C. D., Barrett, L. F. & Barsalou, L. W. Variety in emotional life: Within-category typicality of emotional experiences is associated with neural activity in large-scale brain networks. *Social Cognitive and Affective Neuroscience* **10**, 62-71, doi:10.1093/scan/nsu037 (2015).

9 Russell, J. A. A circumplex model of affect. *Journal of Personality and Social Psychology* **39**, 1161-1178 (1980).

10 Kirschbaum, C., Pirke, K.-M. & Hellhammer, D. H. The ‘Trier Social Stress Test’–a tool for investigating psychobiological stress responses in a laboratory setting. *Neuropsychobiology* **28**, 76-81 (1993).

11 Hout, M. C., Goldinger, S. & Ferguson, R. W. The versatility of SpAM: A fast, efficient, spatial method of data collection for multidimensional scaling. *Journal of Experimental Psychology: General* **142**, 256-281 (2013).

12 Stone, A. A. *et al.* A population approach to the study of emotion: Diurnal rhythms of a working day examined with the Day Reconstruction Method. *Emotion* **6**, 139-149 (2006).

13 Geneva Emotion Research Group. Geneva Appraisal Questionnaire (GAQ). (2002).

14 Scherer, K. R. Appraisal considered as a process of multilevel sequential checking. in *Appraisal Processes in Emotion: Theory, Methods, Research* (eds Scherer, K. R., Schorr, A., & Johnstone, T.) 92-120 (2001).

15 Taylor, S. *et al.* Robust dimensions of anxiety sensitivity: Development and initial validation of the Anxiety Sensitivity Index-3. *Psychological Assessment* **19**, 176-188 (2007).

16 Nock, M. K., Wedig, M. M., Holmberg, E. B. & Hooley, J. M. The emotion reactivity scale: Development, evaluation, and relation to self-injurious thoughts and behaviors. *Behavior Therapy* **39**, 107-116 (2008).

17 Figner, B., Mackinlay, R. J., Wilkening, F. & Weber, E. Affective and deliberative processes in risky choice: Age differences in risk taking in the Columbia Card Task. *Journal of Experimental Psychology: Learning, Memory, and Cognition* **35**, 709-730 (2009).

18 Spitzer, R. L., Kroenke, K., Williams, J. B. & Löwe, B. A brief measure for assessing generalized anxiety disorder: The GAD-7. *Archives of Internal Medicine* **166**, 1092-1097 (2006).

19 Bagby, R. M., Parker, J. D. A. & Taylor, G. J. The twenty-item Toronto Alexithymia Scale—I. Item selection and cross-validation of the factor structure. *Journal of Psychosomatic Research* **38**, 23-32 (1994).

20 Kang, S. M. & Shaver, P. R. Individual differences in emotional complexity: Their psychological implications. *Journal of Personality* **72**, 687-726 (2004).

21 Cohen, S., Kamarck, T. & Mermelstein, R. A global measure of perceived stress. *Journal of Health and Social Behavior*, 385-396 (1983).

22 Kroenke, K., Spitzer, R. L. & Williams, J. B. The PHQ-15: validity of a new measure for evaluating the severity of somatic symptoms. *Psychosomatic Medicine* **64**, 258-266 (2002).

23 Kroenke, K. *et al.* The PHQ-8 as a measure of current depression in the general population. *Journal of Affective Disorders* **114**, 163-173 (2009).

24 Costa, P. T., Jr. & McCrae, R. R. Professional manual: Revised NEO Personality Inventory (NEO-PI-R) and NEO Five-Factor Inventory (NEO-FFI). 101 (Odessa, FL, 1992).

25 Barratt, E. S. Impulsiveness subtraits: Arousal and information processing. *Motivation, Emotion, and Personality* **5**, 137-146 (1985).

26 Weber, E. U., Blais, A. R. & Betz, N. E. A domain‐specific risk‐attitude scale: Measuring risk perceptions and risk behaviors. *Journal of Behavioral Decision Making* **15**, 263-290 (2002).

27 Hughes, M. E., Waite, L. J., Hawkley, L. C. & Cacioppo, J. T. A short scale for measuring loneliness in large surveys: Results from two population-based studies. *Research on Aging* **26**, 655-672 (2004).

28 Zaleskiewicz, T. Beyond risk seeking and risk aversion: Personality and the dual nature of economic risk taking. *European Journal of Personality* **15**, S105-S122 (2001).

29 Nabian, M. *et al.* An open-source feature extraction tool for the analysis of peripheral physiological data. *IEEE Journal of Translational Engineering in Health and Medicine* **6**, 1-11 (2018).

30 Forouzanfar, M. *et al.* Toward a better noninvasive assessment of preejection period: A novel automatic algorithm for B‐point detection and correction on thoracic impedance cardiogram. *Psychophysiology* **55**, e13072 (2018).

31 Cacioppo, J. T. *et al.* Autonomic cardiac control, II: Noninvasive indices and basal response as revealed by autonomic blockades. *Psychophysiology* **31**, 586-598 (1994).

32 Hayano, J. *et al.* Diurnal variations in vagal and sympathetic cardiac control. *The American Journal of Physiology* **258**, H642 (1990).

33 Albers, C. & Lakens, D. When power analyses based on pilot data are biased: Inaccurate effect size estimators and follow-up bias. *Journal of Experimental Social Psychology* **74**, 187-195 (2018).

34 scikit-learn developers. *Gaussian mixture models*, <<https://scikit-learn.org/stable/modules/mixture.html>> (2019).

35 Bishop, C. M. *Pattern Recognition and Machine Learning*. (Springer, 2006).
